# Malignancy and NF-κB signalling strengthen coordination between expression of mitochondrial and nuclear-encoded oxidative phosphorylation genes

**DOI:** 10.1186/s13059-021-02541-6

**Published:** 2021-12-02

**Authors:** Marcos Francisco Perez, Peter Sarkies

**Affiliations:** 1 MRC London Institute of Medical Sciences, Du Cane Road, London, W12 0NN UK; 2 Institute of Clinical Sciences, Imperial College London, Hammersmith Hospital Campus, Du Cane Road, London, W12 0NN UK; 3 Department of Biochemistry, University of Oxford, South Parks Road, Oxford, OX1 3QU UK

## Abstract

**Background:**

Mitochondria are ancient endosymbiotic organelles crucial to eukaryotic growth and metabolism. The mammalian mitochondrial genome encodes for 13 mitochondrial proteins, and the remaining mitochondrial proteins are encoded by the nuclear genome. Little is known about how coordination between the expression of the two sets of genes is achieved.

**Results:**

Correlation analysis of RNA-seq expression data from large publicly available datasets is a common method to leverage genetic diversity to infer gene co-expression modules. Here we use this method to investigate nuclear-mitochondrial gene expression coordination. We identify a pitfall in correlation analysis that results from the large variation in the proportion of transcripts from the mitochondrial genome in RNA-seq data. Commonly used normalisation techniques based on total read counts, such as FPKM or TPM, produce artefactual negative correlations between mitochondrial- and nuclear-encoded transcripts. This also results in artefactual correlations between pairs of nuclear-encoded genes, with important consequences for inferring co-expression modules beyond mitochondria. We show that these effects can be overcome by normalizing using the median-ratio normalisation (MRN) or trimmed mean of *M* values (TMM) methods. Using these normalisations, we find only weak and inconsistent correlations between mitochondrial and nuclear-encoded mitochondrial genes in the majority of healthy human tissues from the GTEx database.

**Conclusions:**

We show that a subset of healthy tissues with high expression of NF-κB show significant coordination, suggesting a role for NF-κB in ensuring balanced expression between mitochondrial and nuclear genes. Contrastingly, most cancer types show robust coordination of nuclear and mitochondrial OXPHOS gene expression, identifying this as a feature of gene regulation in cancer.

**Supplementary Information:**

The online version contains supplementary material available at 10.1186/s13059-021-02541-6.

## Background

Human mtDNA contains 24 genes encoding for ribosomal and transfer RNAs and 13 protein-coding genes, all of which are involved in oxidative phosphorylation (hereafter mtOXPHOS genes). Mammalian mitochondrial genome-encoded RNAs (mtRNAs) are polyadenylated [1] and are thus robustly represented in polyA+ selected RNA-seq libraries, comprising a large fraction of total reads in many human tissues [2]. However, the majority of proteins with mitochondrial localisation are encoded by the nuclear genome, including over 100 genes involved in oxidative phosphorylation (nuOXPHOS genes) in humans.

The mtOXPHOS and nuOXPHOS genes are obligate partners in catalysis within protein complexes with a defined stoichiometry; therefore, their expression should be coordinated in order to maximise cell growth and function. Mitochondrial-to-nuclear communication is known as ‘retrograde signalling’. While retrograde signalling has been characterised in the case of overt mitochondrial function dysfunction or depletion in yeast [3], *Caenorhabditis elegans* [4], *Drosophila* [5] and mammals [6, 7], little is known about whether retrograde signalling functions to co-ordinate mtOXPHOS and nuOXPHOS gene expression under normal physiological conditions.

One possible method to investigate the coordination between mtRNA and nuclear mitochondrial gene expression is to examine the correlation between mtOXPHOS and nuOXPHOS expression across different individuals to investigate whether differences in mtOXPHOS levels are linked to differences in nuOXPHOS expression. Large datasets such as the Genotype Expression Project (GTEx) and the Cancer Genome Atlas (TCGA) are ideal for this purpose, enabling examination of normal and diseased tissues, respectively. Previous approaches utilising this method to examine GTEx data have suggested that there is a weak positive correlation on average between mtOXPHOS and nuOXPHOS expression, indicating coregulation. Surprisingly, however, many tissues were found to have strongly negative correlations [8]. This question has yet to be directly investigated using TCGA data, but the expression of nuclear-encoded mitochondrial genes was shown to correlate, weakly, with predicted mtDNA copy number [9].

An important consideration in the use of gene expression datasets across a number of different samples drawn from different individuals is how to normalize the data to enable comparison across genes and samples. Currently, the dominant technology for high-throughput quantification of gene expression is sequencing RNA (RNA-seq) [10]. The expression level of a gene is indicated by the number of independent sequencing reads mapping to the gene, known as the read count [11]. However, read counts are not comparable for genes within a sample, as longer transcripts accumulate more mapped reads. Read counts are also not comparable for a given gene across samples, due to potential differences in sequencing depth/library size and, crucially, library composition.

A common normalisation method is RPKM (reads per kilobase of the transcript, per million; also known as FPKM for fragments per kilobase of the transcript, per million) for single- or paired-end sequencing, respectively [11]. This method normalizes read counts by library size and gene length. However, the average FPKM still varies from sample to sample, leading to the introduction of the similar TPM (transcripts per million) normalisation [12]. The sum of TPM values across samples is invariant, and thus, TPM is argued to be a better normalisation for the comparison of expression levels across samples [12, 13]. GTEx provides data as TPM, and TCGA provides data as FPKM. Many researchers use data normalized in this way for correlation analysis [8, 14–18], sometimes aided by web-based database interrogation tools [19, 20]. However, other normalisation methods have been proposed which aim to account for biases in library composition, in addition to sequencing depth. The median ratio normalisation (MRN; also known as relative log expression—RLE) provides scaling factors for normalizing library read counts in a way that controls for library size and is insensitive to the presence of a minority of highly expressed transcripts, thus controlling for library composition biases across samples [21]. The trimmed mean of *M* values (TMM) is another algorithm which performs similarly [22]. An alternative normalisation, the upper quartile (UQ) method, adjusts gene read counts by the library 75th percentile of non-zero read counts rather than total read count, so as to exclude the influence of a minority of highly expressed genes [23]. The MRN and TMM normalisation methods are employed in differential expression analysis by the commonly used *R* packages *DESeq2* and *edgeR*, respectively, in which they are used in statistical models.

In this study, we set out to investigate the relationship between mtOXPHOS and nuOXPHOS expression using GTEx and TCGA RNA-seq data, and establish the effects that different RNA-seq normalisation methods may have on this analysis. Further, we aimed to elucidate what might lie behind differences between healthy tissues and tumours in mtOXPHOS-nuOXPHOS coordination.

## Results

### mtOXPHOS-nuOXPHOS gene expression appears anticorrelated using TPM normalisation

To examine the expression correlation between the mtOXPHOS and nuOXPHOS genes, we downloaded RNA-seq data for 54 human tissues from the GTEx website, normalized to TPM (version 8). We excluded tissues with fewer than 100 samples, leaving 48 tissues remaining. We then applied a linear model to each gene to regress out the influence of known confounders (age bracket, sex, cause of death and ischemic time [24] interacting with tissue, as well as sequencing batch and individual donor), continuing downstream analysis with the resulting residuals. We downsampled to 100 samples per tissue to ensure equal representation across tissues. To ensure that the correlations reflect differences among donors and not expression levels between tissues, we ranked the samples for each gene within each tissue, to reflect the relative level of expression within the tissue-specific randomly-sampled cohort of each gene within a sample. Finally, we determined the correlation of the ranks of all pairs of genes using Spearman’s rank-correlation coefficient across the 4800 chosen samples. We repeated this process 100 times and plotted the median value of each correlation coefficient.

**Fig. 1:**
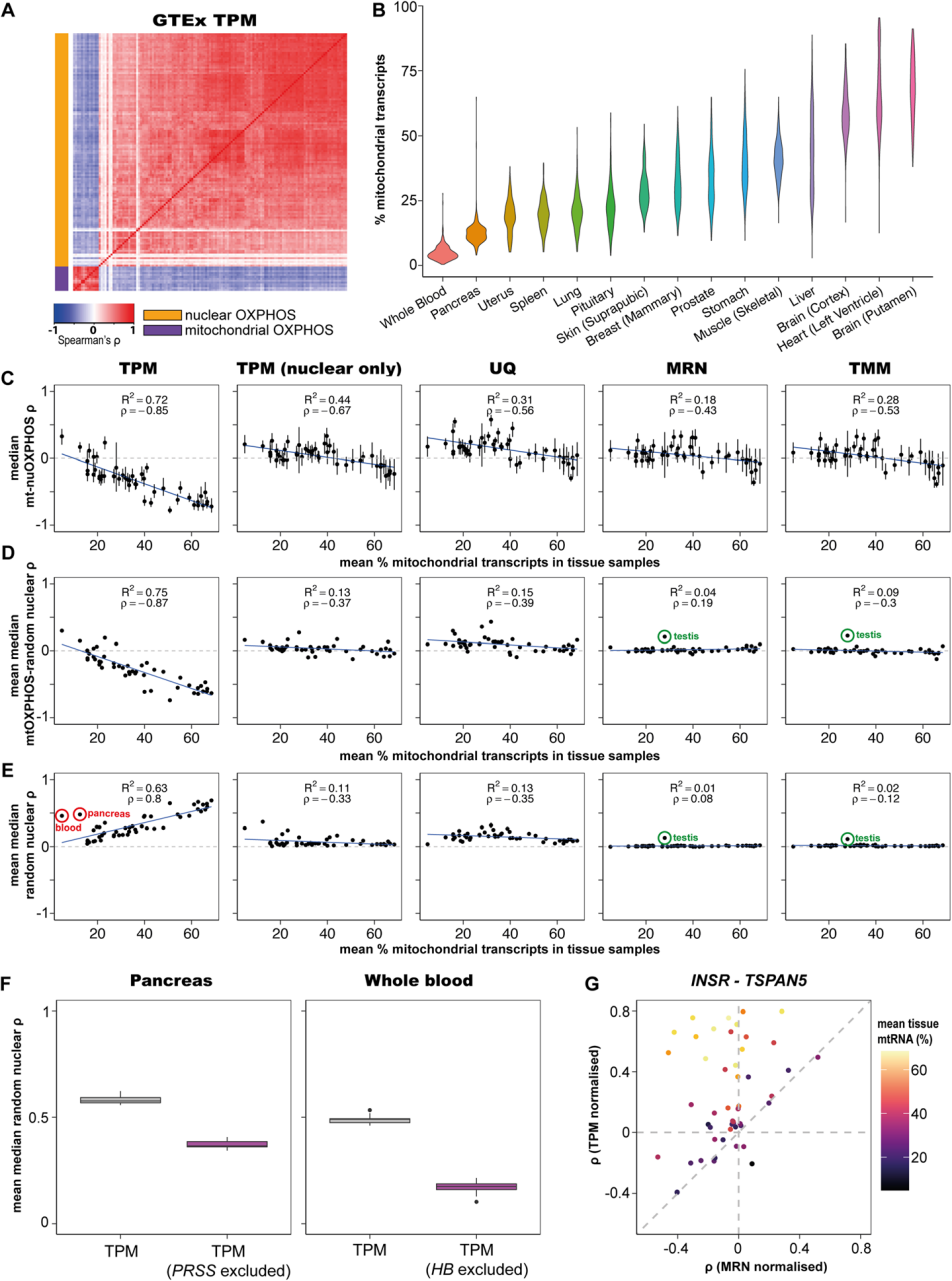
High mitochondrial reads in GTEx libraries lead to artefactual correlations between genes using TPM normalisation, obscuring biological relationships. **A** Heatmap showing median Spearman’s correlation for mtOXPHOS and nuOXPHOS gene expression using TPM normalisation in 48 GTEx tissues combined (100 samples from each tissue, sampled 100 times). The order of genes for this and all mtOXPHOS-nuOXPHOS heatmaps throughout the paper is determined by the hierarchical clustering of correlations from Fig. 2A. **B** Violin plot showing total expression from mtDNA-encoded genes in samples from a selection of 15 healthy human tissues from the GTEx database. **C** Scatterplot shows median Spearman’s correlation (*ρ*) of mtOXPHOS-nuOXPHOS gene pairs within 48 GTEx tissues vs tissue mean of mtRNA levels as a percentage of total transcripts. Shown for data normalized by TPM, TPM excluding mitochondrial reads for normalizing nuclear genes, UQ, MRN and TMM methods. Error bars indicate IQR. Blue line shows linear regression with *R*^2^ and *ρ* noted within panel. **D** Scatterplot shows mean over 100 iterations of the tissue median *ρ* of mtOXPHOS with 126 random expressed nuclear genes vs tissue mean of mtRNA levels as a percentage of total transcripts. Shown for data normalized by TPM, TPM excluding mitochondrial reads for normalising nuclear genes, UQ, MRN and TMM methods. 95% confidence interval error bars are smaller than the plotted symbols. The blue line shows linear regression with *R*^2^ and *ρ* noted within panel. Green circles for MRN and TMM highlight unusually high correlations in the testis. **E** Scatterplot shows mean over 100 iterations of the tissue median *ρ* within 100 random expressed nuclear genes vs tissue mean of mtRNA levels as a percentage of total transcripts. Shown for data normalized by TPM, TPM excluding mitochondrial reads for normalising nuclear genes, UQ, MRN and TMM methods. 95% confidence interval error bars are smaller than the plotted symbols. The blue line shows linear regression with *R*^2^ and *ρ* noted within panel. Red circles for TPM highlight the whole blood and pancreas; green circles for MRN and TMM highlight unusually high correlations in the testis. **F** Boxplot shows mean values for 10 samples of the median *ρ* of 100 random expressed nuclear genes with TPM normalisation or TPM excluding the read counts for *PRSS1* & *PRSS2* (pancreas) or *HBA1*, *HBA2*, *HBB* and *HBD* (whole blood). **G** Scatterplot shows Spearman’s *ρ* within tissues for two genes, *INSR* and *TSPAN5*, for TPM normalized data and MRN normalized data. Colour indicates the tissue mean mtRNA level (% total transcripts). Tissues that fall in the top left quadrant are those in which the negative correlation observed using MRN normalisation has switched sign and appears to be positive using TPM normalisation

Using this approach, expression of mtOXPHOS genes showed a high degree of positive correlation with each other (*ρ* = 0.657), as did nuOXPHOS genes (*ρ* = 0.521). However, mtOXPHOS and nuOXPHOS expression appeared to be strongly anticorrelated (Fig. 1A; *ρ* = −0.376). As nuclear and mitochondrial gene expression have been reported to correlate positively across tissues [2], this result was unexpected and so we set out to investigate the source of the apparent anticorrelation in more depth.

### Variance in mtRNA expression within tissues drives negative mito-nuclear correlations

The average total proportion of transcripts derived from mitochondrial DNA ranges from more than 50% in the heart and most brain tissues to just 4.8% of transcripts in whole blood samples (Fig. 1B; Additional File 1: Fig S1a for all tissues). The variance was also high within tissues, with mtRNAs in the 861 heart (left ventricle) samples ranging from 12.5 to 95.4% of total transcripts (Fig. 1B). As the total of TPM values for each sample must sum to 1 million, a sample with 95.4% mtRNAs leaves fewer than 50,000 transcripts to be shared among the nuclear genome, while a sample with 12.5% mtRNAs has ~875,000. We reasoned that the variation in total mtRNA proportion between donors might therefore dominate the variance in other genes, affecting correlations between genes. Samples with high mtRNA levels will exhibit lower levels of all nuclear genome-derived transcripts and vice-versa, which would result in a spurious negative correlation between mtRNAs and nuclear-encoded RNAs.

For each tissue, we plotted the total percentage of mtRNAs against the median Spearman’s correlation of mtOXPHOS-nuOXPHOS gene pairs, using data for all available samples within each tissue (Fig. 1C). We observed that the average mtRNA proportion was anticorrelated to the average correlation between mtOXPHOS and nuOXPHOS genes, such that high mtRNA proportions led to negative correlations between mtOXPHOS and nuOXPHOS (*ρ* = −0.85 , *p* < 4.33 × 10^-15^).

If this was a result of a technical artefact, we should observe the same pattern when we consider the correlations of mtOXPHOS with randomly selected nuclear genes, instead of the nuOXPHOS genes. Indeed, the median Spearman’s correlation of mtOXPHOS-random nuclear gene pairs also showed a strong negative correlation with total mtRNA percentage (Fig. 1D; (*ρ* = −0.87, *p* < 2.2 × 10^-16^). Thus, as tissue mtRNA levels climb, their variance between samples increasingly comes to dominate the variance of other genes, leading to artefactual negative correlations.

### mtRNA expression variance drives positive correlations between random nuclear transcripts

If sample mtRNA level is a dominant factor in the variance of nuclear gene TPM values, then we reasoned that this might drive artefactual correlations between random nuclear genes which are functionally unrelated to the mitochondria. As the mean tissue mtRNA level rises, variance in mtRNA levels between samples would become an increasingly dominant factor in the variance of nuclear gene TPMs, which are all artificially depressed or inflated in each sample according to mtRNA level.

Supporting this hypothesis, we found that the average correlation for TPM values of pairs of randomly expressed nuclear genes was positive and increased with mtRNA transcript levels (Fig. 1E). The average correlation of random nuclear genes for a tissue was predicted by not only the tissue mean mtRNA level but also the coefficient of variation (CV) of mtRNA levels (linear model, p(mtRNA levels) = 1.37 × 10^-8^ p(CV) = 3.46 × 10^-5^, p(mtRNA levels * CV) = 0.0136). Stronger artefactual correlations are therefore produced when analysing tissues with higher and more variable mtRNA levels.

We noticed two outlying tissues with low mtRNA levels and high correlations between random nuclear genes (Fig. 1E; red circles)—the whole blood and pancreas. We looked at the highest expressing genes in these tissues to see if these correlations might be driven by different library composition biases. In whole blood samples, just 4 genes encoding haemoglobin subunits—*HBB*, *HBA2*, *HBA1* and *HBD*—make up 40.8% of transcripts on average. Meanwhile in pancreas samples, there is high expression of many digestive enzymes, with two paralogous genes encoding serine proteases—*PRSS1* and *PRSS2*—comprising an average of 18.4% of transcripts. Removing the read counts for these genes from whole blood and pancreas samples before computing sample sums for TPM normalisation led to large and significant decreases in correlation coefficients between random genes (Fig. 1F). This shows that the effect we observe is not specific for mitochondrial transcripts but rather applies generally for any transcripts that tend to comprise a high proportion of the total RNA-seq read count.

The inflation of correlations using TPM normalisation is a problem for all tissue datasets, but much more so for those with a high mean mtRNA level (or other strong library composition bias), leading in some cases to gene pairs with true negative correlations switching sign and giving the appearance of being significantly and robustly co-expressed (Fig. 1G).

Notably, all these analyses were entirely consistent when using Pearson’s correlations instead of Spearman’s rank correlation coefficient (Additional File 1: Fig S1). Similar artefactual correlations between mito-nuclear gene pairs or pairs of random nuclear genes were also found using data subjected to FPKM normalisation instead of TPM (Additional File 1: Fig S2).

### Normalizing libraries with MRN or TMM scaling factors abrogates correlation artefacts

We sought to determine if alternative normalisation methods could mitigate these artefacts during correlation analysis. We tried four alternative normalisations. First, we tried removing the mtRNA reads when computing sample read sums for the purposes of normalising nuclear gene expression as with TPM (mtRNAs were still normalized by the total sample sums). We also tried UQ normalisation, which is already applied to some pre-normalized expression datasets available from TCGA. Lastly, we employed MRN and TMM algorithms, which use different procedures to arrive at a scaling factor for library normalisation that is designed to be insensitive to library composition bias.

When computing TPM values for nuclear genes with mitochondrial reads excluded, we find that the strong negative correlation of tissue mitochondrial level with average mtOXPHOS-nuOXPHOS was successfully mitigated (Fig. 1C). Indeed, there was no relationship between tissue mtRNA level with average mtOXPHOS gene-random nuclear correlations (Fig. 1D) or average correlations between random nuclear genes (Fig. 1E). However, in the tissues with different sources of library composition biases such as the whole blood or pancreas, average correlations of random nuclear genes were still strongly positive, while they remained at least slightly positive in all tissues. Excluding mitochondrial reads alone therefore fails to account for all library composition biases.

UQ normalisation performed similarly to TPM with mitochondrial reads excluded with respect to artefacts driven by high mitochondrial transcript levels (Fig. 1C–E) and abrogated the strong positive correlation between random nuclear genes driven by alternative library composition biases in whole blood and pancreas samples (Fig. 1E). However, UQ normalisation resulted in a stronger inflation of correlations between random nuclear genes in most tissues (Fig. 1E) and therefore cannot be recommended for performing correlation analyses.

MRN and TMM normalisations abrogated the artefactual correlations of mitochondrial genes with random nuclear genes (Fig. 1D) and between random nuclear genes (Fig. 1E). We conclude that scaling read counts by library scaling factors produced by MRN or TMM algorithms is a simple and appropriate way of normalising RNA-seq data before correlation analysis. We note that the processed data used for QTL analysis available for download on the GTEx portal are already TMM-normalized and thus may be appropriate for use in correlation analysis (note however that in further processing these values have been corrected for biological as well as technical variance and so may not be suitable for all analyses).

We note that one tissue, the testis (green circles, Fig. 1D, E; Additional File 1: Fig S1C, D), displayed positive correlations between mtOXPHOS genes and random nuclear genes and also between pairs of random nuclear genes when using MRN and TMM normalisations. The distribution of correlations across random nuclear gene pairs differed in width across tissues (Additional File: Fig S3A); in some tissues, more extreme positive and negative correlations were more common, which was apparent when inspecting correlation heatmaps between random nuclear genes (Additional File: Fig S3B). Nonetheless, the distributions were centred around 0 and were unimodal for all tissues but the testis (Additional File: Fig S3A), which displayed a significantly bimodal distribution of correlations between gene pairs for 7/100 samples of 100 random nuclear genes (Hartigan’s dip test; FDR < 0.05; no significant samples in any of the 47 other tissues). This unusual property may be related to the unique transcriptional complexity of the testis, with over 80% of protein-coding genes expressed [25].

### Correlations of mtOXPHOS-nuOXPHOS genes are weak and inconsistent across tissues

**Fig. 2:**
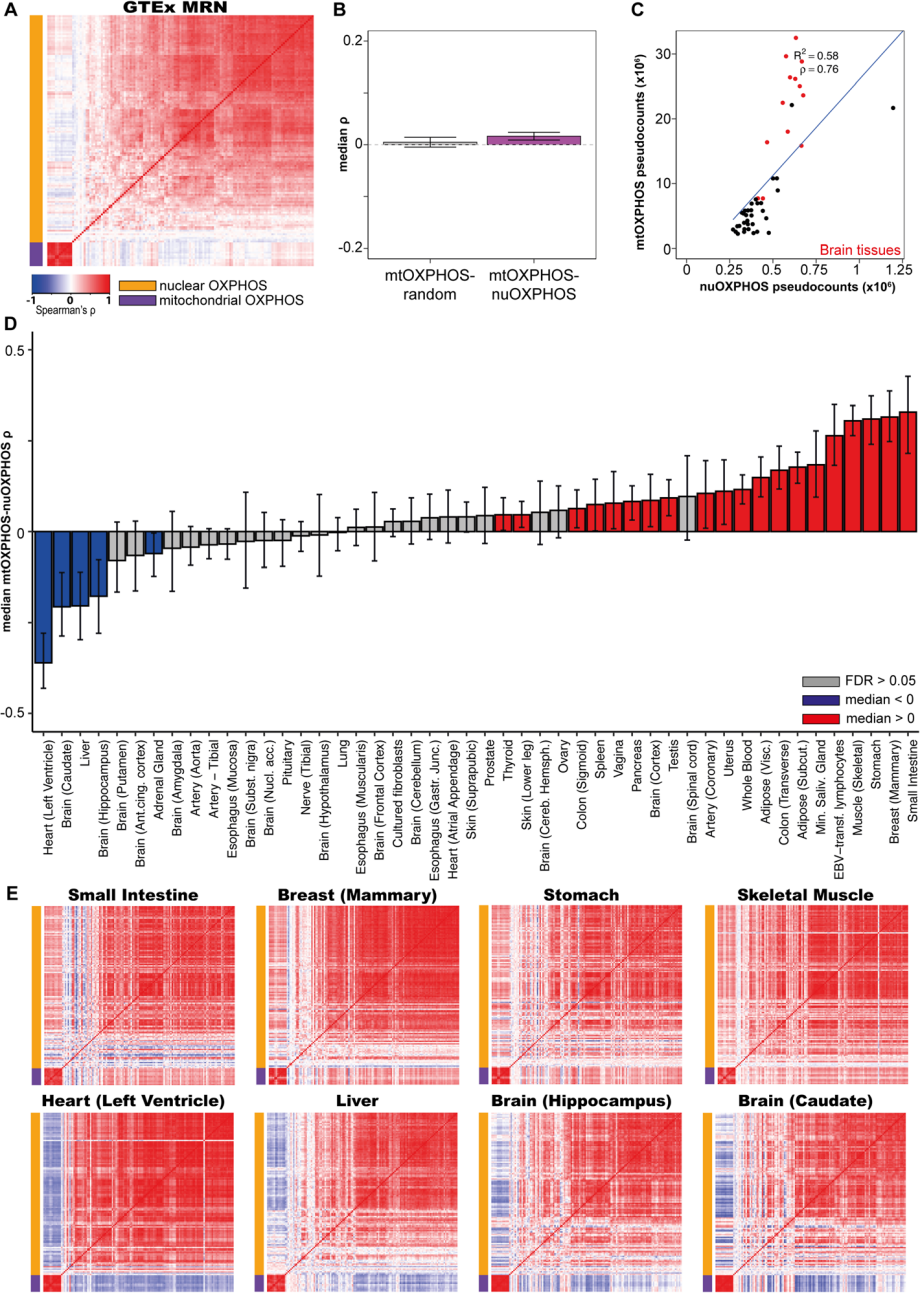
MRN normalisation reveals weak and inconsistent correlations between mtOXPHOS and nuOXPHOS within tissues. **A** Heatmap showing median Spearman’s *ρ* for mtOXPHOS and nuOXPHOS gene expression using MRN normalisation in 48 GTEx tissues combined (100 samples from each tissue, sampled 100 times). **B** Boxplot showing median Spearman’s *ρ* for 100 iterations of mtOXPHOS genes with random nuclear genes or mtOXPHOS with nuOXPHOS genes for 48 GTEx tissues combined. **C** Scatterplot showing mean MRN-normalized pseudocounts for mtOXPHOS and nuOXPHOS genes in 48 GTEx tissues. The 13 brain tissues display a high mtOXPHOS count and are shown in red. **D** Observed median Spearman’s *ρ* between mtOXPHOS and nuOXPHOS genes for 48 GTEx tissues. Error bars show 95% bootstrap confidence interval. Blue bars indicate observed correlation significantly lower than 0 (bootstrap empirical *p* value, FDR-adjusted < 0.05), red bars indicate observed correlation significantly higher than 0 and grey bars indicate FDR > 0.05. *Z* statistics and *p* values for observed value relative to mtOXPHOS with random nuclear genes are shown in Additional File 1:Table S1. **E** Heatmap showing Spearman’s *ρ* for mtOXPHOS and nuOXPHOS genes for 8 GTEx tissues showing clear positive or negative correlations

Having established appropriate normalisations for the data, we returned to the question of the correlation of the mtOXPHOS and nuOXPHOS genes. We normalized GTEx read counts using MRN or TMM across all tissues and regressed out the influence of confounding variables as above. We sampled 100 samples from each tissue, ranked the residuals for each gene within the tissue sample and aggregated these ranks across tissues before computing the Spearman’s correlation across 4800 samples. We repeated this process 100 times and plotted the median coefficient of the 100 iterations for each gene pair (Fig. 2A).

After MRN or TMM normalisation, the overall correlation between mtOXPHOS and nuOXPHOS genes between samples in the GTEx data was very weak. The mean median Spearman’s correlation for mtOXPHOS-nuOXPHOS gene pairs was only slightly higher than the mean median Spearman’s correlation between mtOXPHOS genes and random nuclear genes (0.0166 vs 0.00488 for MRN normalisation; −0.0120 vs −0.0315 for TMM normalisation; Fig. 2B). Although significant (*t* test *p* = 3.13 × 10^-18^ for MRN; 5.58 × 10^-30^ for TMM), only a tiny fraction of the variation in nuOXPHOS gene expression could be explained by mtOXPHOS gene expression. This suggests that despite broad coordination of mtOXPHOS-nuOXPHOS expression across tissues (Fig. 2C) [2], there is limited coordination that can be detected between the nuclear and mitochondrial gene expression programs within tissues. Notably, this contrasts to very clear coregulation within the mtOXPHOS (MRN, *ρ* = 0.859; TMM, *ρ* = 0.847) and nuOXPHOS (MRN, *ρ* = 0.351; TMM, *ρ* = 0.407) gene sets.

With MRN normalized data, we observed that the relationship between mtOXPHOS and nuOXPHOS gene expression in individual tissues is inconsistent (Fig. 2D, Additional File 1: Table S1, Additional File 2). 23/48 tissues exhibited a median mtOXPHOS-nuOXPHOS correlation that was not significantly different from 0 (bootstrap *p* value, FDR > 0.05). 20/48 tissues exhibited a median mtOXPHOS-nuOXPHOS correlation significantly higher from 0, the most striking being the small intestine (terminal ileum), breast (mammary tissue), stomach and skeletal muscle all with median correlations between 0.30 and 0.32 (FDR < 0.003; Fig. 2E). 5/48 tissues displayed a correlation significantly lower than 0; the left ventricle of the heart, liver, hippocampus (brain), caudate (basal ganglia of the brain) and adrenal gland, with medians −0.361, −0.204, −0.206, −0.177 (all FDR < 0.003) and −0.0606 (FDR = 0.0349), respectively (Fig. 2E). Although the testis has a positive mtOXPHOS-nuOXPHOS correlation, it was significantly lower than that observed between mtOXPHOS and random nuclear genes (Additional File 1: Fig S4A & Table S1), which was unusually high as noted above. Similar results were obtained starting with a TMM normalisation, albeit with 17 significantly positive tissues and 9 significantly negative tissues (Additional File 1: Fig S5).

We were concerned that additional confounders, namely genetic ancestry and the cell type composition of different samples, might influence these conclusions. We repeated the single-tissue mtOXPHOS-nuOXPHOS expression correlation analysis with MRN normalisation as above, but additionally regressing out the top 5 genotyping principal components and estimated sample cell type compositions [26]. For the 27 tissues for which these estimates were available, the resulting median mtOXPHOS-nuOXPHOS correlations were largely unchanged and spread across a similar range (Additional File 1: Fig S6). While some tissues gain or lose significant correlations, no tissues significantly switch signs and the estimates from one analysis are not significantly different from the estimates in the other in any case. As such, we conclude that these additional confounding variables do not strongly influence our analysis.

### Positive correlation between mtOXPHOS and nuOXPHOS gene expression in cancer

We next explored whether any relationship between mtOXPHOS and nuOXPHOS gene expression exists in cancer samples. We downloaded data from TCGA (harmonised) for 31 cancer types with more than 50 primary tumour samples.

**Fig. 3:**
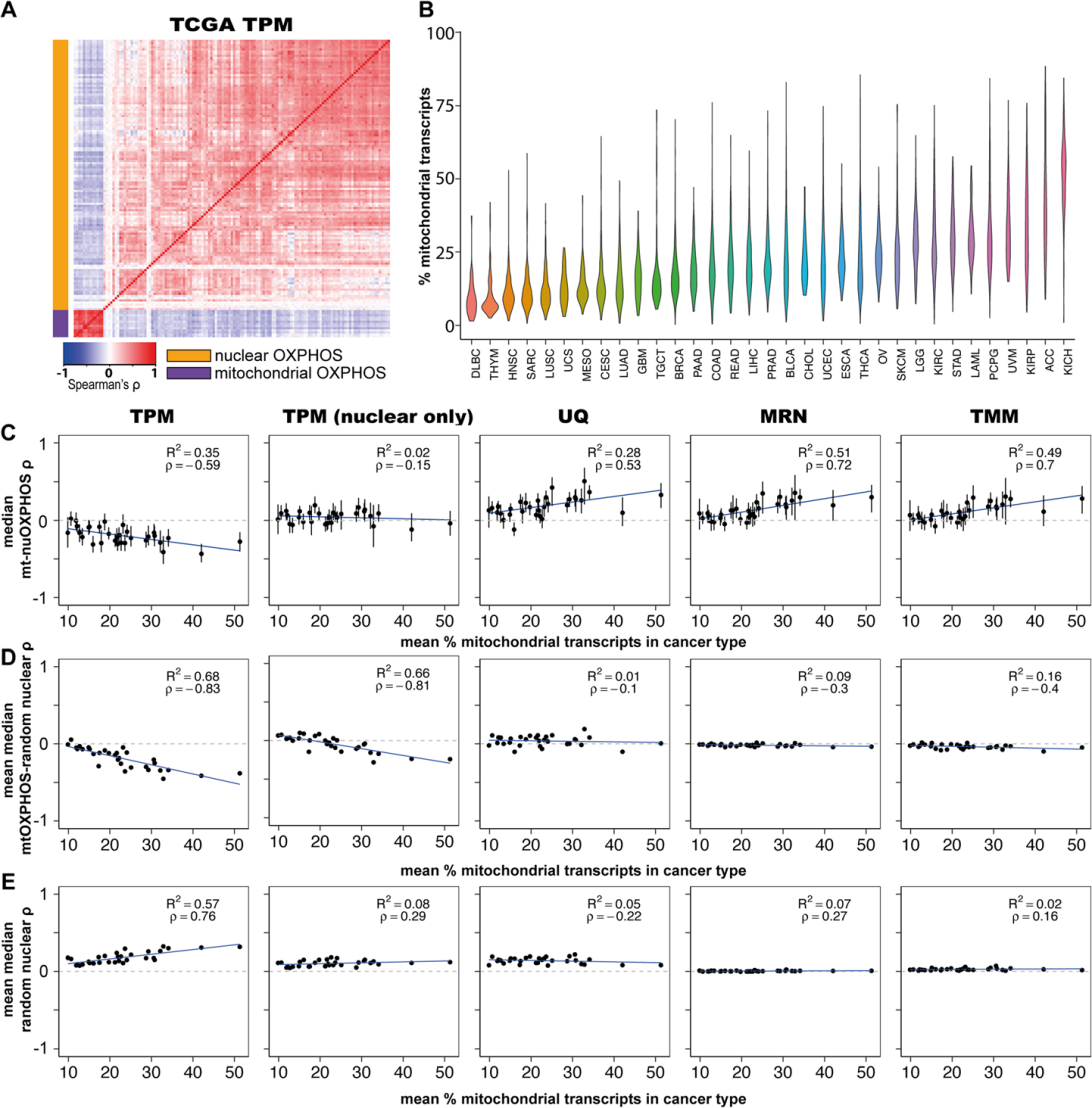
Artefactual correlations are also driven by mtRNA expression in the Cancer Genome Atlas (TCGA) database. **A** Heatmap showing median Spearman’s correlation for mtOXPHOS and nuOXPHOS gene expression using TPM normalisation in 31 TCGA cancer types combined (50 samples from each tissue, sampled 100 times). **B** Violin plot showing total expression from mtDNA-encoded genes in samples from 33 cancer types from TCGA database. **C** Scatterplot shows median Spearman’s correlation (*ρ*) of mtOXPHOS-nuOXPHOS gene pairs within 31 TCGA cancer types vs cancer type mean of mtRNA expression as a percentage of total transcripts. Shown for data normalized by TPM, TPM excluding mitochondrial reads for normalising nuclear genes, UQ, MRN and TMM methods. Error bars indicate IQR. Blue line shows linear regression with *R*^2^ noted within panel. **D** Scatterplot shows mean over 100 iterations of the cancer type median *ρ* of mtOXPHOS with 126 random expressed nuclear genes vs cancer type mean of mtRNA expression as a percentage of total transcripts. Shown for data normalized by TPM, TPM excluding mitochondrial reads for normalising nuclear genes, UQ, MRN and TMM methods. 95% confidence interval error bars are smaller than the plotted symbols. The blue line shows linear regression with *R*^2^ and *ρ* noted within panel. **E** Scatterplot shows mean over 100 iterations of the cancer type median *ρ* within 100 random expressed nuclear genes vs cancer type mean of mtRNA expression as a percentage of total transcripts. Shown for data normalized by TPM, TPM excluding mitochondrial reads for normalising nuclear genes, UQ, MRN and TMM methods. 95% confidence interval error bars are smaller than the plotted symbols. The blue line shows linear regression with *R*^2^ and *ρ* noted within panel. Abbreviations for cancer type are given in Additional File 1: Table S5

We observed substantial variation in mtRNA expression across and within cancer types (Fig. 3B). As we observed for GTEx data, normalisation of TCGA gene expression values using TPM led to spurious correlations, both between mtOXPHOS-nuOXPHOS genes and random nuclear gene pairs (Fig. 3A, C–E). We then proceeded to apply MRN normalisation and repeated the analysis. These normalisation methods corrected the spurious correlations between mtOXPHOS genes and random nuclear genes and between pairs of random nuclear genes (Fig. 3A, D, E).

**Fig. 4:**
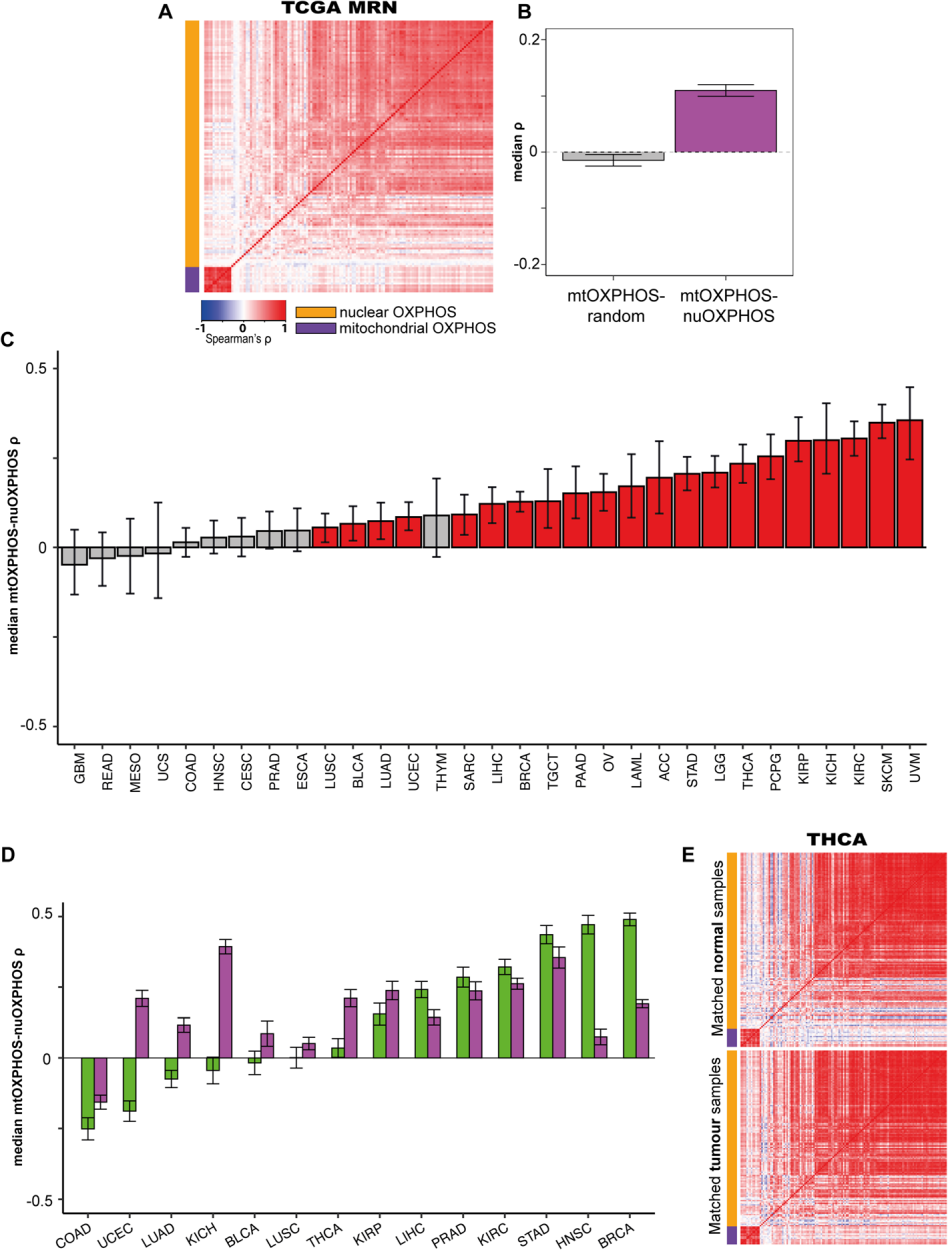
Most cancer types display a positive correlation between mtOXPHOS and nuOXPHOS expression. **A** Heatmap showing median Spearman’s ρ for mtOXPHOS and nuOXPHOS gene expression using MRN normalisation in 31 TCGA cancer types combined (50 samples from each tissue, sampled 100 times). **B** Boxplot showing median Spearman’s *ρ* for 100 iterations of mtOXPHOS genes with random nuclear genes or mtOXPHOS with nuOXPHOS genes for 31 TCGA cancer types combined. **C** Observed median Spearman’s *ρ* between mtOXPHOS and nuOXPHOS genes for 31 TCGA cancer types. Error bars show bootstrap 95% confidence interval. The blue bar indicates observed correlation significantly lower 0 (bootstrap empirical *p* value, FDR-adjusted < 0.05), red bars indicate observed correlation significantly higher than 0 and grey bars indicate FDR > 0.05. *Z* statistics and *p* values for observed value vs mtOXPHOS-random nuclear correlations are shown in Additional File 1: Table S2. **D** Median mtOXPHOS-nuOXPHOS *ρ* within matched samples of tumour (purple) or normal tissue (green) from TCGA projects. Error bars show bootstrapped standard error. **E** mtOXPHOS-nuOXPHOS correlation heatmaps for thyroid cancer (TCGA) matched normal tissue samples (above) and tumour samples (below). Abbreviations for cancer types are given in Additional File 1: Table S5

Nevertheless, with MRN normalisation 21 of 31 individual cancer types tested displayed a significantly positive correlation (FDR < 0.05), with none displaying a significant negative correlation (Fig. 4C, Additional File 1: Table S2; Additional File 3). Median correlations of mtOXPHOS gene expression with random nuclear genes were around 0 for all cancer types (Additional File 1: Fig S4B). Analysing across cancer types, the mean median Spearman’s correlation for mtOXPHOS-nuOXPHOS genes was 0.110, while for mtOXPHOS with random nuclear genes it was −0.0147 (Fig. 4A, b; *t* test *p* value = 5.53 × 10^-158^). In contrast, the mean median correlations within the mtOXPHOS genes (*ρ* = 0.781) and nuOXPHOS genes *(ρ* = 0.314) were similar to those observed in the GTEx (*ρ* = 0.859 & 0.351, respectively). Interestingly, the degree of mtOXPHOS-nuOXPHOS correlation within a cancer type increased with mean mtRNA expression level (Fig. 3C), in a reversal of the apparent relationship when using TPM-normalized data.

We tested whether sample cell type composition strongly influences these results, as we had for the GTEx data. We corrected MRN-normalized gene expression values with cancer type-specific molecular subtypes computed by DeClust, a gene expression deconvolution tool [27], for 13 TCGA cancer types. We found that although one cancer type (UCEC) lost its significant positive correlation, the results were similar to those found without molecular subtype classification (Additional File 1: Fig S7). We conclude that sample cell type composition is not a major confounder of our analysis.

We analysed 14 cancer types for which data for at least 10 normal adjacent tissue samples were available and identified the matched tumour counterpart samples. Using MRN-normalized data, we then computed the correlations between mtOXPHOS and nuOXPHOS genes within the normal and tumour samples to compare them (Fig. 4D). Direct comparison between tissues for the two databases is not possible, as healthy tissue samples from the TCGA are taken from an area in close proximity to the tumour and do not necessarily derive from the same tissue within the organ as their apparent GTEx counterparts. That said, we note that the mtOXPHOS-nuOXPHOS correlations we observed for normal samples in TCGA were broadly congruous with those observed in the GTEx tissues, in particular the strong positive correlations in TCGA adjacent normal tissue samples from the stomach (STAD) and breast (BRCA). We found that the mtOXPHOS-nuOXPHOS correlation tended to be positive in diseased samples in those tissues in which the normal cohort displayed a negative or nonsignificant correlation, such as in thyroid cancer (Fig. 4E; Additional Files 4-5). Overall, 13/14 matched tumour samples displayed a significantly positive mtOXPHOS-nuOXPHOS correlation, compared to 7/14 of the matched normal tissue samples (*p* = 0.0329, Fisher’s exact test).

### NF-κB expression is correlated with OXPHOS coordination in healthy tissues

To understand what might underlie the variation in mtOXPHOS-nuOXPHOS correlation across tissues, we correlated the mean expression of all genes within each GTEx tissue to the observed tissue OXPHOS correlation. We selected the 1000 genes whose expression across tissues (tissue mean expression) most strongly positively correlated to mtOXPHOS-nuOXPHOS correlation across 48 tissues (the top 1000, Spearman’s *ρ* > 0.487, FDR < 0.0210) and performed a gene ontology and KEGG pathway enrichment analysis; we did the same for the 1000 most anticorrelated genes (the bottom 1000, Spearman’s *ρ* < −0.443, FDR < 0.0325). While the anticorrelated genes yielded no significantly enriched terms, the positively correlated genes were strongly enriched for terms related to immune function and B cell activation (Additional File 1: Fig S8A, B; Additional Files 6-7). On closer inspection, we found that this was mostly due to a large number (221) of immunoglobulin-encoding genes within the top 1000 genes. These immunoglobulin genes represented all three major immunoglobulin clusters: IGH (107 genes), IGK (50) and IGL (42), as well as IGH (15) and IGK (7) orphons located outside the main clusters. As a control, we performed the same analysis with the 1000 genes whose expression correlated most positively to mtOXPHOS-random nuclear correlation across tissues; no enriched terms were observed.

We used estimates of immune cell fraction for GTEx samples produced by GEDIT, a gene expression deconvolution tool [28] to see if this was predictive of nuOXPHOS-mtOXPHOS correlation. Immune cell fraction could explain little of the OXPHOS correlation variance (*R*^2^ = 0.0199, *p* < 1.57 × 10^-77^) (Additional File 1: Fig S9A).

**Fig. 5:**
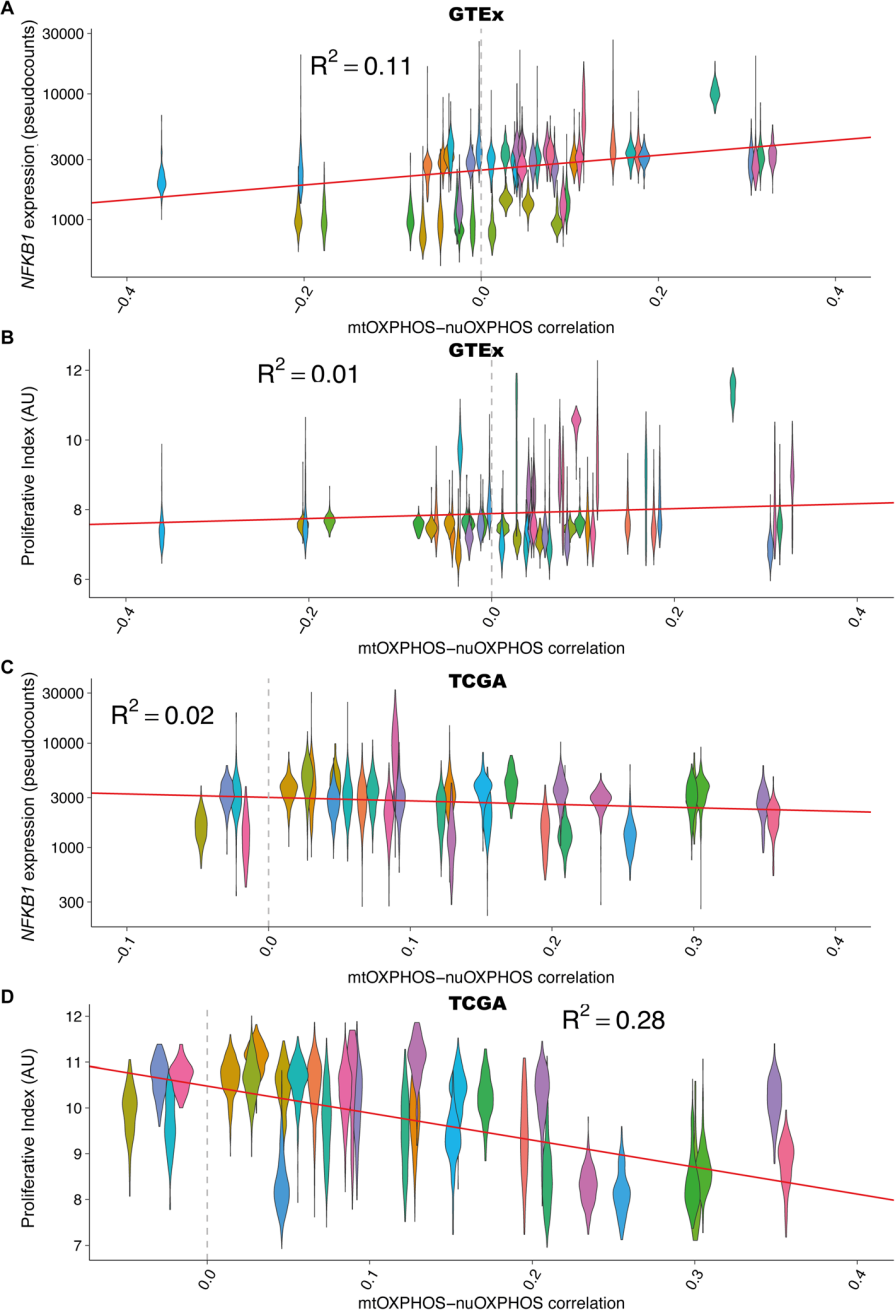
mtOXPHOS-nuOXPHOS coordination is correlated to NF-κB expression and cellular proliferation in the GTEx and TCGA, respectively. **A** Within-tissue mtOXPHOS-nuOXPHOS correlation against *NFKB1* expression (MRN pseudocounts) for GTEx tissues. Each violin represents the samples within a tissue. **B** Within-tissue mtOXPHOS-nuOXPHOS correlation against Proliferative Index for GTEx tissues. Each violin represents the samples within a tissue. **C** Within-tissue mtOXPHOS-nuOXPHOS correlation against *NFKB1* expression (MRN pseudocounts) for TCGA cancer types. Each violin represents the samples within a tissue. **D** Within-tissue mtOXPHOS-nuOXPHOS correlation against Proliferative Index for TCGA cancer types. Each violin represents the samples within a tissue. Dashed grey vertical line shows no correlation. AU, arbitrary units. Also see Additional File 1: Fig S10 for nonparametric analysis

In addition to immune-related terms, there was an enrichment for terms that implied a possible role for cellular proliferation, such as ribosome biogenesis and DNA replication. To assess this contribution to mtOXPHOS-nuOXPHOS correlation, we used the expression data to infer a measure of active proliferation, the Proliferative Index (PI) [29], for all GTEx samples. Despite a significant relationship, the PI explained a tiny fraction of the variance across tissues in OXPHOS correlation (*R*^2^ = 0.00759, *p* < 1.22 × 10^-30^; Fig. 5B, Additional File 1: Fig S10B).

Another possibility was that immunoglobulin expression was driven by NF-κB, a protein complex present in almost all cell types that can drive immunoglobulin expression in non-immune cells [30] and has been implicated in mitochondrial signalling to the nucleus in mammals [31] and in mitochondrial gene regulation [32]. Three of the 5 NF-κB members (*NFKB1*, *REL* and *RELB*) were found in the top 1000 genes. Indeed, there was a strong correlation between tissue *NFKB1* expression level and total expression from *IG* genes (Additional File: Fig S9B-E). Together the expression of the 5 NF-κB members explain much of the variance in mtOXPHOS-nuOXPHOS correlation, with *NFKB1* expression alone the most important contribution (Fig. 5A, Additional File 1: Fig S10B & Table S3). Sample *NFKB1* expression alone explains 10.7% of variation in OXPHOS coordination (*p* = 0.0306 compared to random nuclear genes).

### Proliferation is negatively correlated to OXPHOS coordination in cancer

Having established the importance of NF-κB expression in OXPHOS coordination in healthy tissues, we next tested whether NF-κB could play a similar role in cancer. However, we found only a weak, negative relationship between *NFKB1* expression and mtOXPHOS-nuOXPHOS correlation in the TCGA data (*R*^2^ = 0.0194, *p* < 1.34 × 10^-43^; Fig. 5C, Additional File 1: Fig S10B).

We next correlated the mean expression of all genes within 31 TCGA cancer types to the observed mtOXPHOS-nuOXPHOS correlation, before performing a gene ontology enrichment analysis on the top and bottom 1000 genes (Spearman’s *ρ* > 0.467, FDR < 0.196 and *ρ* < −0.502, FDR < 0.160, respectively; Additional Files 8-9). Overall, there was no overlap at all between the gene ontology terms that were positively or negatively related to OXPHOS coordination in both GTEx and TCGA databases. For the TCGA, the top 1000 genes yielded only 7 enriched terms, related to the lysosome and Golgi apparatus. On the contrary, the 1000 most negatively correlated genes were associated with 220 significant terms, many associated with replication and mitosis (Additional File 1: Fig S11). While control analyses using the top and bottom 1000 genes correlating to mtOXPHOS-random nuclear correlation did yield a few significantly enriched terms (6 for top 1000, 30 for bottom 1000 over 4 gene set libraries), there was no overlap with terms enriched for genes correlating with mtOXPHOS-nuOXPHOS correlation.

In agreement with the enrichment of proliferation-related terms in the bottom 1000 genes, we found a strong negative relationship between Proliferative Index and mtOXPHOS-nuOXPHOS coordination (*R*^2^ = 0.279, *p* < 2.2 × 10^-16^; Fig. 5D; Additional File 1: Fig S10B & Table S4), suggesting that variation in the rate of proliferation in cancer might explain differences in OXPHOS coordination (see the “Discussion” section).

## Discussion

Here we investigated the coordination between the expression of mitochondrial and nuclear transcripts, in particular the mtOXPHOS genes which comprise all 13 mitochondrially encoded protein-coding genes and their nuclear-encoded catalytic partners, the nuOXPHOS genes. Using tens of thousands of RNA-seq samples across 48 healthy human tissues from the GTEx database and 31 distinct cancer types from TCGA, we examined the correlation of mtOXPHOS and nuOXPHOS expression to identify signs of co-regulation. We found that most healthy tissues in the GTEx database showed little correlation between mtOXPHOS and nuOXPHOS expression. In contrast, in TCGA we find that cancers showed a clear tendency towards positive correlations between mtOXPHOS and nuOXPHOS expression. Our results have implications for the analysis of gene expression coordination as well as the potential for retrograde signalling to balance the nuclear and mitochondrial expression of genes with mitochondrial function.

### Avoiding artefacts when analysing correlations between expressed genes

In the course of our investigation, we discovered that the apparent relationship between mtOXPHOS and nuOXPHOS expression depends strongly on the choice of normalisation method used for RNA-seq data. We showed that this is due to the fact that mtRNA levels, including mtOXPHOS genes, make up more than 50% of the total sequenced transcripts in some tissue or cancer types and show a very large range of variation across samples within a cohort. We showed that this leads to artefactually negative correlations between mtOXPHOS and nuOXPHOS expression across samples within a cohort when using TPM or FPKM normalisations. Importantly, we also show that the effect of mtRNA expression variation between tissue samples leads to artefactual positive correlations between TPM values for random nuclear-encoded genes unrelated to the mitochondria. These artefacts are more severe for tissues or cancers with strong library composition biases, of which the most common is high levels of expression from mitochondrial DNA.

Because it allows direct comparison across samples, TPM is often considered to be a superior normalisation to FPKM [12, 13]. However, as the total sum of TPM values is invariant, the strength of the artefactual correlations introduced are actually greater than those observed with FPKM normalisation.

It has previously been pointed out that library composition biases make comparison of samples from different tissues problematic when using TPM or FPKM normalisation [13, 22]. Here we have demonstrated that library composition biases make comparisons and correlations of TPM/FPKM values problematic even when the analysis is limited to samples of the same tissue type.

These results have strong implications for any reported correlation analyses using expression values normalized by TPM or FPKM. As correlation coefficients between almost all pairs of nuclear genes are artificially inflated in almost every tissue, these artefacts can obscure the true relationship between genes, with negatively correlated gene pairs appearing to be strongly co-expressed. This also presents difficulties in understanding changes in the interactions of pairs of genes across tissues, as changes may be simply due to different library composition biases in those tissues. Lastly, this adds additional difficulties in interpretation of the already problematic [33] but frequently observed practice of plotting TPM values of a favourite gene for each tissue from the GTEx database side-by-side as a qualitative measure of tissue expression level, given that a gene’s mean TPM value for a tissue will in large part be determined by the mean mtRNA level of that tissue.

We demonstrate that scaling libraries using MRN or TMM algorithms is a simple and effective way to account for these biases prior to correlation analysis. We strongly dissuade researchers from using TPM or FPKM for gene expression correlation analysis and recommend that researchers employ alternative scaling normalisations that account for library composition bias before embarking on correlation analyses.

### Coordination between mitochondrial and nuclear gene expression

Our analysis using correct normalisation substantially updates our view of how gene expression is coordinated between mitochondrial and nuclear genomes. The lack of strong correlations between mtOXPHOS and nuOXPHOS gene expression in the majority of healthy tissues in the GTEx database implies that these genes are generally not strongly co-ordinated by retrograde signalling pathways. The broad coordination afforded by tissue-specific transcriptional programs [2] is likely sufficient for supporting tissue function under normal physiological conditions. However, we found that the tissue expression level of NF-κB genes is associated with the strength of coordination between mtOXPHOS and nuOXPHOS expression, supporting a role for this complex in mito-nuclear communication as proposed previously [31]. The biological significance of strong negative correlations, such as observed in the left ventricle of the heart and the liver, remains unclear. However, we note that they tend to be observed in tissues with very high expression levels from the mitochondrial genome.

It is clear from our analysis that the mechanisms by which apparent coordination between the mitochondrial and nuclear OXPHOS genes arises differ sharply between healthy tissues and tumour cells. The positive correlation of mtOXPHOS and nuOXPHOS genes across samples within many cancer types does not necessarily imply that retrograde signalling is active in cancers, as other differences between healthy tissues and tumours could play a role. Both mtOXPHOS and nuOXPHOS gene expression are often altered in cancers [34]. Positive correlations might result from selection within tumours for clones with concordant OXPHOS expression, with this selection becoming stronger with higher mtOXPHOS expression; this might explain the strong positive relationship between mtOXPHOS-nuOXPHOS correlation and mtRNA levels among TCGA cancer types.

Intriguingly, however, we found that although cancers tend to exhibit positive mtOXPHOS-nuOXPHOS correlation, the strength of this correlation in a particular cancer type relates strongly to its inferred proliferation status, with stronger proliferation related to lower mtOXPHOS-nuOXPHOS correlation. This may be due to the Warburg effect, in which metabolism in rapidly proliferating tumour cells is reported to shift towards aerobic glycolysis, thereby bypassing the mitochondrial role in respiration [35]. In the fastest proliferating cancer types, the importance of mitochondrial respiration may therefore be reduced, leading to reduced selection within tumours for concordant OXPHOS expression. Thus, although selection between cells within proliferating tumours may act to produce a positive mtOXPHOS-nuOXPHOS correlation in most cancers, this selection may be weakened and increasingly dominated by the Warburg effect in the fastest proliferating cancers.

## Conclusions

Understanding how coordination between mitochondrial and nuclear genes with mitochondrial function is an important question in biology. Large-scale datasets showing gene expression across humans, either in healthy tissues (GTEx) or cancer (TCGA) represent attractive options to analyse this question. Here we provided a robust methodology to normalize these data correctly so that correlations in gene expression can be assessed. On the basis of this, we showed that the correlation between mitochondrial and nuclear gene expression was weak in most tissues. Tissues with higher correlations are associated with increased NF-κB subunit expression, implicating this pathway in nuclear-mitochondrial communication. Contrastingly, we demonstrated that cancers have much stronger coordination between mitochondrial and nuclear gene expression, but that the fastest proliferating cancers have a weaker correlation. These data highlight the complex, context-specific control of mitochondrial and nuclear gene expression coordination and its sensitivity to both external and intrinsic factors.

## Methods

### Raw data

RNA-Seq data were downloaded from the GTEx data portal for GTEx V8, apart from data shown in Additional File 1: Fig S2 from GTEx V6p. Data were downloaded as normalized TPM values (or FPKM values for GTEx V6p, later also converted to TPM) or as raw counts. ‘Harmonised’ (hg38) RNA-seq data were downloaded for TCGA projects using the ‘*TCGAbiolinks*’ package in *R* as normalized FPKM values or as raw counts.

For TCGA cancer type analyses, we only considered samples annotated as Primary Tumours, except where we explicitly note that we perform analyses on adjacent normal tissue samples.

We were mindful of the possibility of contamination of mitochondrial RNA reads by the expression of nuclear integrations of mitochondrial DNA (NUMTs). Previous studies have established that NUMT contamination of mtDNA expression quantification in RNAseq data is negligible for the GTEx [8] and TCGA [34] databases.

We restricted our analyses to GTEx tissues with at least 100 samples or TCGA cancer types with at least 50 Primary Tumour samples.

### mtOXPHOS and nuOXPHOS genes

The list of mtOXPHOS and nuOXPHOS genes was taken from [8] and can be found in Additional File 10.

### Normalisations

For TCGA data and GTEx V6p, TPM values were obtained by converting FPKM values according to the following formula for gene *i*:

$$ {\mathrm{TPM}}_i=\frac{{\mathrm{RPKM}}_i}{\mathrm{Total}\ \mathrm{library}\ \mathrm{RPKM}}\times {10}^6 $$

The MRN normalisation was performed using the ‘*DESeq2*’ package in *R*. Normalisations were applied both individually for each tissue or cancer type cohort and across all samples within each database. Normalized pseudocounts were obtained by converting raw counts data to a *DESeq*DataSet using the ‘DESeqDataSetFromMatrix()’ function, applying the ‘estimateSizeFactors()’ function to the resulting dds object, and then retrieving the normalized pseudocounts with the function ‘counts()’ with ‘normalized = TRUE’.

The TMM and UQ normalisations were performed using the ‘*edgeR*’ package in *R*. Normalisations were applied individually for each tissue or cancer type cohort. Raw counts were converted into a DGEList object with the function ‘DGEList’. Normalisation factors were returned using the function ‘calcNormFactors()’ with method set as either ‘TMM’ or ‘UQ’. Library scaling factors were then obtained by multiplying the resulting library sizes by the resulting normalisation factors. All read counts for each library/sample were then multiplied by 10^6^ and divided by the scaling factor for that library.

TPM (nuclear only) values for nuclear-encoded genes were obtained by multiplying raw read counts for nuclear genes by 10^6^ and dividing by the sample sum of reads for all genes excluding those borne on mitochondrial DNA. mtDNA-encoded genes were normalized with the total sample sum instead, as for TPM. We did not normalize for transcript length; the values thus calculated should be considered as counts per million. However, for correlation analysis, which does not involve direct comparison of genes within samples, this is equivalent to TPM.

TPM values excluding haemoglobin reads or *PRSS1/2* reads for GTEx Whole Blood or Pancreas samples, respectively, were obtained as for TPM nuclear only but excluding the read counts for those genes when computing the total sample read count, instead of the read counts for the mitochondrial genes.

### Correlations

Correlations were computed using the ‘cor.test()’ function in *R*, with ‘method = “spearman”’ or ‘method = “pearson”’.

### Linear model correction of data prior to correlation analysis

Before correlation analysis, we applied linear model corrections to normalized data to control for known confounders in the GTEx and TCGA datasets.

Sample and donor information for the GTEx were downloaded from the GTEx data portal in the form of the Subject Phenotypes and Subject Annotations files accompanying GTEx V8 (or V6p as appropriate). TCGA metadata was obtained using the ‘all_metadata(subset = “tcga”)’ function of the ‘*recount*’ package in *R*. Some TCGA donors provided multiple cancer samples; these were all excluded to leave only samples unique to a single donor.

For single-tissue or cancer type analyses, linear models within each tissue or cancer type cohort were applied, to account for tissue-specific trends. TMM and MRN normalisations were applied within the tissue or cancer type cohort. A linear model was fitted for each gene using the ‘lm()’ function in *R*, and the resulting residuals were used downstream in correlation analyses. GTEx data were corrected for the age bracket of the patient, sex of the patient, cause of death (Hardy scale), sample ischemic time and sequencing batch. The age bracket was considered to be a numerical variable, and the midpoint of the appropriate age bracket was taken for each sample to be entered into the linear model. We opted to use the publicly available age-bracket data so that our analyses can be readily reproduced. TCGA data (including matched normal samples) was corrected for the gender, race, tumour stage and age of the patient and the sequencing centre where the samples were processed.

For single-tissue GTEx analyses accounting for cell-type composition estimates and genotyping principal components, we downloaded the cell type composition estimates for available tissues from the supplementary material of [26] (see Availability of Data and Materials section). We downloaded genotyping principal components from the eQTL covariates file from the GTEx download portal. We then included these factors in linear models for each gene in addition to the five factors reported above and proceeded with our analysis using the residuals from these models.

For single cancer-type TCGA analyses accounting for cell-type composition-based subtypes, we downloaded the ‘*DeClust*’ *R* package [27] from GitHub [36], which loads pre-calculated DeClust subtypes for 13 TCGA cancer types. We then included the sample DeClust subtype in the linear models for each gene alongside the other potentially confounding variables and proceeded with our analysis using the residuals from these models.

For analyses combining different GTEx tissues, we accounted for the fact that multiple samples from different tissues may derive from the same donor. TMM and MRN normalisation were applied across all tissues. We then applied a linear mixed-effects model for each gene across all samples from all tissues, with age bracket, sex, cause of death and sample ischemic time interacting with tissue type. Additionally individual donor and sequencing batch were included as random effects. We then proceeded with residuals from these mixed-effects models. As sample independence was not an issue for the TCGA data, we used residuals from the single-cancer type analysis in the combined analysis.

### Correlations combining tissues or cancer types

To perform a correlation analysis on combined tissues or cancer types, we took the residuals of expression data that had been normalized and corrected by fitting a linear model to control for confounding variables, as above. We filtered the tissue or cancer types for those with at least 100 (GTEx) or 50 (TCGA) samples, leaving 48 tissues and 31 cancers, respectively. To ensure equal representation of each tissue or cancer type, we randomly sampled 100 (GTEx) or 50 (TCGA) from each tissue. Combining raw gene expression residuals for tissues or cancer types with different mtRNA levels may introduce artefacts, as high-expressing tissues will have much greater variance in the absolute value of the residuals, despite the linear model controlling for the tissue average gene expression level. To account for this, we ranked the sample residuals for each gene within the sample chosen for each tissue or cancer type. We then combined the ranks for the chosen samples across tissue types, using the ranks in place of the raw residuals; this gave us 4800 samples for GTEx or 1550 samples for TCGA. The Spearman’s correlation was then computed across these aggregated ranks. As the result varies slightly depending on the random sampling within each tissue, we repeated this process 100 times. Heatmaps show the median correlation for any gene pair over the 100 iterations. The order of mtOXPHOS/nuOXPHOS genes for all heatmaps for both GTEx and TCGA data throughout the manuscript is identical and was determined by clustering of the correlations for the MRN-normalized data combining GTEx tissues. Although the gene names are not shown in main figure panels, the genes are fully annotated with HGNC symbols in Additional Files 2-5.

In order to provide a null distribution for the correlation of mtOXPHOS genes with nuOXPHOS genes, we repeated the process as above, but replaced the 126 nuOXPHOS genes with a random sample of 126 expressed nuclear genes (median TPM > 5 across all samples for all tissues/cancer types) for each iteration. This process was repeated 100 times. In order to test the observed mtOXPHOS-nuOXPHOS correlation against this distribution, the 100 median correlations for mtOXPHOS-nuOXPHOS gene pairs for each iteration was compared to the 100 median correlations of mtOXPHOS genes with random nuclear genes with a two-tailed *t* test.

### Correlations within tissues or cancer types

For correlations reported within cancer types or tissue types, all available samples were utilised for the calculations. To provide a bootstrapped distribution, we calculated the median mtOXPHOS-nuOXPHOS for each tissue or cancer type 1000 times in samples constituted by resampling with replacement. The 95% confidence intervals shown in the main figure panels were the observed 2.5th and 97.5th percentiles in the bootstrap distribution. We used empirical *p* values from the bootstrap distributions to test if observed median mtOXPHOS-nuOXPHOS correlations were different from 0. Raw *p* values were the fraction of bootstrap values > 0 for tissue/cancer types *s* with observed negative correlations or < 0 for positive correlations. These *p* values were then corrected to FDR across tissue/cancer types. Prior to correction, if any bootstrap distribution did not contain 0 or opposite sign correlations, the observed 0 was substituted for 1/1000 and the *p* value was reported to be less than this value after FDR adjustment.

To test the significance of mtOXPHOS-nuOXPHOS correlations against mtOXPHOS with random nuclear genes, correlations were computed for each tissue/cancer type for the mtOXPHOS genes and 126 random nuclear genes (median TPM > 5 across all samples from all tissues/cancer types). This was repeated 100 times and the medians of the correlations of mtOXPHOS genes with random nuclear genes from each iteration were taken to be the null distribution. We confirmed that these medians conformed to a normal distribution, as would be expected under the Central Limit Theorem, by performing a Shapiro-Wilk normality test on the 100 medians for each tissue. The adjusted *p* values (FDR) for the Shapiro-Wilk tests are reported in Additional File 1: Table S1 & S2. The *Z* statistic for the observed median mtOXPHOS-nuOXPHOS correlation was then calculated by subtracting the mean and dividing by the standard deviation of the mtOXPHOS-random nuclear medians. *p* values were then calculated for each tissue according to the normal cumulative distribution function and corrected to FDR (Additional File 1: Table S1 & S2).

### Matched cancer and normal samples

Matched primary tumour and adjacent normal tissue samples were identified using TCGA metadata and barcodes. Tissues were identified with at least 10 normal samples. Using the donor portion of the TCGA barcode, matching primary tumour samples were identified. If multiple primary tumour samples matched the adjacent normal tissue sample, one was retained at random and the remainder were discarded. Additionally, normal tissue samples without identifiable primary tumour samples in the expression data were discarded, such that all normal tissue samples had one matching primary tumour sample and vice versa. mtOXPHOS-nuOXPHOS correlations were computed using all samples. To calculate the bootstrapped standard error, we repeated the correlations using unique subsets of 90% of the datapoints 100 times. The bootstrapped standard error for the median mtOXPHOS-nuOXPHOS correlation is the standard deviation of the bootstrapped median mtOXPHOS-nuOXPHOS correlations.

### Dip test for bimodality

To test for bimodality in the distribution of correlations among random nuclear genes, correlations for 100 sets of random genes were computed for all GTEx tissues using MRN-normalized data. Hartigan’s dip test was then performed for each set of 100 random genes in each tissue using the ‘dip.test()’ function of the ‘*diptest*’ package in *R*. Dip test *p* values were adjusted to FDR within each tissue.

### Gene ontology enrichment analysis

To test for genes whose expression was associated with mtOXPHOS-nuOXPHOS coordination, we calculated the mean expression of each gene in each tissue or cancer type (in pseudocounts generated by MRN normalisation of raw counts across all tissues/cancers). We then computed the Spearman’s correlation for each gene of the mean tissue/cancer pseudocounts with tissue/cancer mtOXPHOS-nuOXPHOS correlation calculated earlier using MRN normalisation. We ordered the gene lists in order of correlation and took the top or bottom 1000 genes. As these were ENSEMBL gene IDs, we retrieved the HGNC symbols for these genes using the ‘*biomaRt*’ package in *R*; this typically resulted in the loss of a fraction of the genes which lack HGNC symbols (such as some lncRNA genes). We then submitted the HGNC symbols to the ‘enrichr()’ function of the ‘*enrichR*’ package in *R* [37] using the reference databases ‘KEGG_2021_Human’, ‘GO_Molecular_Function_2018’, ‘GO_Cellular_Component_2018’ and ‘GO_Biological_Process_2018’. *p* values computed by ‘*enrichR*’ which we used to assess the significance of enrichments are calculated using Fisher’s exact test.

In order to be sure our enrichment results were specific to mtOXPHOS-nuOXPHOS correlation, we repeated the above process but using the top or bottom 1000 genes which correlated with median mtOXPHOS-random nuclear correlation in each tissue.

### Proliferative Index

The Proliferative Index (PI) was calculated with the ‘*ProliferativeIndex*’ package in *R*. Briefly, the entire dataset across all tissues or cancer types was normalized by MRN and variance stabilising transformation using the ‘varianceStabilizingTransformation()’ function of *DESeq2*. Following the normalisation, the PI was calculated by applying the ‘readDataForPI()’ function with a randomly selected gene specified in the ‘modelIDs’ argument, then running ‘calculatePI()’ on the resulting object.

### Estimations of total immune fraction

The estimates for the total immune fraction of GTEx samples were taken directly from the GitHub repository associated with the GEDIT tool [28, 38].

## Supplementary Information

**Additional File 1: Supplementary figures and tables. Contains Fig S1-S11 and Table S1-S5.**

**Additional File 2.** GTEx mtOXPHOS-nuOXPHOS heatmaps. mtOXPHOS-nuOXPHOS expression heatmaps using MRN normalization for 48 GTEx tissues.

**Additional File 3.** TCGA mtOXPHOS-nuOXPHOS heatmaps. mtOXPHOS-nuOXPHOS expression heatmaps using MRN normalization for 31 TCGA cancer types.

**Additional File 4.** TCGA matched tumour heatmaps. mtOXPHOS-nuOXPHOS expression heatmaps for matched tumour samples for 14 cancer types.

**Additional File 5.** TCGA matched normal heatmaps. mtOXPHOS-nuOXPHOS expression heatmaps for matched normal samples for 14 cancer types.

**Additional File 6.** GTEx top 1000 Gene Ontology. Gene Ontology enrichment for the top 1000 genes, whose expression across GTEx tissues is most positively correlated with tissue mtOXPHOS-nuOXPHOS correlation. Note the spreadsheet has multiple tabs corresponding to different gene set libraries interrogated.

**Additional File 7.** GTEx bottom 1000 Gene Ontology. Gene Ontology enrichment for the bottom 1000 genes, whose expression across GTEx tissues is most negatively correlated with tissue mtOXPHOS-nuOXPHOS correlation. Note the spreadsheet has multiple tabs corresponding to different gene set libraries interrogated.

**Additional File 8.** TCGA top 1000 Gene Ontology. Gene Ontology enrichment for the top 1000 genes whose expression across TCGA cancer types is most positively correlated with cancer type mtOXPHOS-nuOXPHOS correlation. Note the spreadsheet has multiple tabs corresponding to different gene set libraries interrogated.

**Additional File 9.** TCGA bottom 1000 Gene Ontology. Gene Ontology enrichment for the bottom 1000 genes, whose expression across TCGA cancer types is most negatively correlated with cancer type mtOXPHOS-nuOXPHOS correlation. Note the spreadsheet has multiple tabs corresponding to different gene set libraries interrogated.

**Additional File 10.** OXPHOS gene names and IDs. The spreadsheet containing HGNC symbols and ENSEMBL gene IDs for mtOXPHOS and nuOXPHOS genes used in this study.

## Data Availability

The datasets supporting the conclusions of this article are available in the dbGaP repository: GTEx V8 data (accession phs000424.v8.p2), GTEx V6p (accession phs000424.v6.p1) and TCGA (accession phs000178.v11.p8). GTEx sample cell type composition estimates were obtained from ref. [26], Supplementary Datasets 4-9 and 11-17. TCGA deClust deconvolution subtypes were obtained from the ‘*DeClust*’ *R* package [27, 36]. GTEx immune cell fraction predictions were obtained from refs [28, 38]. All code used to produce the analyses presented in the manuscript is available on Github [39] under a GPL 3.0 licence and on Zenodo [40] under a Creative Commons licence 4.0.
