# Supplementary material for: Malignancy and NF-κB signalling strengthen coordination between expression of mitochondrial and nuclear-encoded oxidative phosphorylation genes
Source: Genome Biol. 2021 Dec 2;22:328. doi: 10.1186/s13059-021-02541-6 (PMC8638269; doi:10.1186/s13059-021-02541-6)
Supplement: Supplementary file 1 — Additional File 1: Supplementary figures and tables. Contains Fig S1-S11 and Table S1-S5. [file 13059_2021_2541_MOESM1_ESM.docx]

**
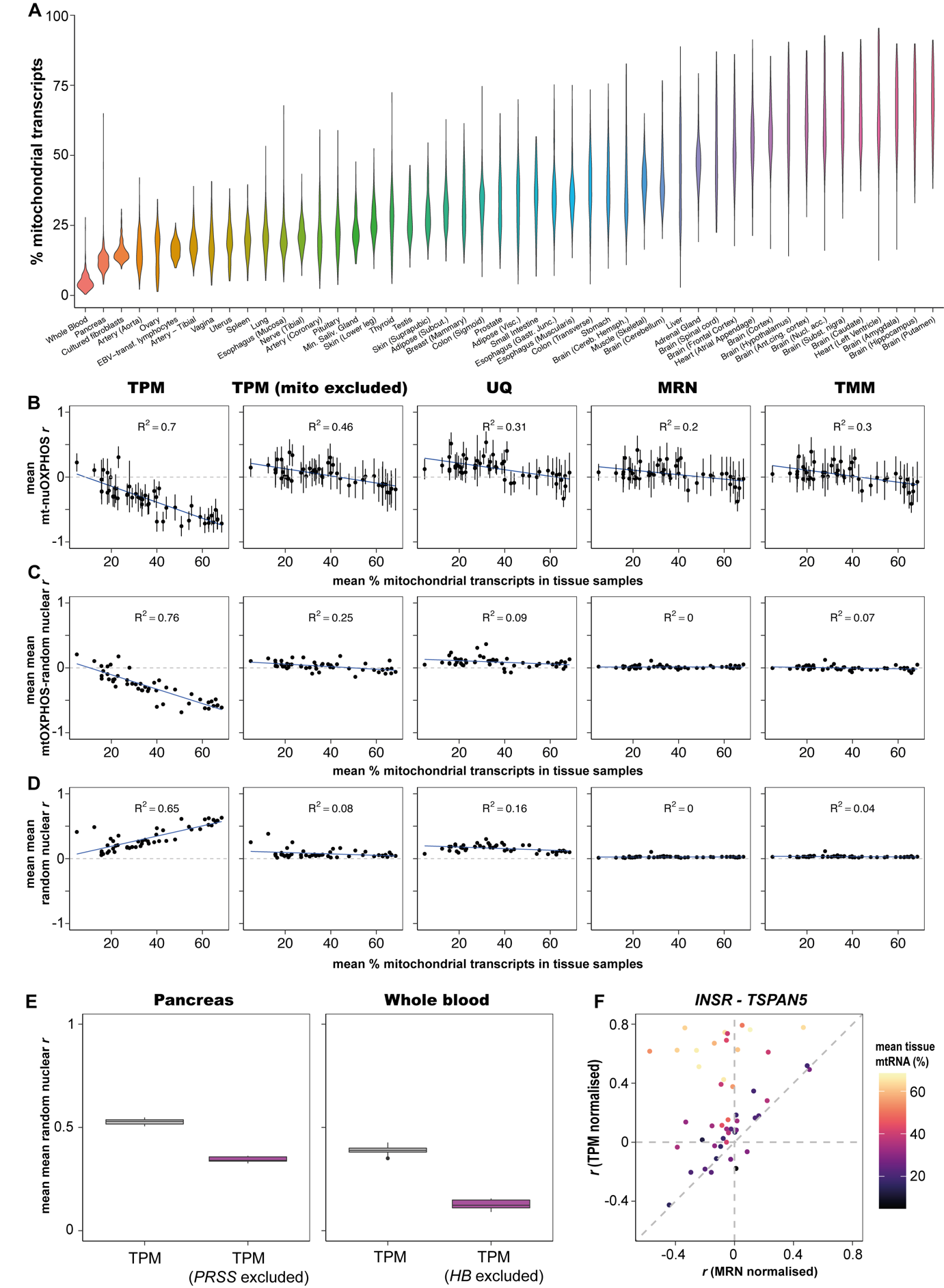
**

**Fig S1. Mitochondrial library bias drive artefactual correlations using Pearson’s correlation.**

**A)** Violin plot showing total expression from mtDNA-encoded genes in samples from all 48 healthy human tissues with more than 100 samples from the GTEx database.

**B)** Scatterplot shows mean Pearson’s correlation (*r*) of mtOXPHOS-nuOXPHOS gene pairs within 48 GTEx tissues vs tissue mean of mtRNA expression as a percentage of total transcripts. Shown for data normalized by TPM, TPM excluding mitochondrial reads for normalizing nuclear genes, UQ, MRN and TMM methods. Error bars indicate SD. Blue line shows linear regression with R^2^ noted within panel.

**C)** Scatterplot shows mean over 100 samples of the tissue mean *r* of mtOXPHOS with 126 random expressed nuclear genes vs tissue mean of mtRNA expression as a percentage of total transcripts. Shown for data normalized by TPM, TPM excluding mitochondrial reads for normalizing nuclear genes, UQ, MRN and TMM methods. 95% confidence interval error bars are smaller than the plotted symbols. Blue line shows linear regression with R^2^ noted within panel. Green circles for MRN and TMM highlight unusually high correlations in the testis.

**D)** Scatterplot shows mean over 100 samples of the tissue mean *r* within 100 random expressed nuclear genes within tissues vs tissue mean of mtRNA expression as a percentage of total transcripts. Shown for data normalized by TPM, TPM excluding mitochondrial reads for normalizing nuclear genes, UQ, MRN and TMM methods. 95% confidence interval error bars are smaller than the plotted symbols. Blue line shows linear regression with R^2^ noted within panel.

**E)** Boxplot shows mean values for 10 samples of the mean *r* within 100 random expressed nuclear genes with TPM normalization or TPM excluding the read counts for *PRSS1* & *PRSS2* (pancreas) or *HBA1*, *HBA2*, *HBB* and *HBD* (whole blood).

**F)** Scatterplot shows Pearson’s *r* within tissues for two genes, *INSR* and *TSPAN5,* for TPM normalized data and MRN normalized data. Colour indicates the tissue mean mtRNA expression (% total transcripts).


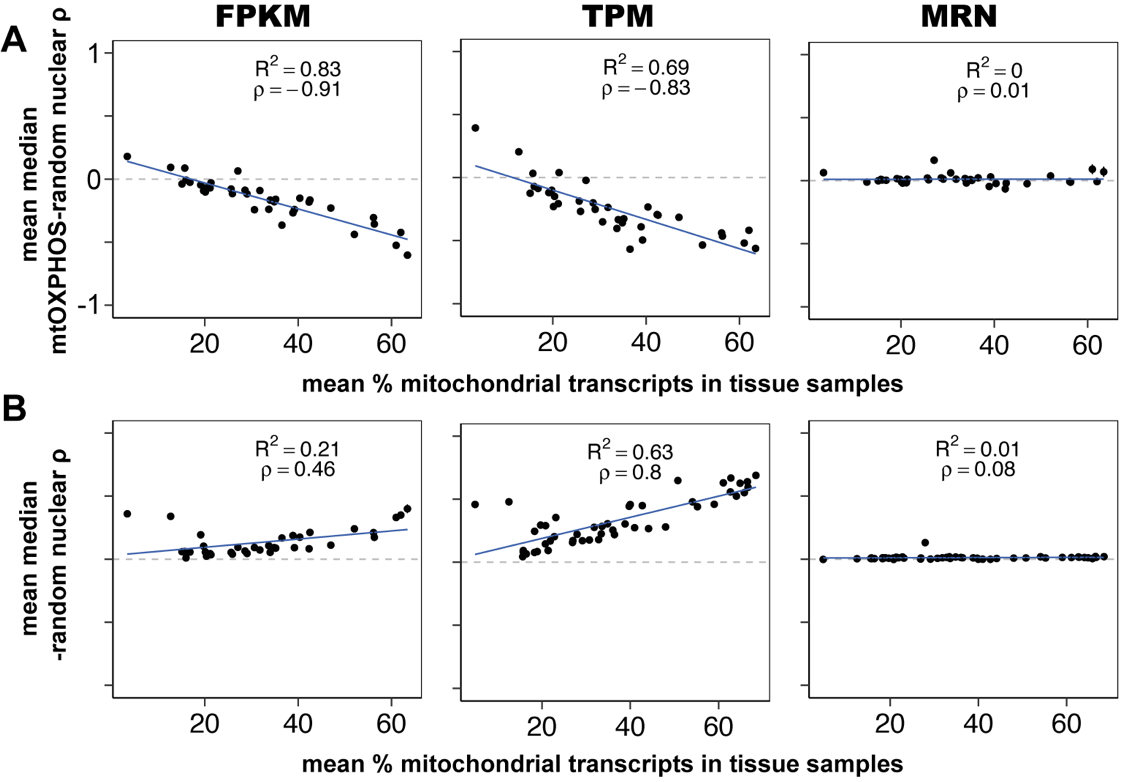


**Fig S2.** **Mitochondrial library bias drive artefactual correlations using FPKM in addition to TPM.**

**A)** Scatterplot shows mean over 100 samples of the tissue median Spearman’s ρ of mtOXPHOS with 126 random expressed nuclear genes vs tissue mean of mtRNA expression as a percentage of total transcripts. Shown for data normalized by FPKM, TPM, or MRN. 95% confidence interval error bars are smaller than the plotted symbols. Blue line shows linear regression with R^2^ and Spearman’s ρ noted within panel.

**B)** Scatterplot shows mean over 100 samples of the tissue median ρ within 100 random expressed nuclear genes vs tissue mean of mtRNA expression as a percentage of total transcripts. Shown for data normalized by TPM, TPM excluding mitochondrial reads for normalizing nuclear genes, UQ, MRN and TMM methods. 95% confidence interval error bars are smaller than the plotted symbols. Blue line shows linear regression with R^2^ noted within panel.

Data in this figure are drawn from GTEx V6p, rather than GTEx V8 as for all other figures.


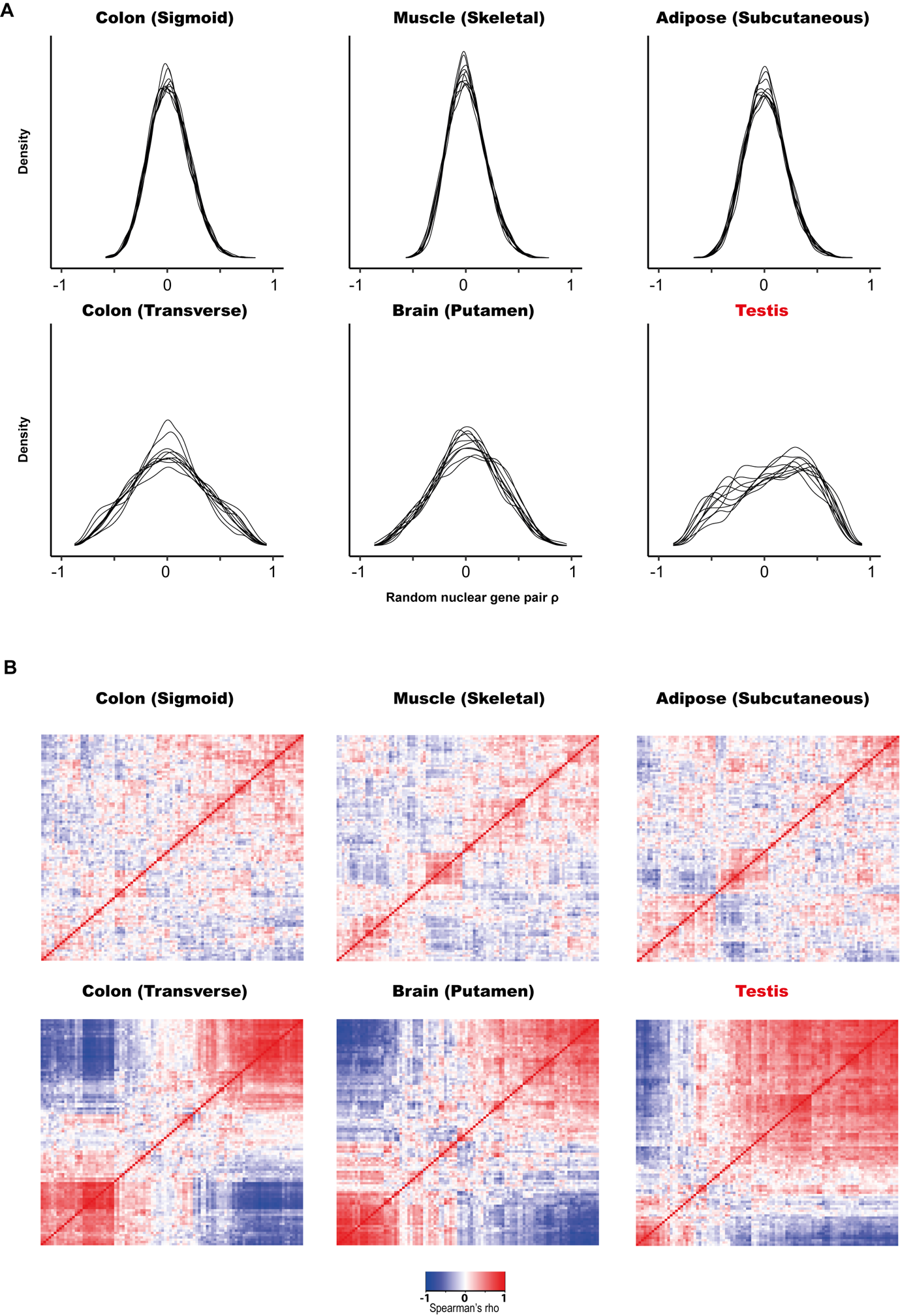


**Fig S3. Correlation distributions vary consistently across tissues, with the testis unique in displaying signs of bimodal correlation distribution.**

**A)** Density of correlation coefficients within 10 random samples (overlaid lines) of 100 random nuclear genes for GTEx tissues with narrow distributions (above) and wide distributions (below). The testis shows clear signs of bimodality. Correlations performed on MRN-normalized data.

**B)** Representative heatmaps for Spearman’s correlations of 100 random genes for the same tissues.


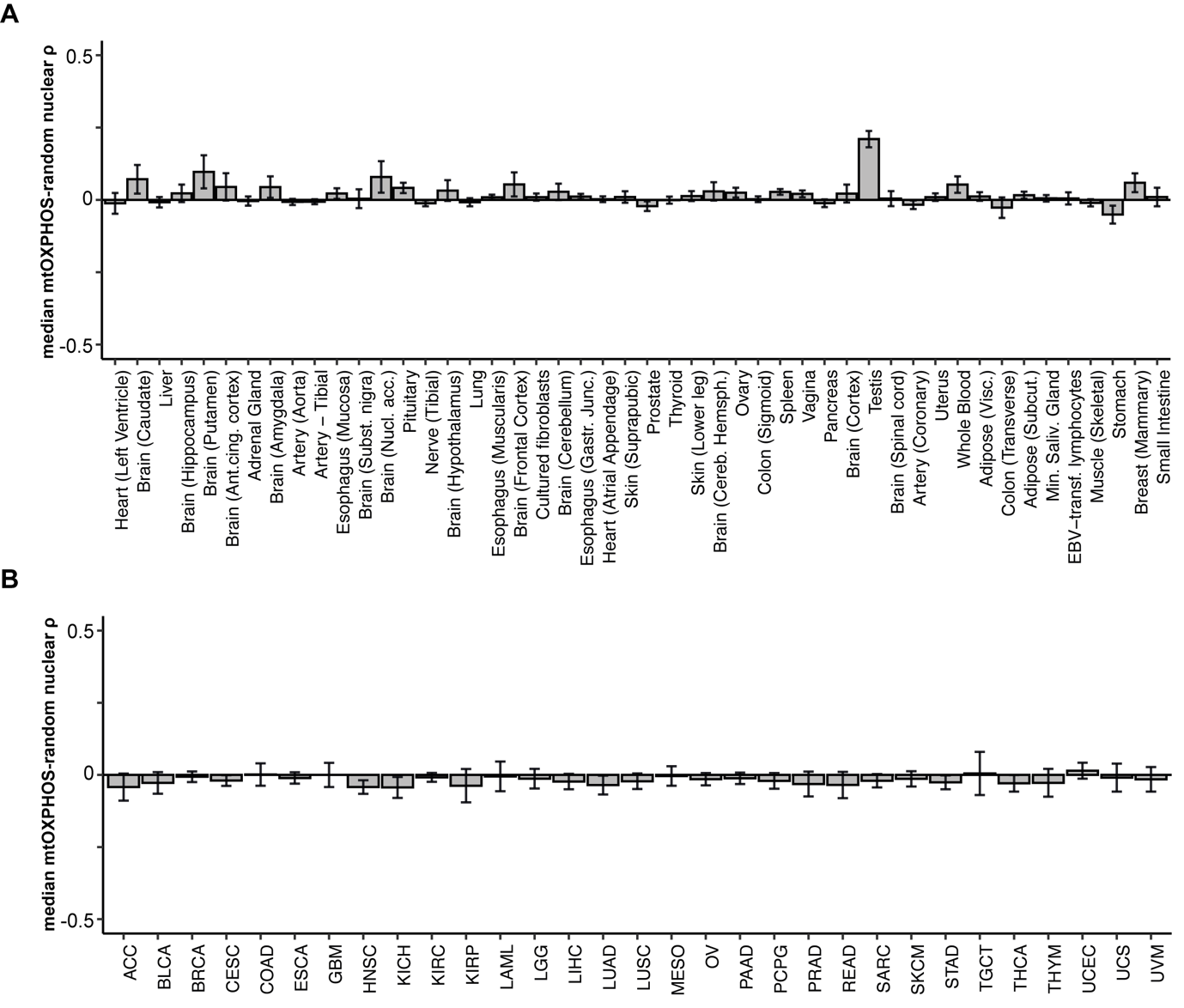


**Fig S4. Correlations of mtOXPHOS with random nuclear genes do not resemble the observed mtOXPHOS-nuOXPHOS correlations across tissues or cancer types.**

**A)** Bars show mean median ρ for mtOXPHOS genes with 100 samples of 126 random nuclear genes in GTEx tissues; error bars indicate standard error. The testis has unusually high correlations between mtOXPHOS and random genes. The order of tissues matches the order of mtOXPHOS-nuOXPHOS correlation as shown in Fig 2D. The observed mtOXPHOS-nuOXPHOS correlation for each tissue is tested against this distribution in Table S1.

**B)** Bars show mean median ρ for mtOXPHOS genes with 100 samples of 126 random nuclear genes in TCGA cancer types; error bars indicate standard error. The order of cancer types matches the order of mtOXPHOS-nuOXPHOS correlation as shown in Fig 4C. The observed mtOXPHOS-nuOXPHOS correlation for each cancer type is tested against this distribution in Table S2. Cancer abbreviations are found in Table S5.


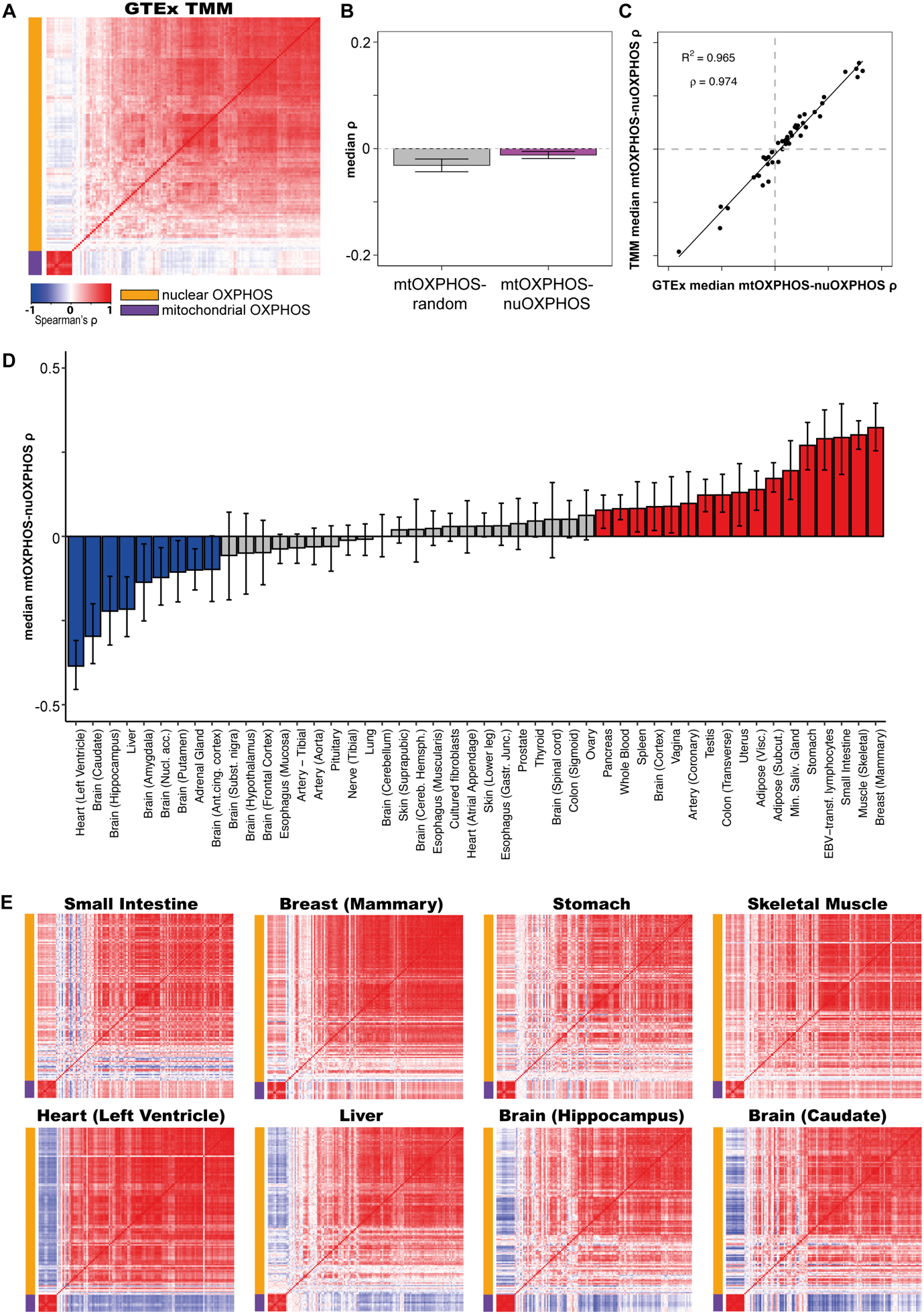


**Fig S5. TMM normalization supports weak and inconsistent correlations between mtOXPHOS and nuOXPHOS within tissues.**

**A)** Heatmap showing median Spearman’s ρ for mtOXPHOS and nuOXPHOS gene expression using TMM normalization in 48 GTEx tissues combined (100 samples from each tissue, sampled 100 times).

**B)** Boxplot showing median Spearman’s ρ for 100 iterations of mtOXPHOS genes with random nuclear genes or mtOXPHOS with nuOXPHOS genes for 48 GTEx tissues combined.

**C)** Scatterplot showing tissue median mtOXPHOS-nuOXPHOS ρ from analysis with MRN normalisation or TMM normalisation.

**D)** Observed median Spearman’s ρ between mtOXPHOS and nuOXPHOS genes for 48 GTEx tissues. Error bars show 95% bootstrap confidence interval. Blue bars indicate observed correlation significantly lower than 0 (bootstrap empirical p-value, FDR-adjusted < 0.05), red bars indicate observed correlation significantly higher than 0 and grey bars indicate FDR > 0.05.

**E)** Heatmap showing Spearman’s ρ for mtOXPHOS and nuOXPHOS genes for 8 GTEx tissues showing clear positive or negative correlations.

**
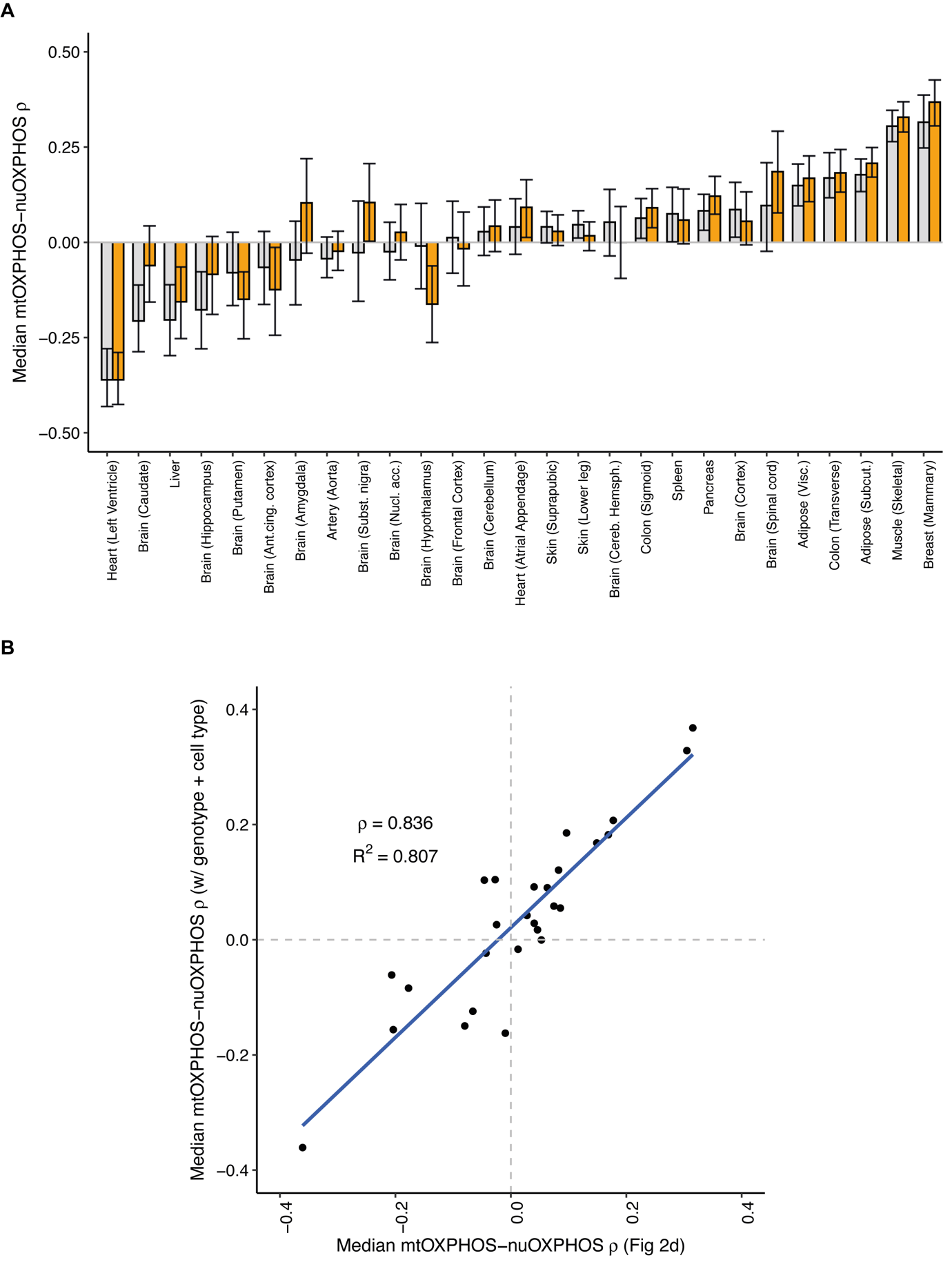
**

**Fig S6. Cell type composition and genetic ancestry are not major confounders of mtOXPHOS-nuOXPHOS correlation in the GTEx database.**

**A)** Bar chart showing median mtOXPHOS-nuOXPHOS Spearman’s ρ for 27 GTEx tissues with available estimates of sample cell type composition, taken from ref: 26. Orange bars indicate the median mtOXPHOS-nuOXPHOS correlation of residuals after additionally correcting for sample cell type composition and genetic ancestry (the top 5 genotyping principal components). Grey bars indicate the tissue estimate without additional correction (identical to tissue estimates in Fig 2D). Error bars indicate 95% bootstrap confidence intervals.

**B)** Scatterplot showing median mtOXPHOS-nuOXPHOS Spearman’s ρ for 27 GTEx tissues with or without additional correction for sample cell type composition and genetic ancestry.


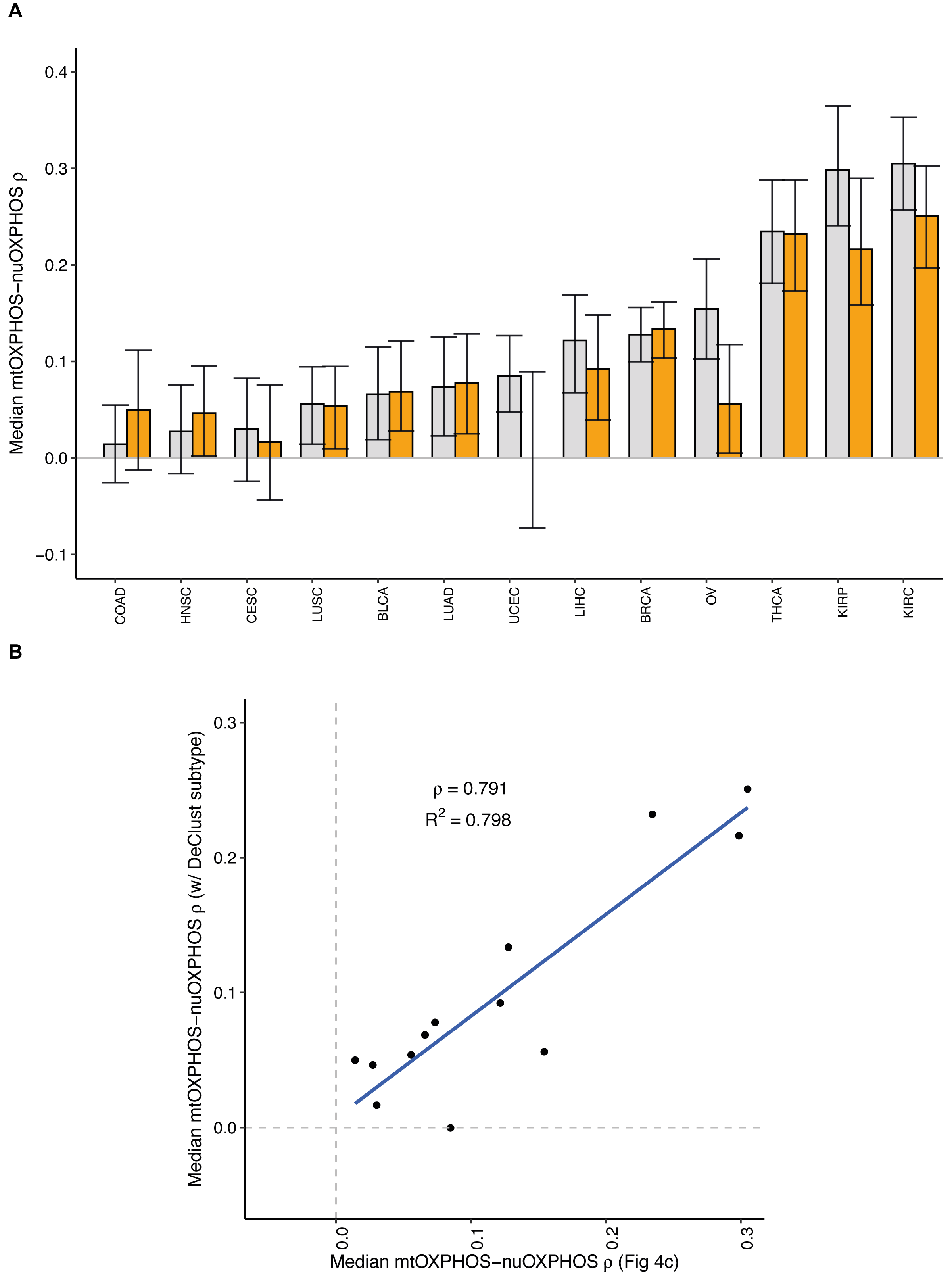
**Fig S7. Cell type composition is not a major confounder of mtOXPHOS-nuOXPHOS correlation in the TCGA database.**

**A)** Bar chart showing median mtOXPHOS-nuOXPHOS Spearman’s ρ for 13 TCGA cancer types with available sample molecular subtype based on estimates of sample cell type composition, taken from ref: 27. Orange bars indicate the median mtOXPHOS-nuOXPHOS correlation of residuals after additionally correcting for molecular subtype. Grey bars indicate the tissue estimate without additional correction (identical to cancer type estimates in Fig 4C). Error bars indicate 95% bootstrap confidence intervals.

**B)** Scatterplot showing median mtOXPHOS-nuOXPHOS Spearman’s ρ for 13 TCGA cancer types with or without additional correction for sample molecular subtype.

**
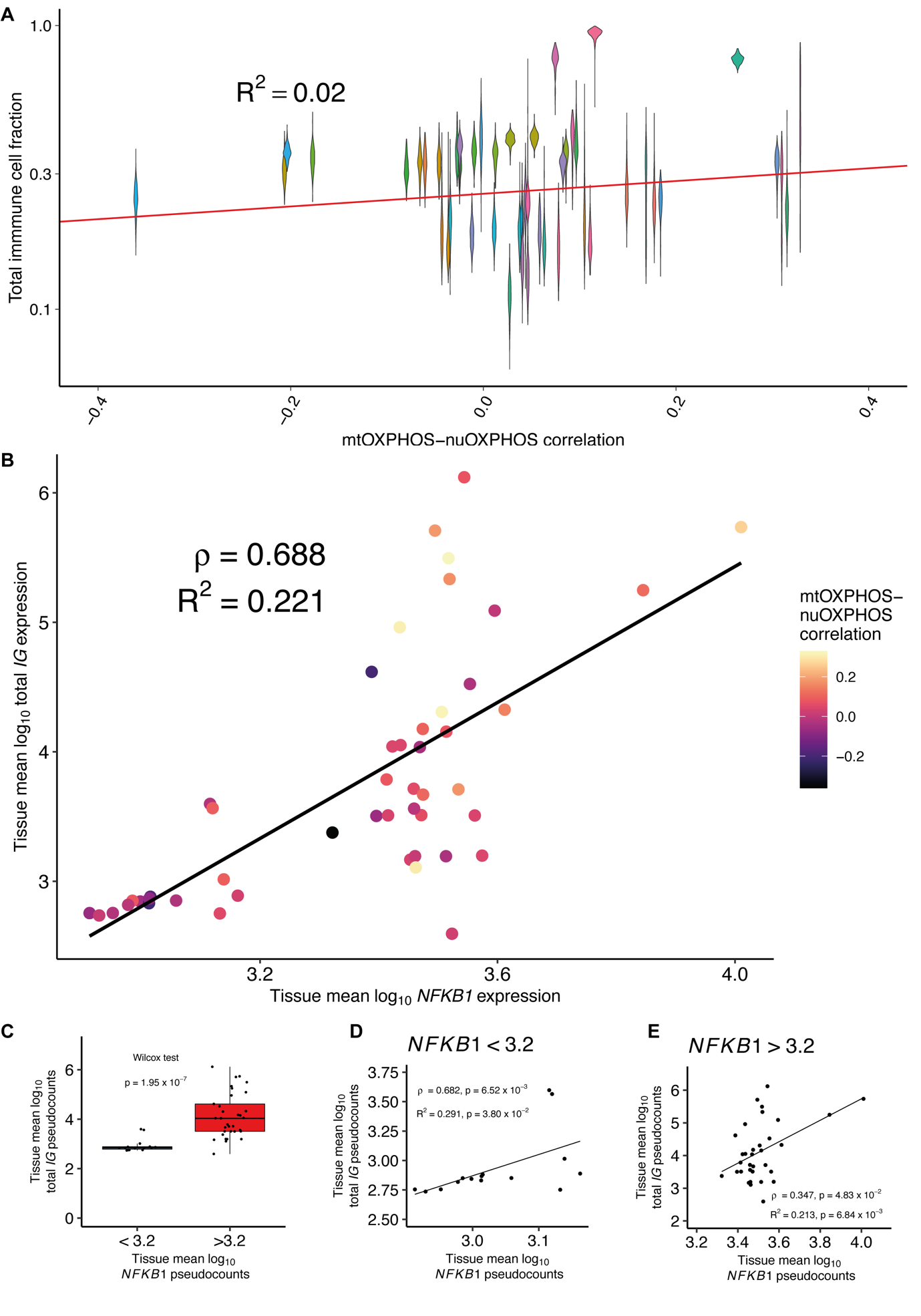
**

**Fig S8. Total inferred immune cell fraction relates poorly to mtOXPHOS-nuOXPHOS correlation.**

**A)** Violins represent tissue types. Total inferred immune cell fraction for each sample was inferred from GEDIT, a gene expression deconvolution tool.

**B)** Scatterplot showing tissue mean log_10_ *NFKB1* expression against total log_10_ gene expression from all immunoglobulin-encoding *IG* genes (pseudocounts from MRN normalisation). Plots are coloured according to tissue mtOXPHOS-nuOXPHOS correlation.

**C)** Despite not being significantly bimodal, there appears to be a discontinuity in the *NFKB1* distribution. Boxplot shows total log_10_ *IG* expression for tissues with mean log_10_ *NFKB1* expression above or below 3.2.

**D)** Scatterplot showing tissue mean log_10_ *NFKB1* expression against total log_10_ gene expression from all immunoglobulin-encoding *IG* genes for tissues with ‘low’ NFKB1 expression. R^2^ and Spearman’s ρ are shown with corresponding p-values.

**E)** Scatterplot showing tissue mean log_10_ *NFKB1* expression against total log_10_ gene expression from all immunoglobulin-encoding *IG* genes for tissues with ‘high’ NFKB1 expression. R^2^ and Spearman’s ρ are shown with corresponding p-values.


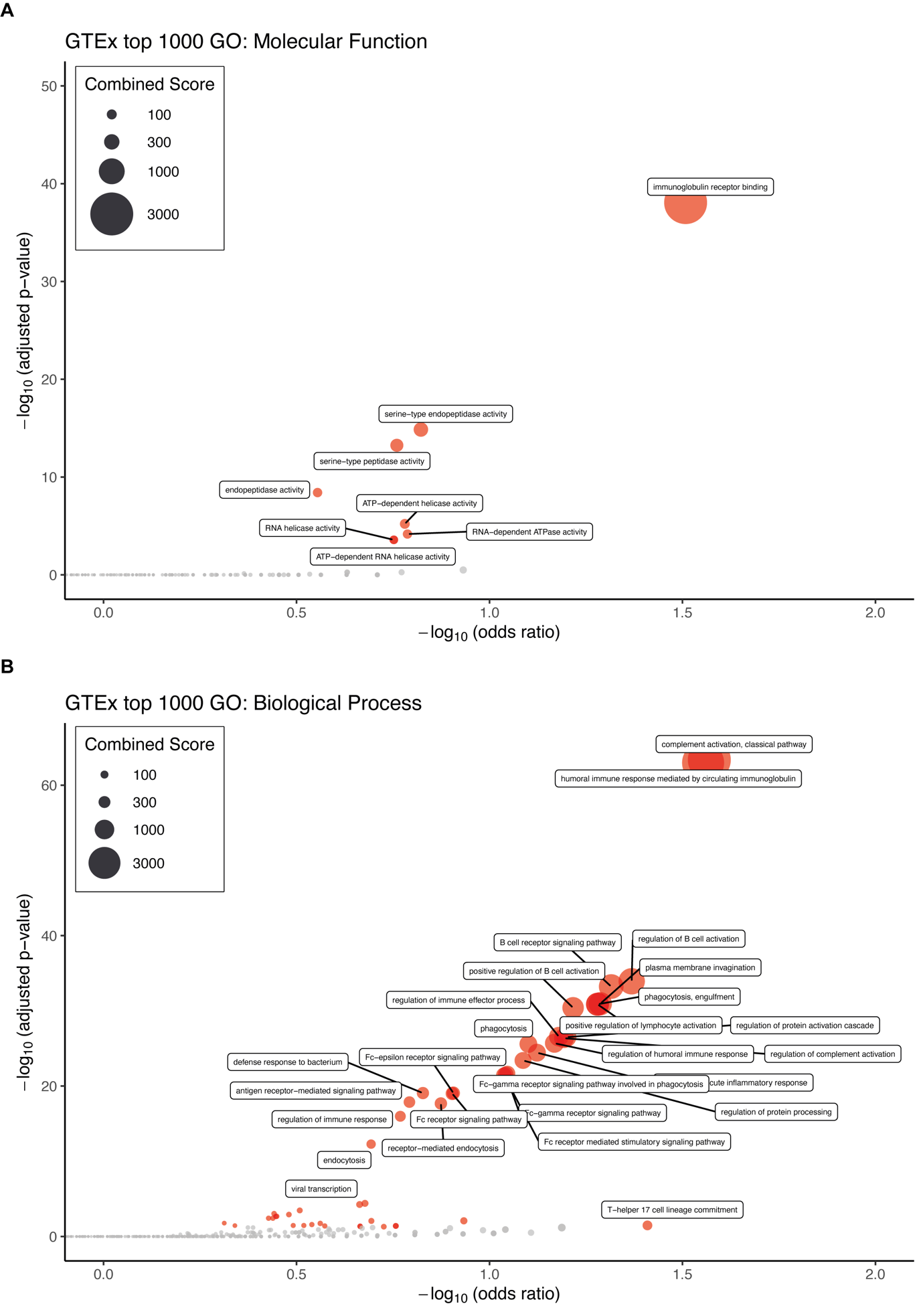


**Fig S9. Bubble plots showing Gene Ontology terms enriched for the 1000 genes whose tissue expression in the GTEx dataset correlates most positively with mtOXPHOS-nuOXPHOS correlation .**

**A)** Enriched terms for the Gene Ontology gene set library ‘Molecular Function’. The odds ratio, reflecting degree of enrichment, is plotted against the adjusted p-value from a Fisher’s exact test. The size of the bubble reflects Enrichr’s combined score, which is the product of the p-value and odds ratio. Red bubbles are significantly enriched at an adjusted p-value < 0.05.

**B)** Enriched terms for the Gene Ontology gene set library ‘Biological Process’. Labels plotted for the 50 terms with highest combined scores. Some labels may be excluded due to lack of space.


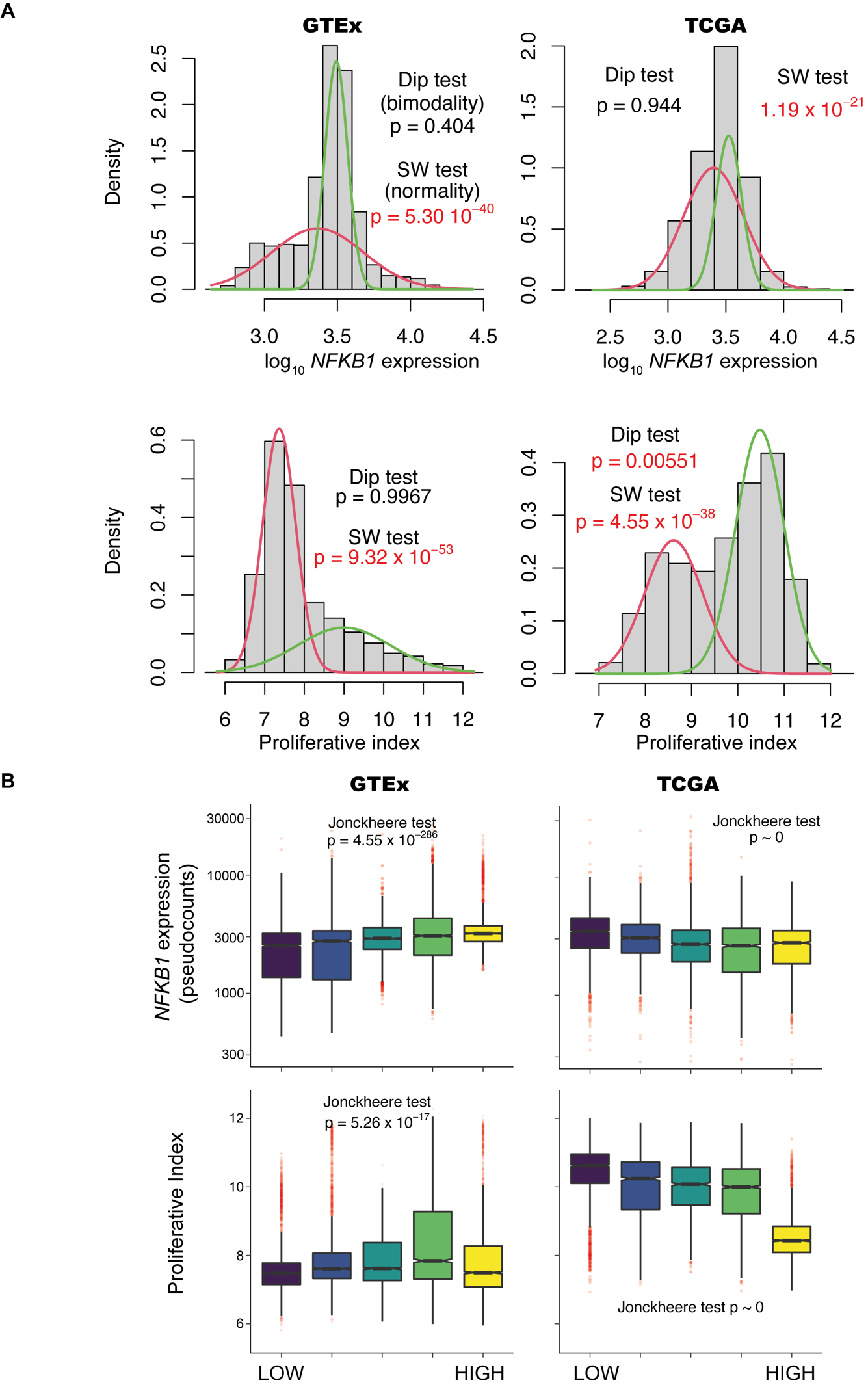
**Fig S10. Non-parametric analysis supports role for *NFKB1* expression in GTEx and proliferation in TCGA mtOXPHOS-nuOXPHOS coordination (related to Fig 5).**

**A)** Density plots of log_10_ *NFKB1* expression or proliferative index (PI) for the GTEx and TCGA samples. To determine if distributions are bimodal, two normal distributions are fitted to the data and the p-value for Hartigan’s dip test for bimodality is shown. To determine if the data is normally distributed, the p-value for Shapiro-Wilk normality test is shown. Clockwise from top-left: GTEx *NFKB1*, TCGA *NFKB1*, TCGA PI, GTEx PI.

**B)** Nonparametric tests for relationship of OXPHOS correlation with *NFKB1* expression or PI. The data is sorted into 5 bins according to OXPHOS correlation; log_10_ *NFKB1* expression or PI is shown for each bin. p-values are shown for the Jonckheere test for trend. Clockwise from top-left: GTEx *NFKB1* (corresponds to Fig 5A), TCGA *NFKB1* (c.f. Fig 5C), TCGA PI (c.f. Fig 5D), GTEx PI (c.f. Fig 5B).

**
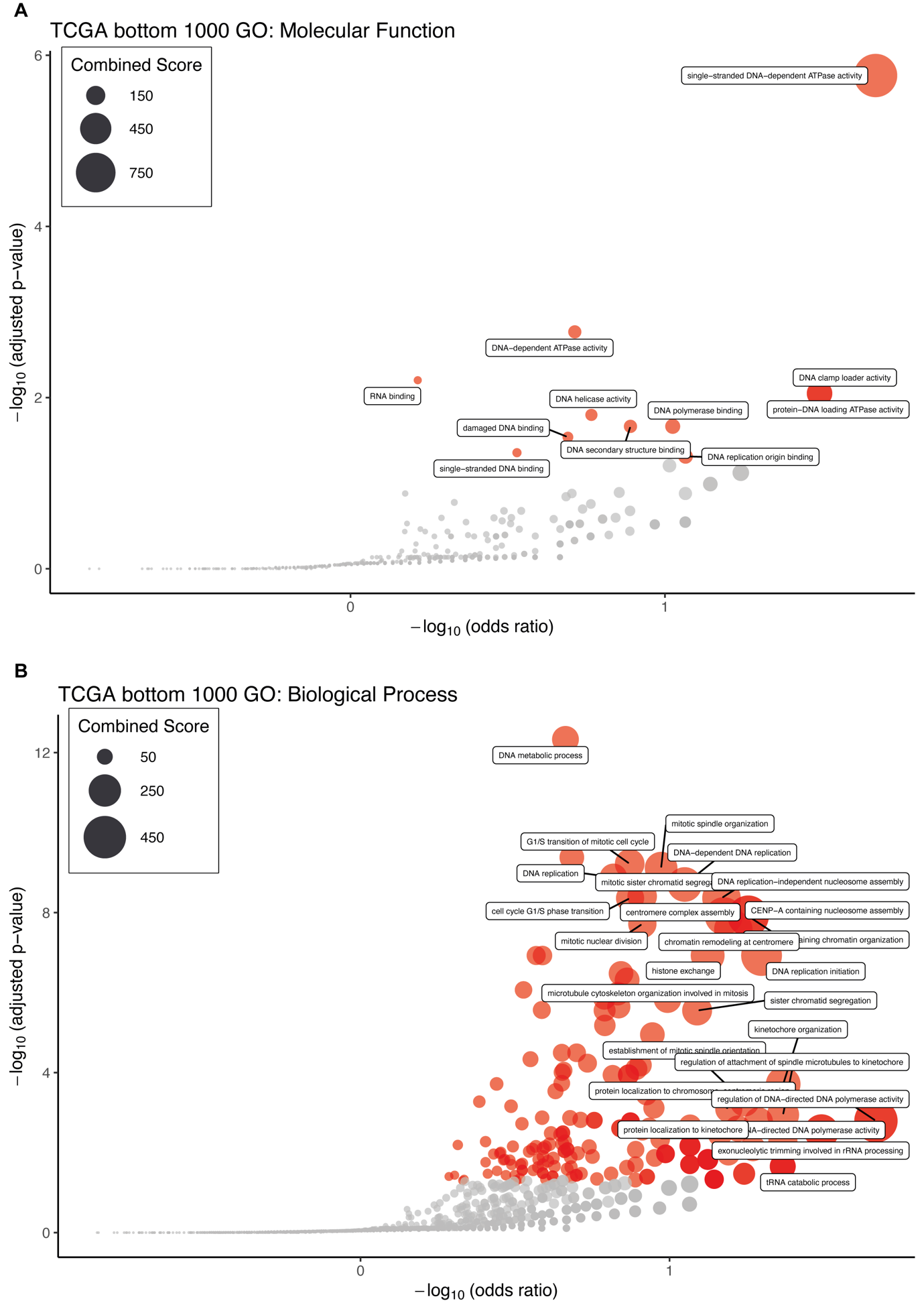
**

**Fig S11. Bubble plots showing proliferation-related Gene Ontology terms enriched for the 1000 genes whose cancer type expression in the TCGA dataset correlates most negatively with mtOXPHOS-nuOXPHOS correlation.**

**A)** Enriched terms for the Gene Ontology gene set library ‘Molecular Function’. The odds ratio, reflecting degree of enrichment, is plotted against the adjusted p-value from a Fisher’s exact test. The size of the bubble reflects Enrichr’s combined score, which is the product of the p-value and odds ratio. Red bubbles are significantly enriched at an adjusted p-value < 0.05.

**B)** Enriched terms for the Gene Ontology gene set library ‘Biological Process’. Labels only applied for terms with a combined score greater than 150. Some labels may be excluded due to lack of space.

**Table S1: Tissue mtOXPHOS-nuOXPHOS Spearman’s correlations for GTEx database relative to median mtOXPHOS-random nuclear gene correlations.**

| **Tissue** | **Observed median**  **mtOXPHOS-nuOXPHOS ρ** | **Mean median**  **mtOXPHOS-random ρ** | **Shapiro-Wilk normality test FDR** | **Z-statistic**  **(observed**  **correlation)** | **FDR** |
| --- | --- | --- | --- | --- | --- |
| Small Intestine - Terminal Ileum | 0.329 | 0.010 | 0.517 | 9.88 | 3.52E-22 |
| Breast - Mammary Tissue | 0.315 | 0.059 | 0.878 | 7.84 | 1.91E-14 |
| Stomach | 0.310 | -0.051 | 0.992 | 11.7 | 1.89E-30 |
| Muscle - Skeletal | 0.305 | -0.010 | 0.946 | 25.3 | 5.16E-139 |
| Cells - EBV-transformed lymphocytes | 0.264 | 0.005 | 0.753 | 12.3 | 9.28E-34 |
| Minor Salivary Gland | 0.184 | 0.006 | 0.100 | 15.9 | 3.18E-55 |
| Adipose - Subcutaneous | 0.177 | 0.016 | 0.656 | 13.2 | 1.02E-38 |
| Colon - Transverse | 0.169 | -0.027 | 0.594 | 5.51 | 9.99E-08 |
| Adipose - Visceral (Omentum) | 0.149 | 0.012 | 0.869 | 9.23 | 1.43E-19 |
| Whole Blood | 0.116 | 0.053 | 0.517 | 2.20 | 3.91E-02 |
| Uterus | 0.111 | 0.010 | 0.878 | 7.79 | 2.74E-14 |
| Artery - Coronary | 0.105 | -0.017 | 0.878 | 8.10 | 2.63E-15 |
| Brain - Spinal cord (cervical c-1) | 0.096 | 0.004 | 0.953 | 3.55 | 7.25E-04 |
| Testis | 0.093 | 0.210 | 0.843 | -4.17 | 7.22E-05 |
| Brain - Cortex | 0.086 | 0.022 | 0.517 | 2.05 | 5.49E-02 |
| Pancreas | 0.083 | -0.011 | 0.926 | 7.05 | 6.67E-12 |
| Vagina | 0.078 | 0.021 | 0.656 | 4.83 | 3.58E-06 |
| Spleen | 0.075 | 0.028 | 0.628 | 4.73 | 5.58E-06 |
| Colon - Sigmoid | 0.063 | 0.002 | 0.953 | 6.00 | 6.29E-09 |
| Ovary | 0.058 | 0.025 | 0.878 | 1.96 | 6.69E-02 |
| Brain - Cerebellar Hemisphere | 0.053 | 0.029 | 0.517 | 0.746 | 4.97E-01 |
| Skin - Sun Exposed (Lower leg) | 0.046 | 0.013 | 0.753 | 1.91 | 7.17E-02 |
| Thyroid | 0.046 | -0.001 | 0.517 | 3.87 | 2.33E-04 |
| Prostate | 0.044 | -0.022 | 0.695 | 3.91 | 2.15E-04 |
| Skin - Not Sun Exposed (Suprapubic) | 0.041 | 0.010 | 0.946 | 1.53 | 1.54E-01 |
| Heart - Atrial Appendage | 0.040 | 0.002 | 0.882 | 3.85 | 2.42E-04 |
| Esophagus - Gastroesophageal Junction | 0.038 | 0.012 | 0.517 | 2.80 | 8.02E-03 |
| Brain - Cerebellum | 0.028 | 0.028 | 0.926 | 0.001 | 1.00E+00 |
| Cells - Cultured fibroblasts | 0.027 | 0.010 | 0.517 | 1.417 | 1.88E-01 |
| Brain - Frontal Cortex (BA9) | 0.012 | 0.053 | 0.992 | -0.989 | 3.69E-01 |
| Esophagus - Muscularis | 0.011 | 0.009 | 0.768 | 0.238 | 8.47E-01 |
| Lung | -0.002 | -0.008 | 0.594 | 0.400 | 7.35E-01 |
| Brain - Hypothalamus | -0.010 | 0.032 | 0.869 | -1.17 | 2.84E-01 |
| Nerve - Tibial | -0.012 | -0.012 | 0.517 | -0.021 | 1.00E+00 |
| Pituitary | -0.024 | 0.042 | 0.882 | -3.68 | 4.62E-04 |
| Brain - Nucleus accumbens (basal ganglia) | -0.025 | 0.079 | 0.517 | -1.91 | 7.17E-02 |
| Brain - Substantia nigra | -0.027 | 0.004 | 0.602 | -0.966 | 3.73E-01 |
| Esophagus - Mucosa | -0.034 | 0.022 | 0.953 | -3.10 | 3.16E-03 |
| Artery - Tibial | -0.036 | -0.006 | 0.869 | -3.48 | 9.02E-04 |
| Artery - Aorta | -0.043 | -0.007 | 0.594 | -3.42 | 1.06E-03 |
| Brain - Amygdala | -0.046 | 0.044 | 0.517 | -2.44 | 2.20E-02 |
| Adrenal Gland | -0.061 | -0.004 | 0.517 | -3.58 | 6.66E-04 |
| Brain - Anterior cingulate cortex (BA24) | -0.066 | 0.045 | 0.992 | -2.35 | 2.73E-02 |
| Brain - Putamen (basal ganglia) | -0.080 | 0.097 | 0.632 | -3.09 | 3.16E-03 |
| Brain - Hippocampus | -0.177 | 0.023 | 0.882 | -6.61 | 1.29E-10 |
| Liver | -0.204 | -0.008 | 0.882 | -10.9 | 8.41E-27 |
| Brain - Caudate (basal ganglia) | -0.206 | 0.071 | 0.602 | -5.62 | 5.89E-08 |
| Heart - Left Ventricle | -0.361 | -0.012 | 0.100 | -9.73 | 1.25E-21 |

**Table S2: Cancer type mtOXPHOS-nuOXPHOS Spearman’s correlations for TCGA database relative to median mtOXPHOS-random nuclear gene correlations.**

| **Cancer** | **Observed median**  **mtOXPHOS-nuOXPHOS ρ** | **Mean median**  **mtOXPHOS-random ρ** | **Shapiro-Wilk normality test FDR** | **Z-statistic**  **(observed**  **correlation)** | **FDR** |
| --- | --- | --- | --- | --- | --- |
| UVM | 0.356 | -0.015 | 0.737 | 17.03 | 2.01E-64 |
| SKCM | 0.349 | -0.014 | 0.737 | 26.9 | 2.05E-157 |
| KIRC | 0.305 | -0.009 | 0.924 | 39.5 | 0.00E+00 |
| KICH | 0.300 | -0.043 | 0.466 | 18.6 | 1.57E-76 |
| KIRP | 0.299 | -0.037 | 0.737 | 11.4 | 1.08E-29 |
| PCPG | 0.255 | -0.021 | 0.581 | 19.8 | 1.15E-86 |
| THCA | 0.234 | -0.029 | 0.347 | 17.9 | 7.54E-71 |
| LGG | 0.209 | -0.013 | 0.581 | 12.8 | 5.40E-37 |
| STAD | 0.206 | -0.026 | 0.581 | 18.9 | 9.38E-79 |
| ACC | 0.195 | -0.042 | 0.896 | 9.95 | 5.83E-23 |
| LAML | 0.171 | -0.005 | 0.234 | 6.72 | 3.39E-11 |
| OV | 0.154 | -0.015 | 0.347 | 15.5 | 6.03E-54 |
| PAAD | 0.151 | -0.012 | 0.679 | 16.2 | 3.88E-58 |
| TGCT | 0.129 | 0.005 | 0.581 | 3.25 | 1.40E-03 |
| BRCA | 0.128 | -0.006 | 0.581 | 14.2 | 3.23E-45 |
| LIHC | 0.122 | -0.023 | 0.737 | 10.7 | 4.14E-26 |
| SARC | 0.092 | -0.020 | 0.924 | 9.53 | 3.16E-21 |
| THYM | 0.089 | -0.027 | 0.737 | 4.76 | 2.54E-06 |
| UCEC | 0.085 | 0.014 | 0.581 | 5.00 | 7.94E-07 |
| LUAD | 0.073 | -0.035 | 0.581 | 6.53 | 1.24E-10 |
| BLCA | 0.066 | -0.028 | 0.234 | 4.91 | 1.24E-06 |
| LUSC | 0.056 | -0.022 | 0.679 | 5.64 | 2.69E-08 |
| ESCA | 0.047 | -0.011 | 0.924 | 5.72 | 1.72E-08 |
| PRAD | 0.046 | -0.032 | 0.873 | 3.52 | 5.31E-04 |
| CESC | 0.030 | -0.019 | 0.581 | 5.24 | 2.40E-07 |
| HNSC | 0.027 | -0.042 | 0.737 | 5.77 | 1.37E-08 |
| COAD | 0.014 | 0.001 | 0.234 | 0.658 | 5.46E-01 |
| UCS | -0.016 | -0.009 | 0.821 | -0.274 | 8.10E-01 |
| MESO | -0.023 | -0.004 | 0.737 | -1.10 | 2.98E-01 |
| READ | -0.029 | -0.035 | 0.622 | 0.241 | 8.10E-01 |
| GBM | -0.048 | 0.000 | 0.581 | -2.23 | 2.96E-02 |

**Table S3: Coefficients of a linear model predicting tissue mtOXPHOS-nuOXPHOS correlation by GTEx sample Proliferative Index (PI) and expression of NF-κB family members.**

|  | Estimate | Std. Error | t value | Pr(>\|t\|) |
| --- | --- | --- | --- | --- |
| Intercept | -0.425181 | 0.024484 | -17.366 | < 2e-16 |
| Proliferative Index | -0.005170 | 0.001053 | -4.910 | 9.18e-07 |
| log_10_(*NFKB1* expression) | 0.187359 | 0.007624 | 24.574 | < 2e-16 |
| log_10_(*NFKB2* expression) | -0.077582 | 0.005614 | -13.819 | < 2e-16 |
| log_10_(*REL* expression) | 0.033591 | 0.003987 | 8.425 | < 2e-16 |
| log_10_(*RELA* expression) | -0.038051 | 0.008690 | -4.379 | 1.20e-05 |
| log_10_(*RELB* expression) | 0.068737 | 0.005065 | 13.570 | < 2e-16 |

Residual standard error: 0.1254 on 17237 degrees of freedom

Multiple R-squared: 0.1235, Adjusted R-squared: 0.1232

F-statistic: 404.7 on 6 and 17237 DF, p-value: < 2.2e-16

**Table S4: Coefficients of a linear model predicting cancer type mtOXPHOS-nuOXPHOS correlation by TCGA sample Proliferative Index (PI) and expression of NF-κB family members.**

|  | Estimate | Std. Error | t value | Pr(>\|t\|) |
| --- | --- | --- | --- | --- |
| Intercept | 0.7654839 | 0.0251287 | 30.463 | < 2e-16 |
| Proliferative Index | -0.0458482 | 0.0008316 | -55.135 | < 2e-16 |
| log_10_(*NFKB1* expression) | -0.0310699 | 0.0047090 | -6.598 | 4.38e-11 |
| log_10_(*NFKB2* expression) | 0.0412251 | 0.0043534 | 9.470 | < 2e-16 |
| log_10_(*REL* expression) | -0.0019117 | 0.0023865 | -0.801 | 0.423 |
| log_10_(*RELA* expression) | -0.0417820 | 0.0062731 | -6.661 | 2.88e-11 |
| log_10_(*RELB* expression) | -0.0227510 | 0.0046963 | -4.844 | 1.29e-06 |

Residual standard error: 0.08055 on 9714 degrees of freedom

Multiple R-squared: 0.294, Adjusted R-squared: 0.2935

F-statistic: 674.1 on 6 and 9714 DF, p-value: < 2.2e-16

**Table S5: TCGA cancer type codes.**

| **TCGA abbreviation** | **Cancer type** |
| --- | --- |
| LAML | Acute myeloid leukemia |
| ACC | Adrenocortical carcinoma |
| BLCA | Bladder urothelial carcinoma |
| LGG | Brain lower grade glioma |
| BRCA | Breast invasive carcinoma |
| CESC | Cervical squamous cell carcinoma and endocervical adenocarcinoma |
| COAD | Colon adenocarcinoma |
| ESCA | Esophageal carcinoma |
| GBM | Glioblastoma multiforme |
| HNSC | Head and neck squamous cell carcinoma |
| KICH | Kidney chromophobe |
| KIRC | Kidney renal clear cell carcinoma |
| KIRP | Kidney renal papillary cell carcinoma |
| LIHC | Liver hepatocellular carcinoma |
| LUAD | Lung adenocarcinoma |
| LUSC | Lung squamous cell carcinoma |
| MESO | Mesothelioma |
| OV | Ovarian serous cystadenocarcinoma |
| PAAD | Pancreatic adenocarcinoma |
| PCPG | Pheochromocytoma and paraganglioma |
| PRAD | Prostate adenocarcinoma |
| READ | Rectum adenocarcinoma |
| SARC | Sarcoma |
| SKCM | Skin cutaneous melanoma |
| STAD | Stomach adenocarcinoma |
| TGCT | Testicular germ cell tumors |
| THYM | Thymoma |
| THCA | Thyroid carcinoma |
| UCS | Uterine carcinosarcoma |
| UCEC | Uterine corpus endometrial carcinoma |
| UVM | Uveal melanoma |
