# Supplementary material for: Malignancy and NF-κB signalling strengthen coordination between expression of mitochondrial and nuclear-encoded oxidative phosphorylation genes
Source: Genome Biol. 2021 Dec 2;22:328. doi: 10.1186/s13059-021-02541-6 (PMC8638269; doi:10.1186/s13059-021-02541-6)
Supplement: Supplementary file 2 — Additional File 2. GTEx mtOXPHOS-nuOXPHOS heatmaps. mtOXPHOS-nuOXPHOS expression heatmaps using MRN normalization for 48 GTEx tissues. [file 13059_2021_2541_MOESM2_ESM.pdf]

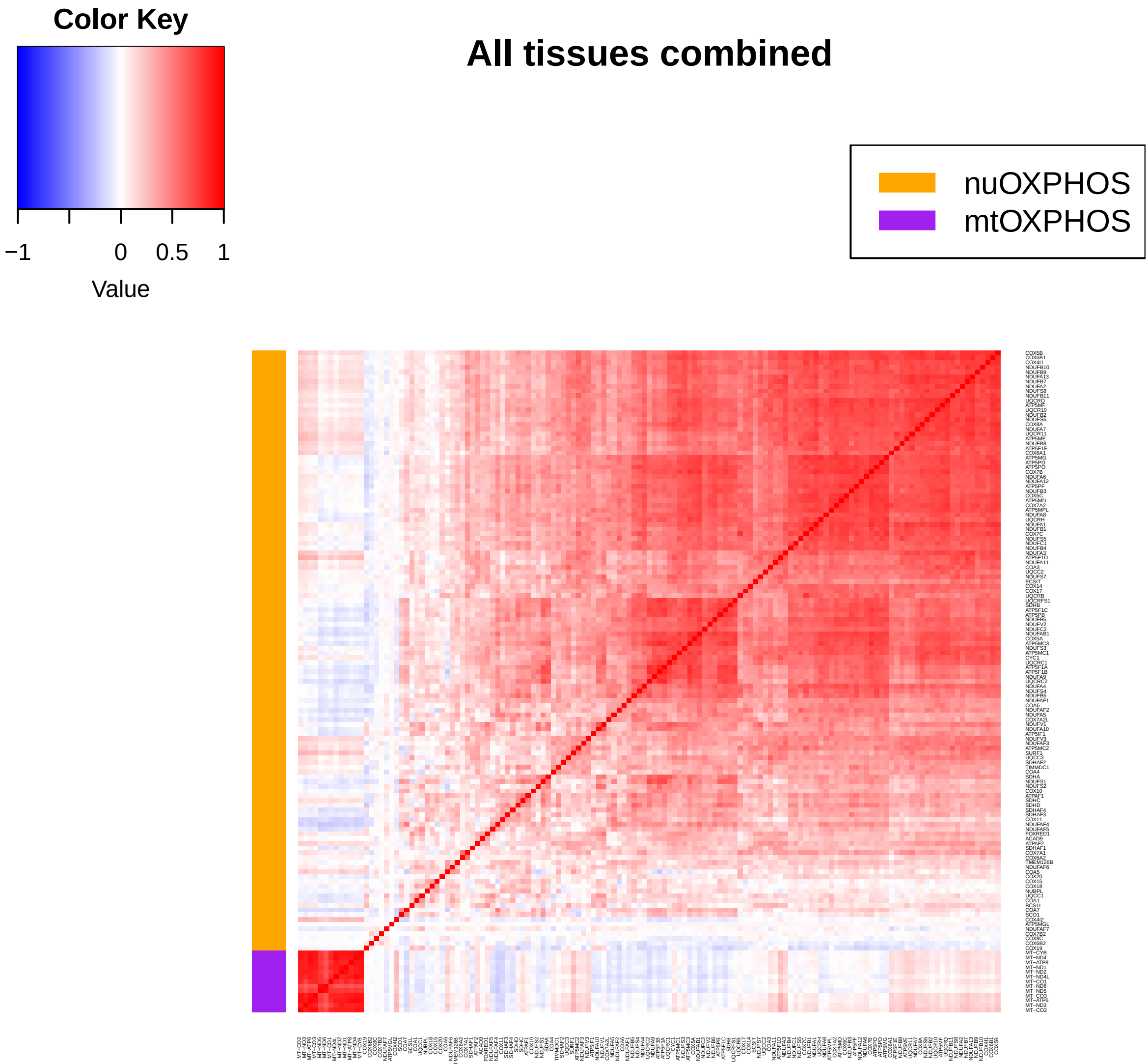
Color Key
−1
0
0.5
1
Value
All tissues combined
nuOXPHOS
mtOXPHOS

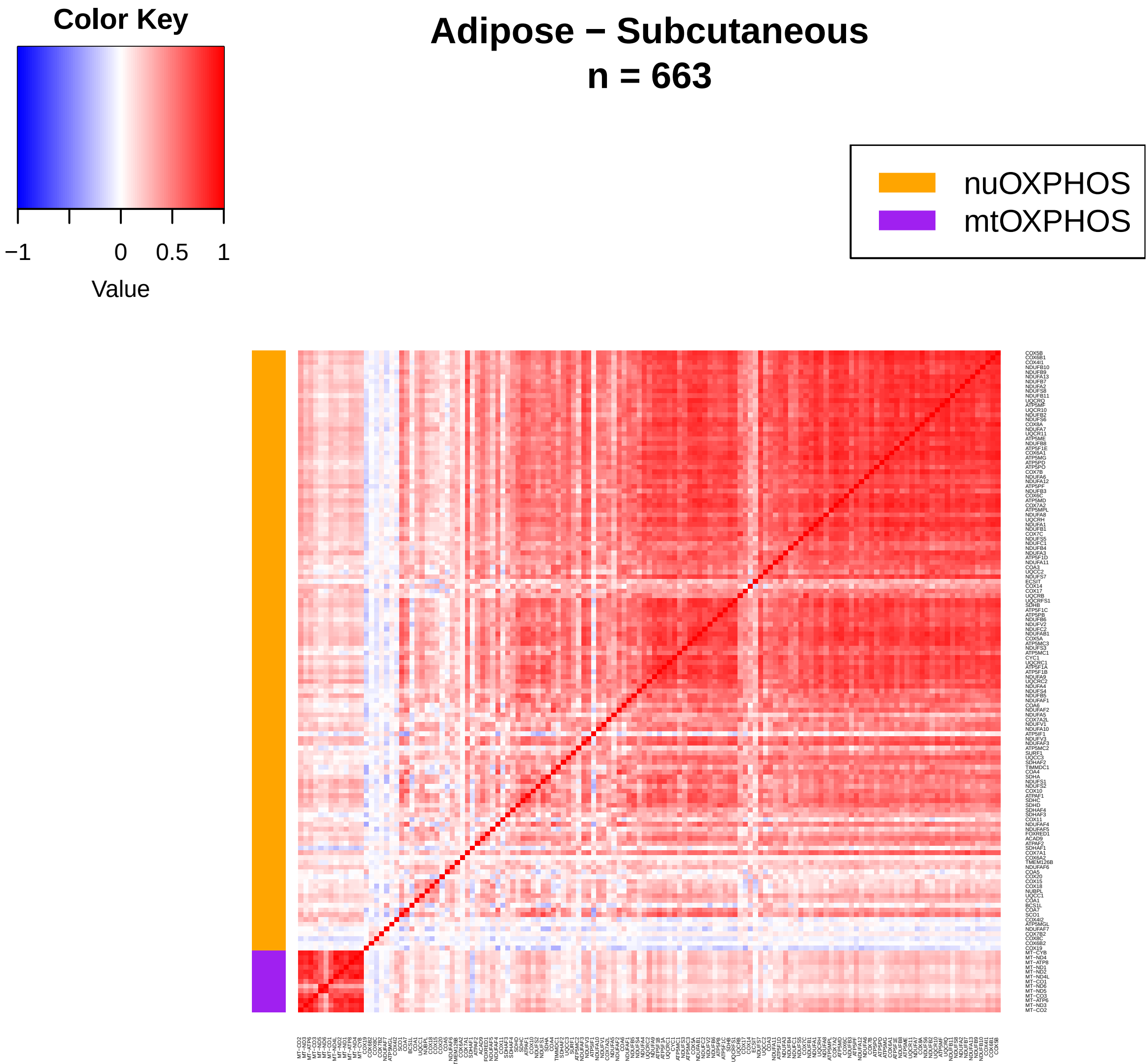
Color Key
−1
0
0.5
1
Value
Adipose − Subcutaneous
n = 663
nuOXPHOS
mtOXPHOS

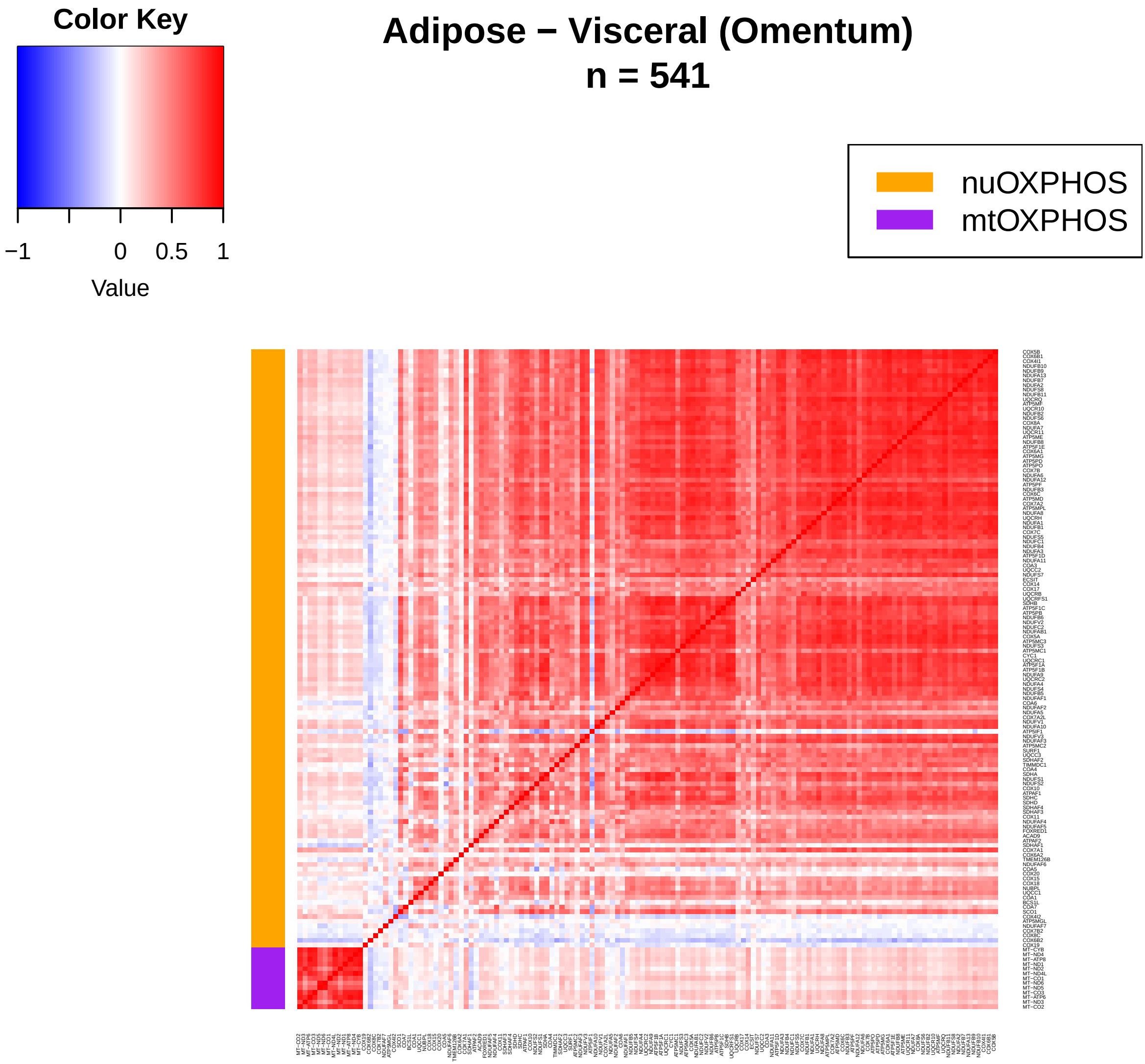
Color Key
−1
0
0.5
1
Value
Adipose − Visceral (Omentum)
n = 541
nuOXPHOS
mtOXPHOS

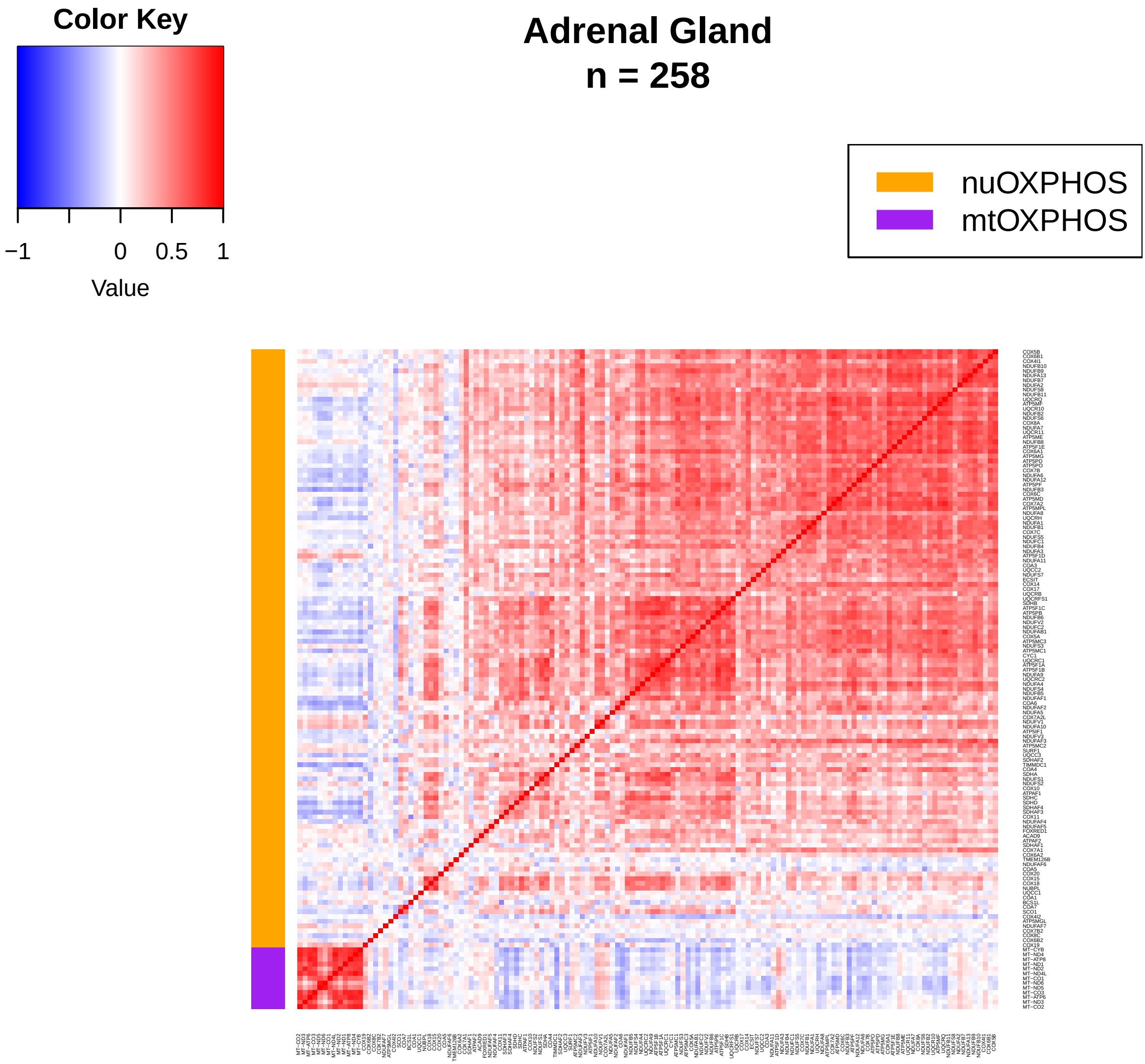
Color Key
−1
0
0.5
1
Value
Adrenal Gland
n = 258
nuOXPHOS
mtOXPHOS

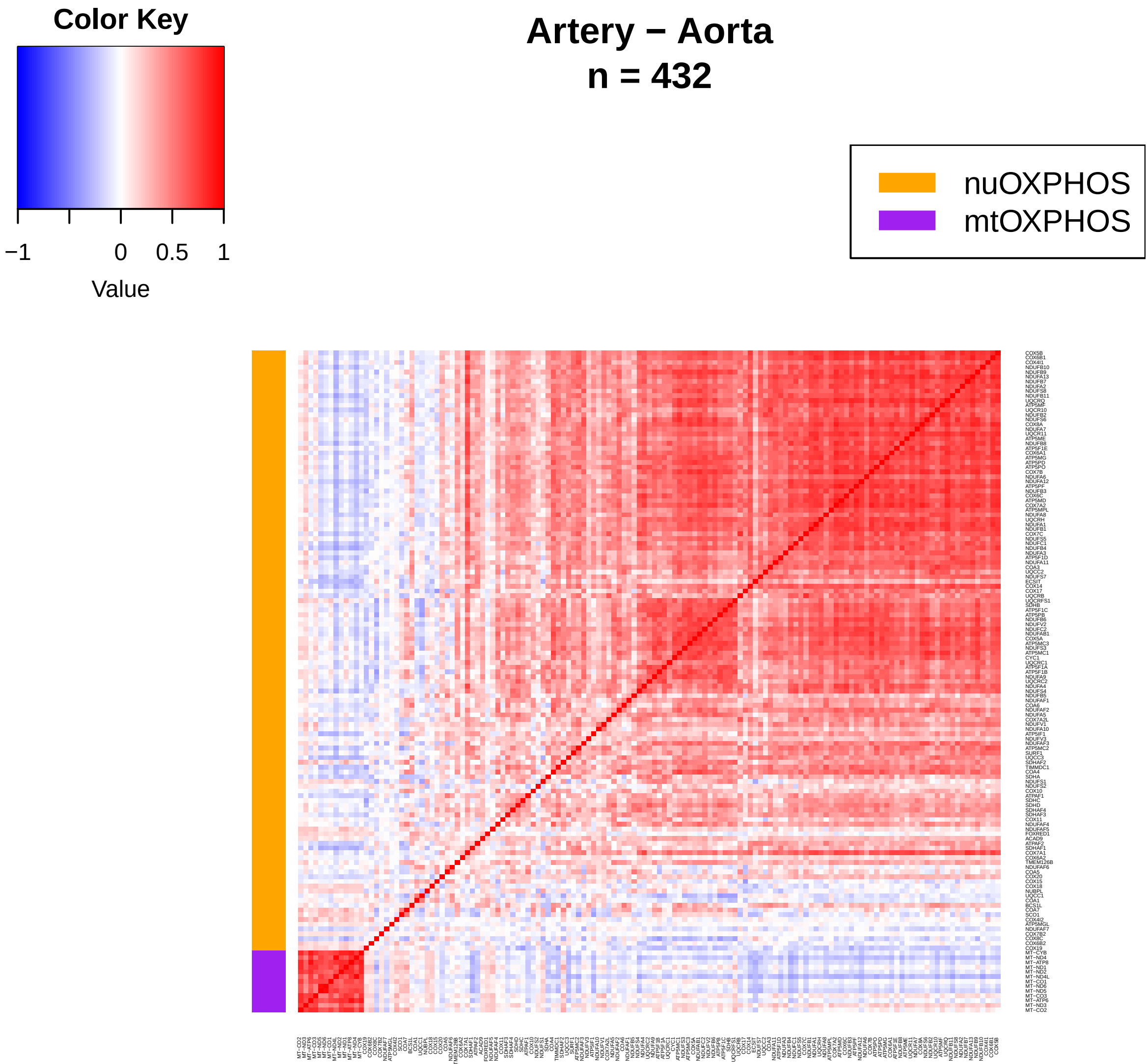

Color Key
−1
0
0.5
1
Value
Artery − Aorta
n = 432
nuOXPHOS
mtOXPHOS

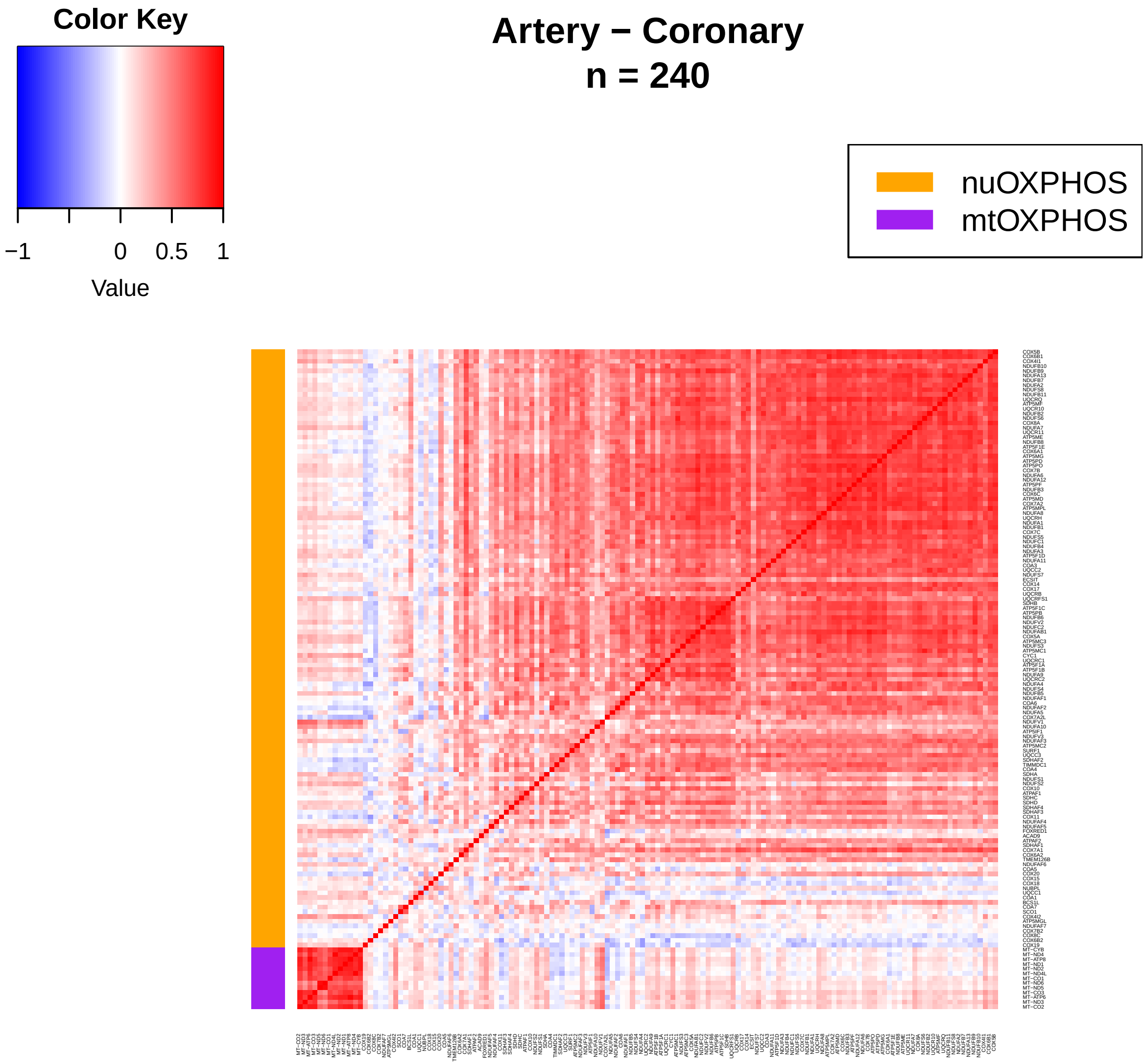
Color Key
−1
0
0.5
1
Value
Artery − Coronary
n = 240
nuOXPHOS
mtOXPHOS

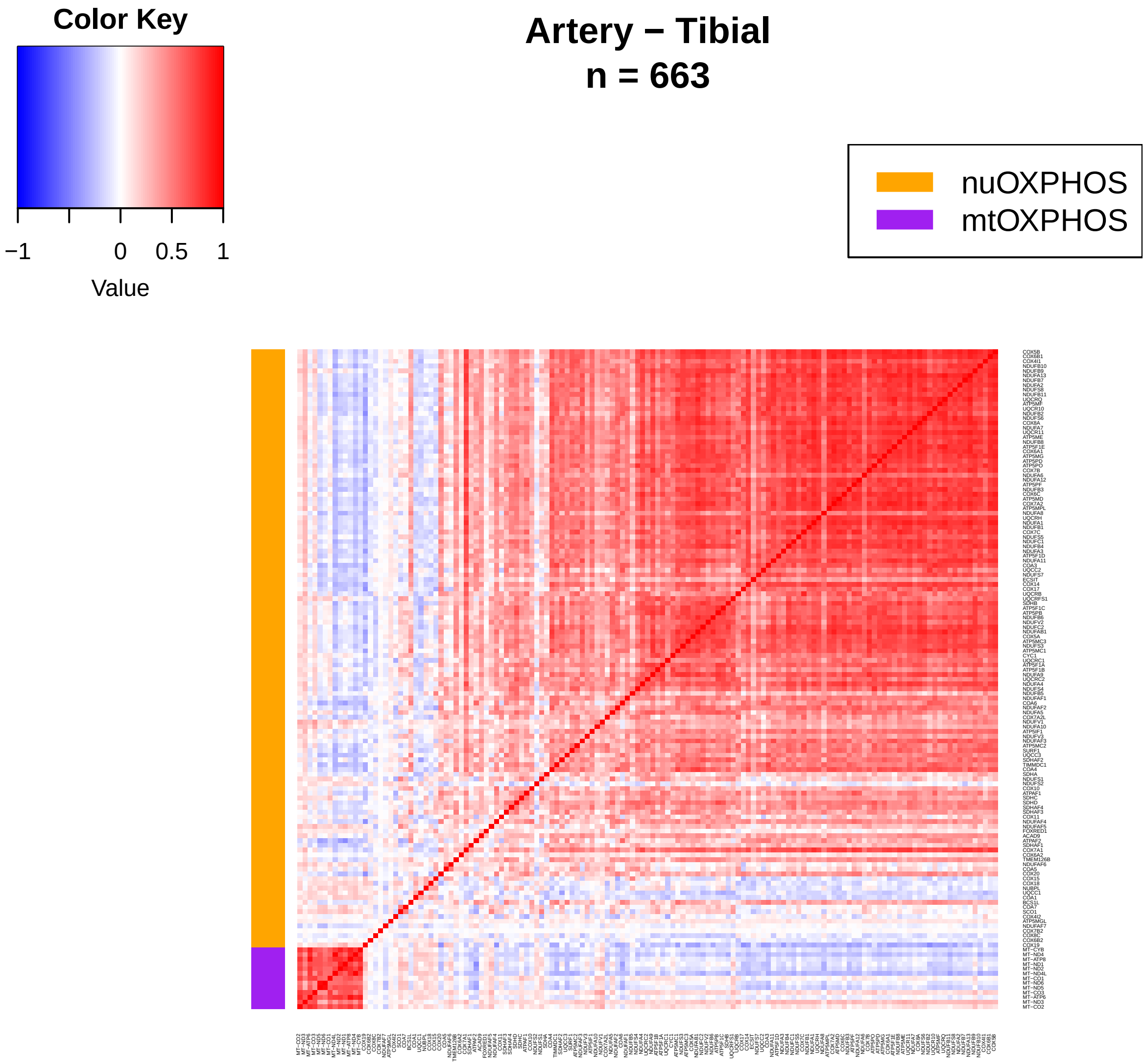
Artery − Tibial
n = 663
Color Key
−1
0
0.5
1
Value
nuOXPHOS
mtOXPHOS

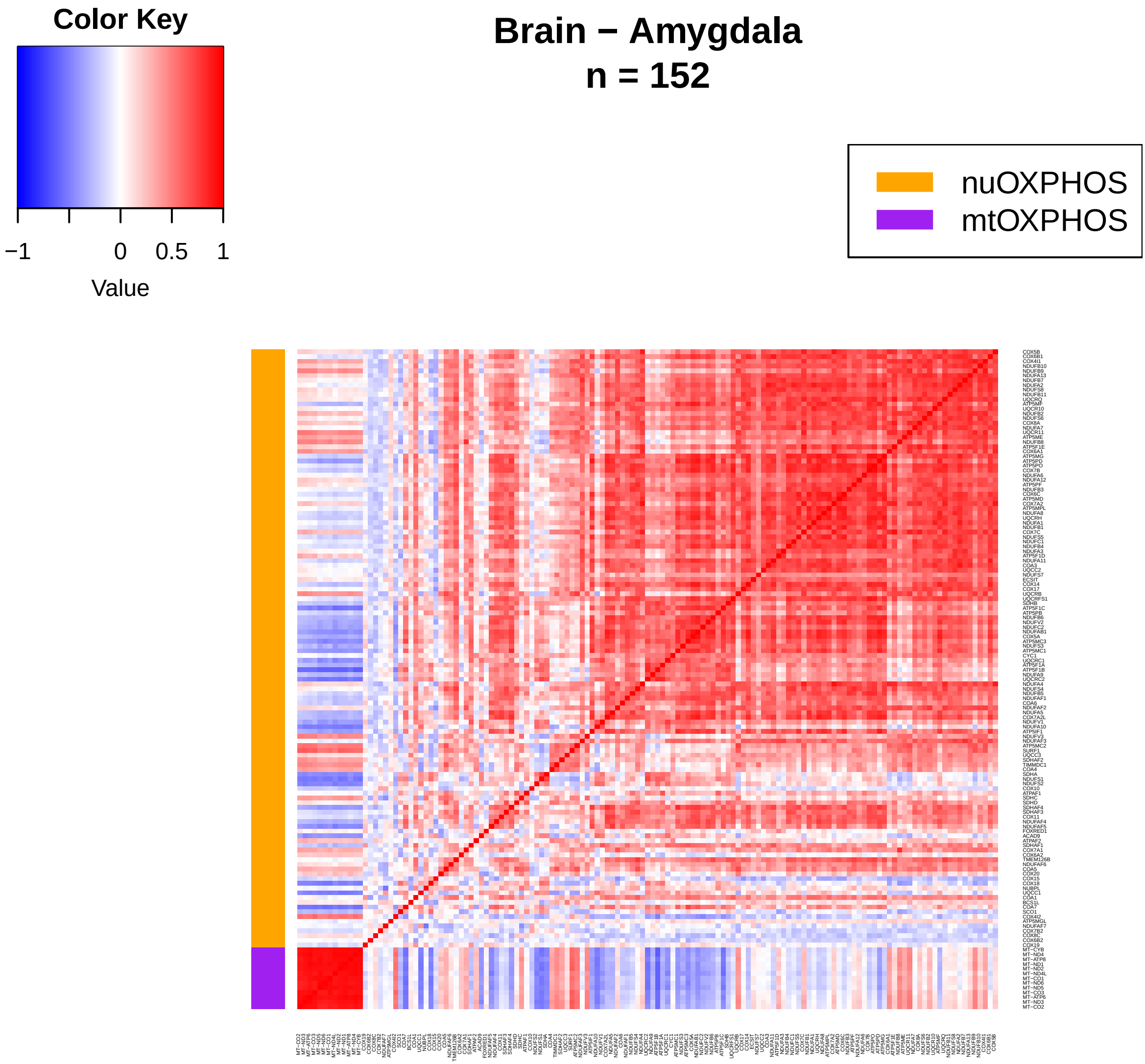
Color Key
−1
0
0.5
1
Value
Brain − Amygdala
n = 152
nuOXPHOS
mtOXPHOS

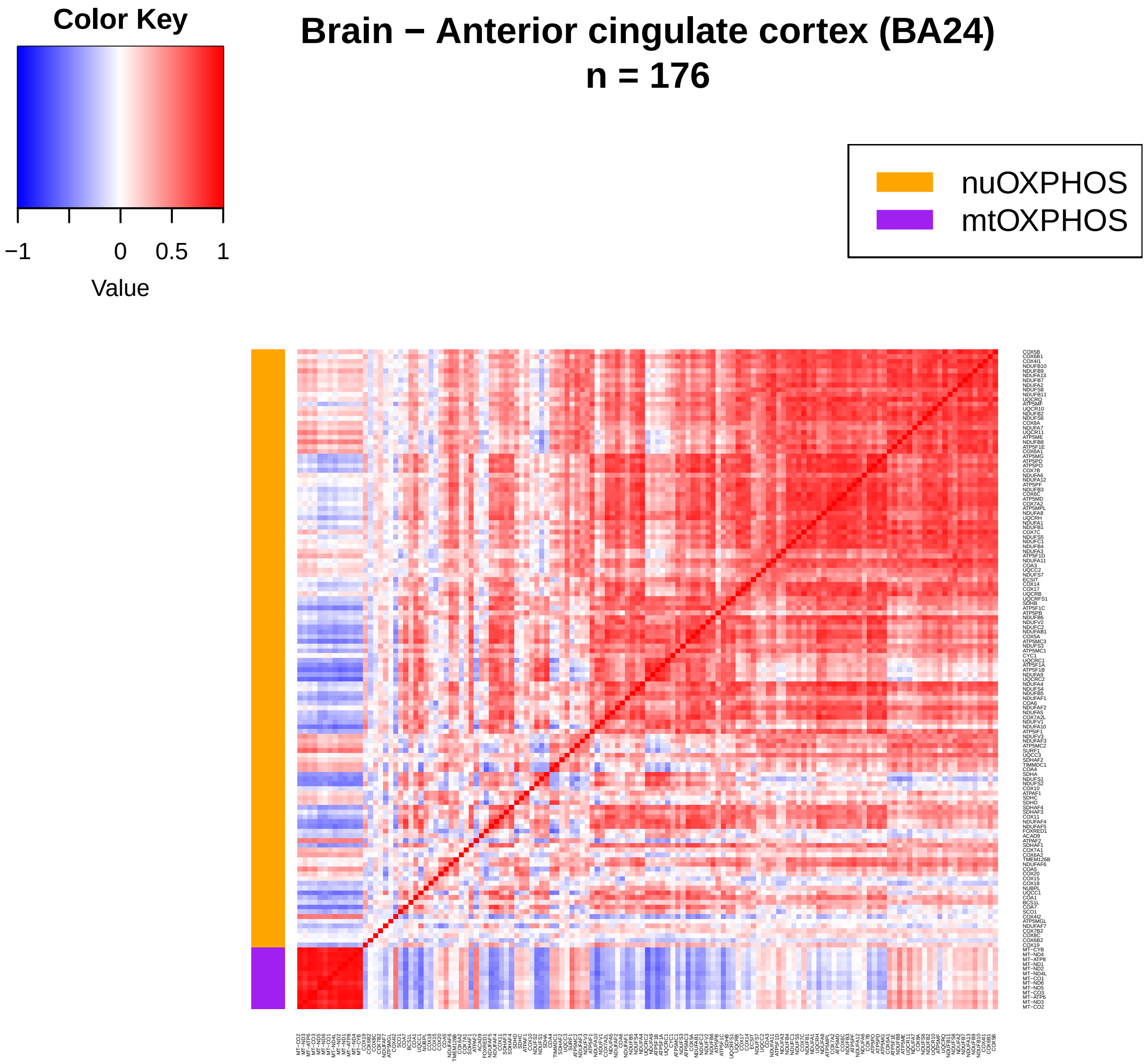
Color Key
−1
0
0.5
1
Value
Brain − Anterior cingulate cortex (BA24)
n = 176
nuOXPHOS
mtOXPHOS

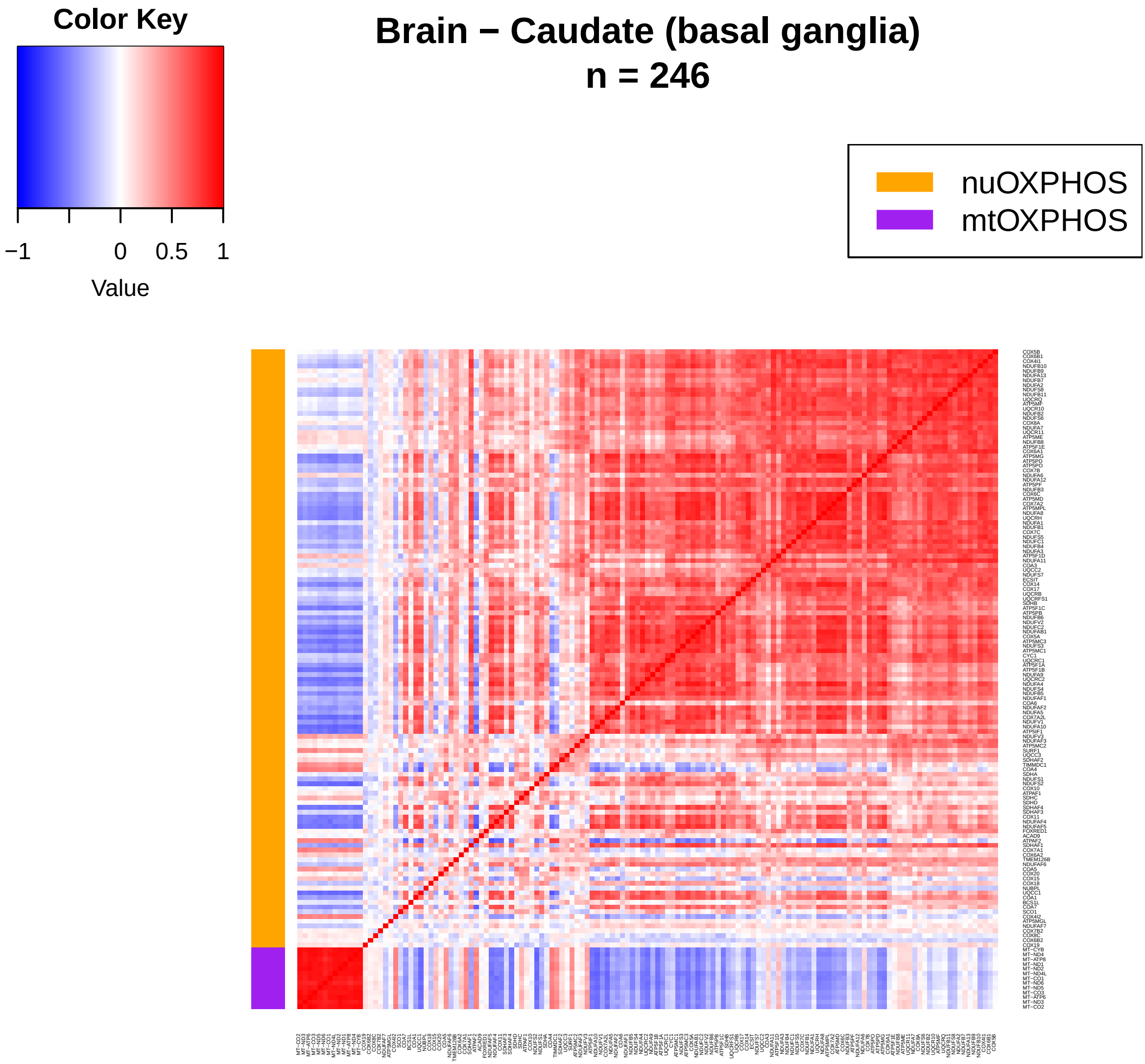
Color Key
−1
0
0.5
1
Value
Brain − Caudate (basal ganglia)
n = 246
nuOXPHOS
mtOXPHOS

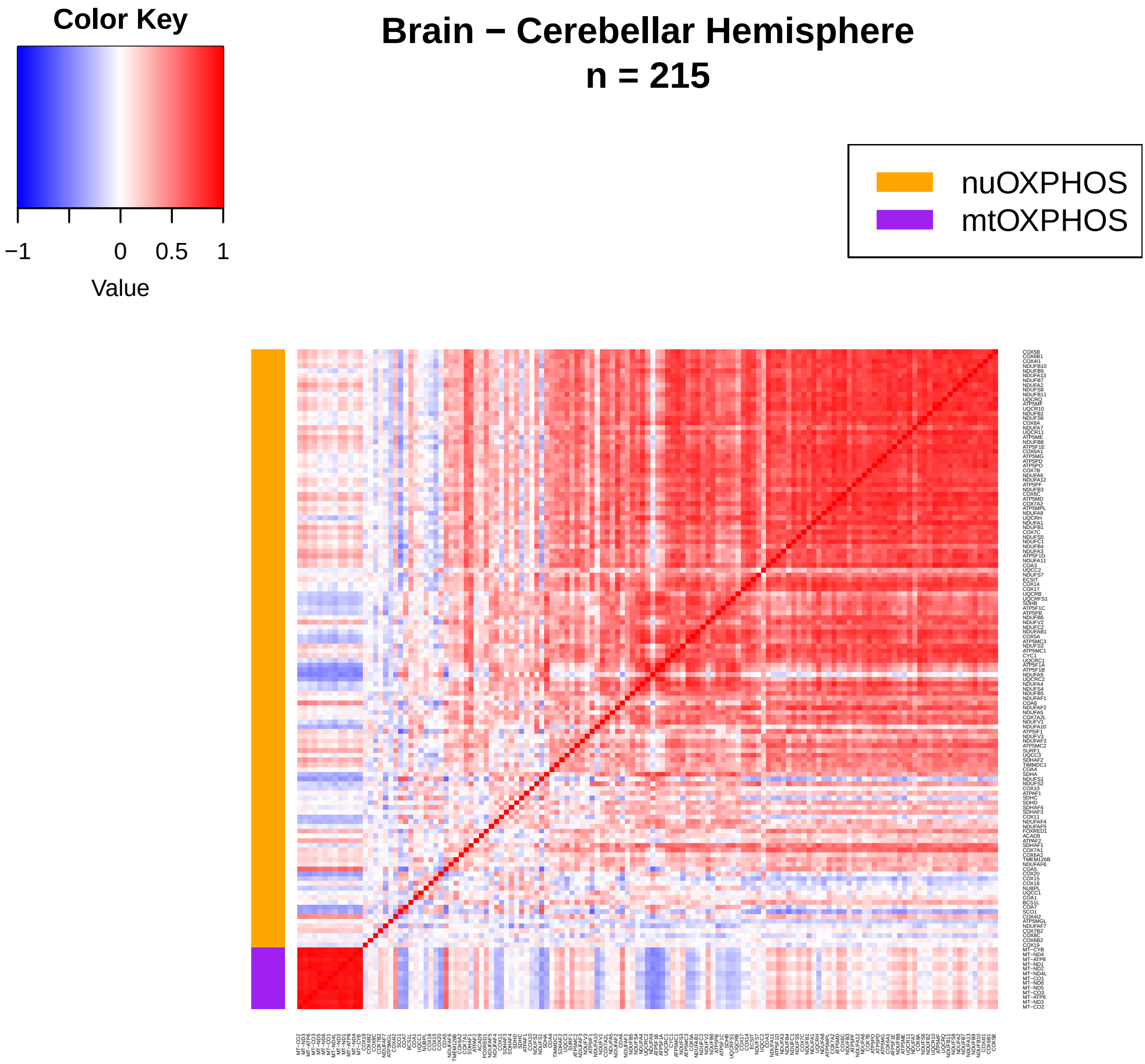
Color Key
−1
0
0.5
1
Value
Brain − Cerebellar Hemisphere
n = 215
nuOXPHOS
mtOXPHOS

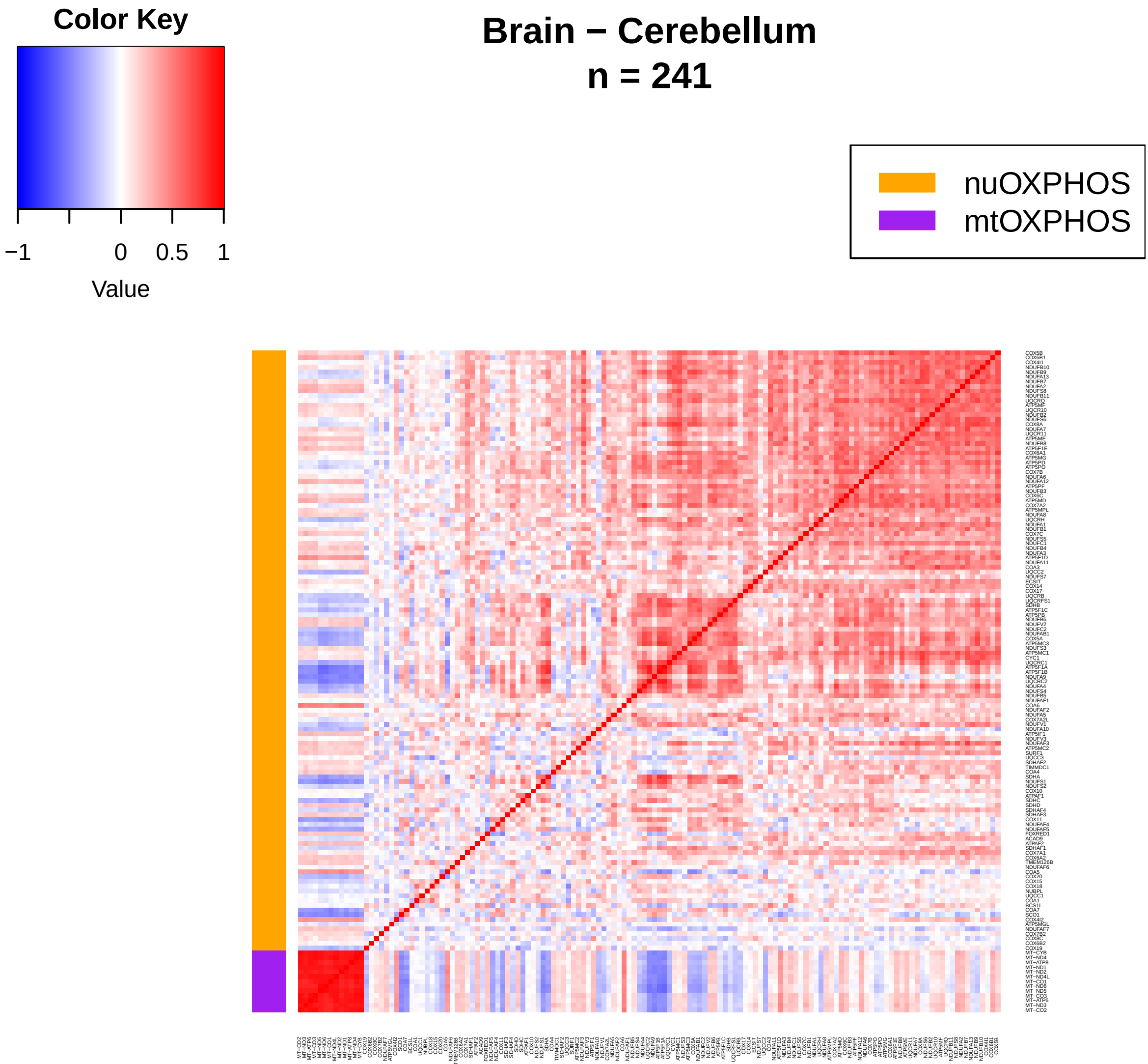
Color Key
−1
0
0.5
1
Value
Brain − Cerebellum
n = 241
nuOXPHOS
mtOXPHOS

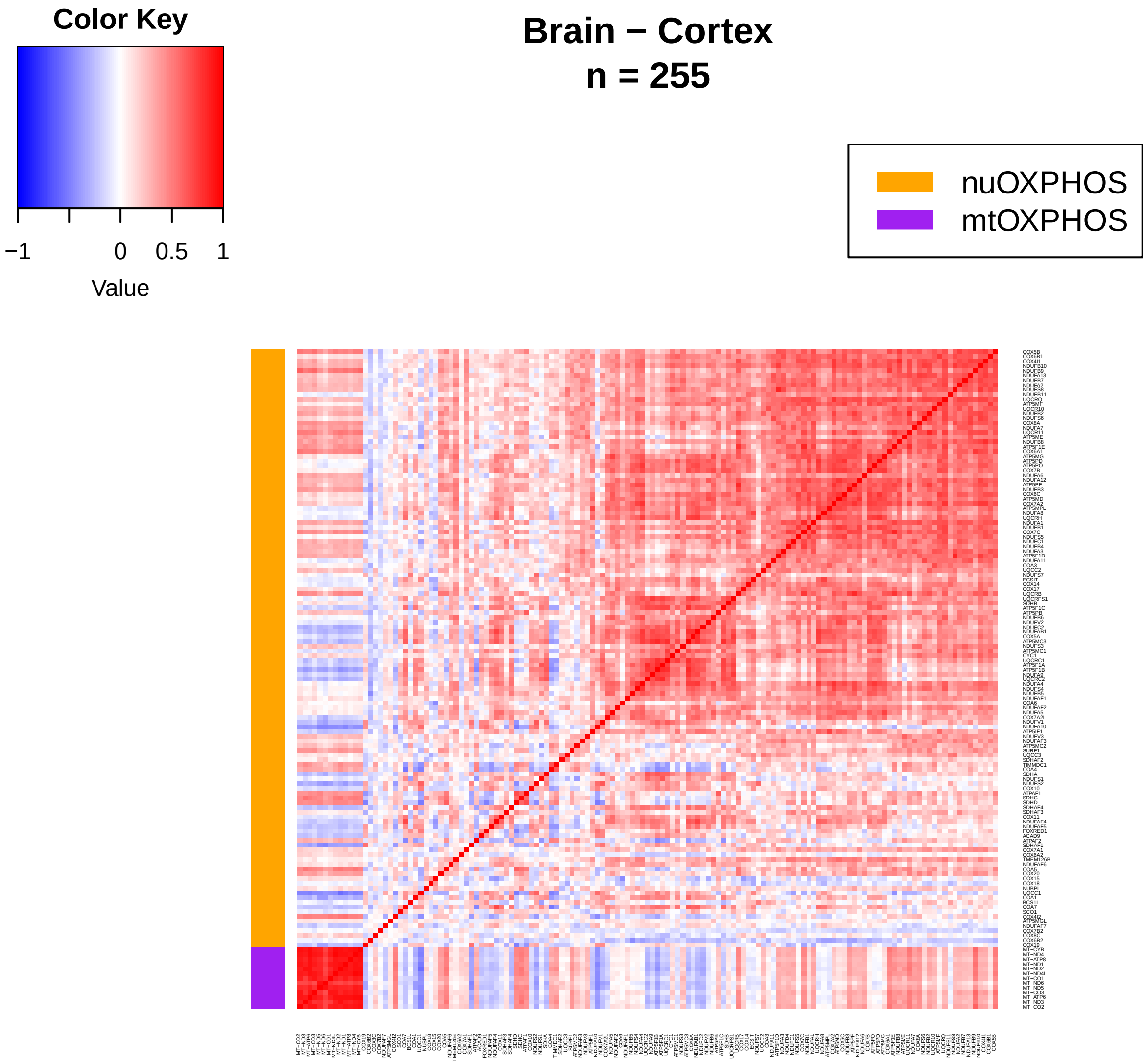
Color Key
−1
0
0.5
1
Value
Brain − Cortex
n = 255
nuOXPHOS
mtOXPHOS

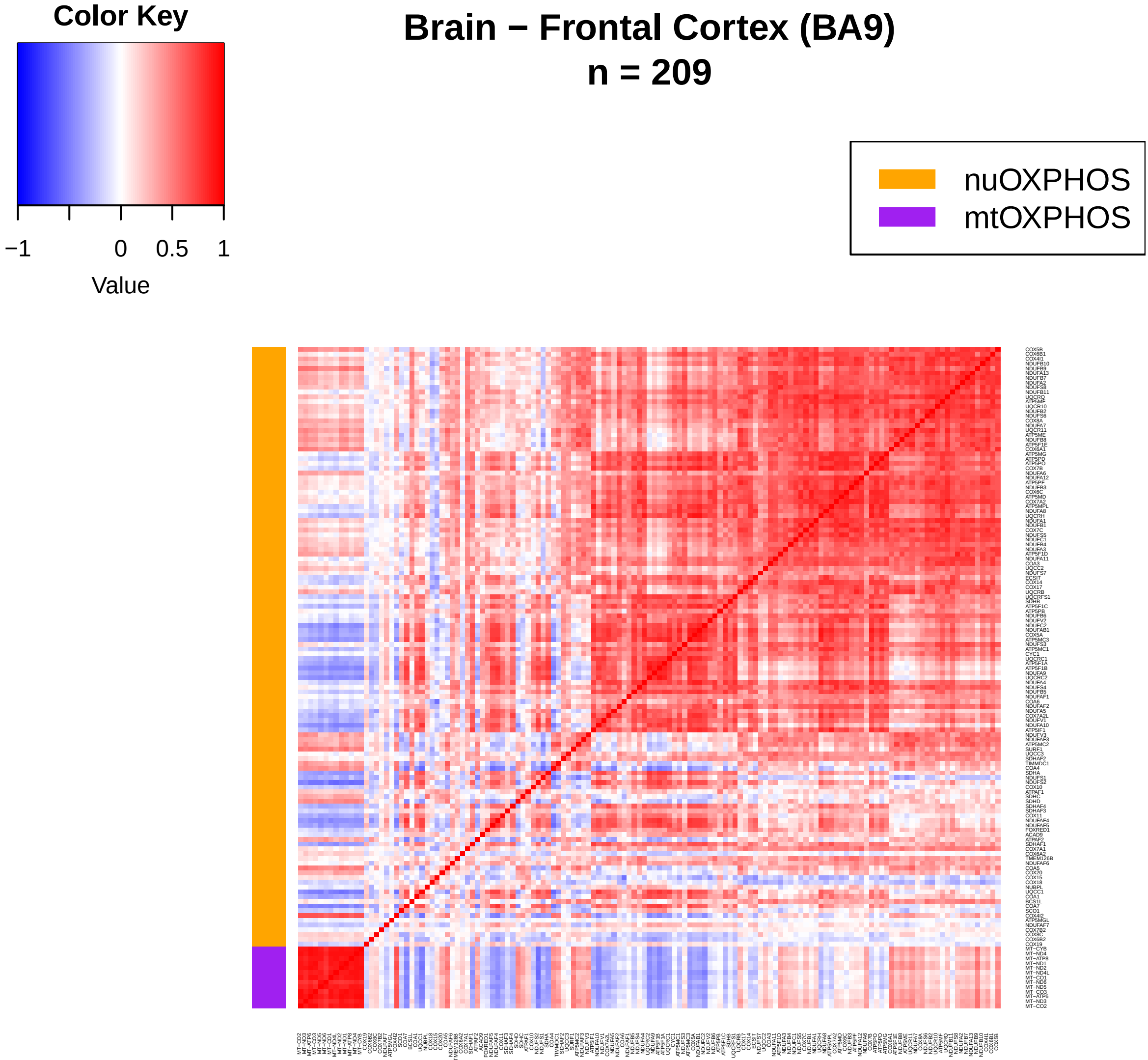
Color Key
−1
0
0.5
1
Value
Brain − Frontal Cortex (BA9)
n = 209
nuOXPHOS
mtOXPHOS

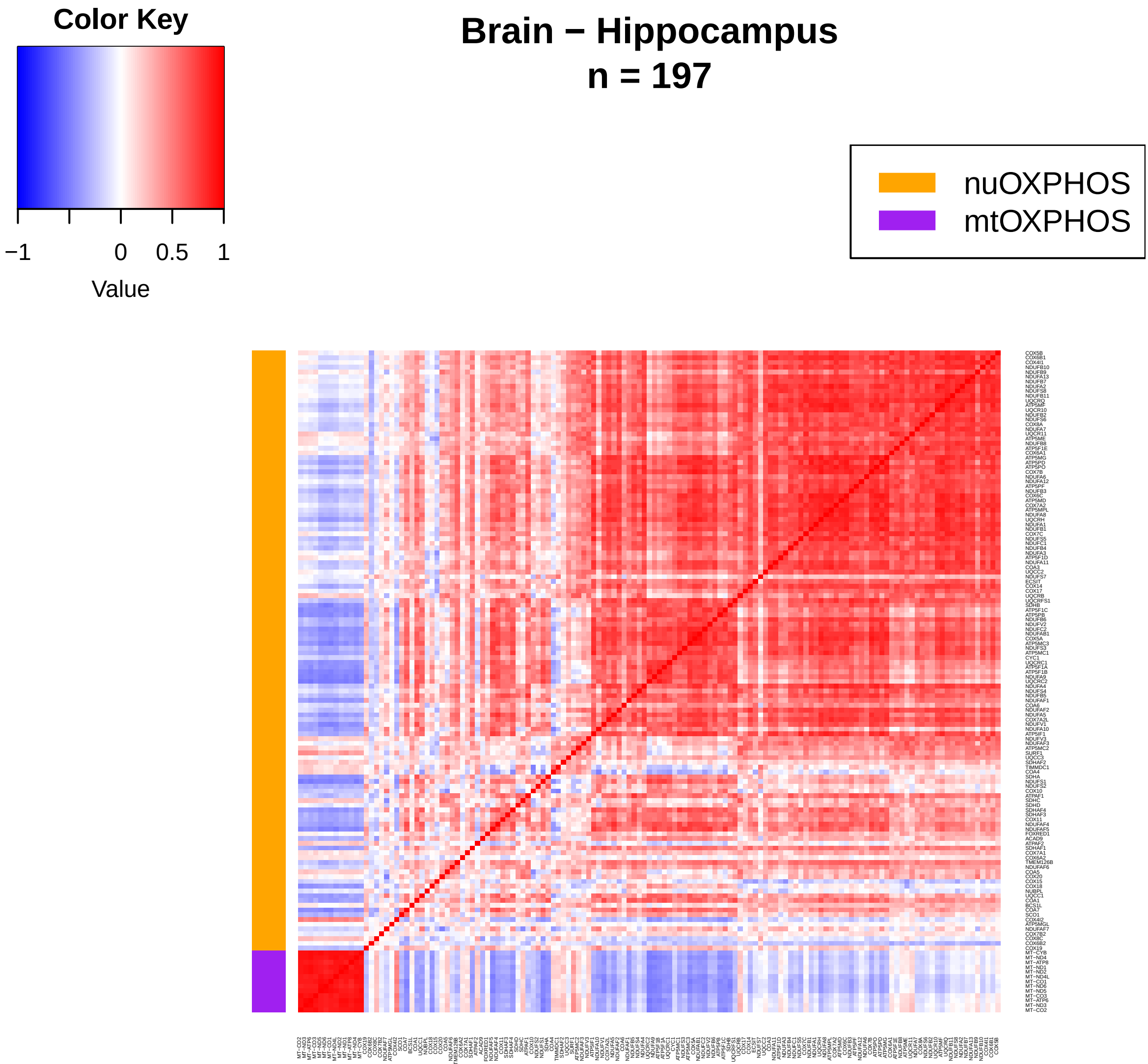
Color Key
−1
0
0.5
1
Value
Brain − Hippocampus
n = 197
nuOXPHOS
mtOXPHOS

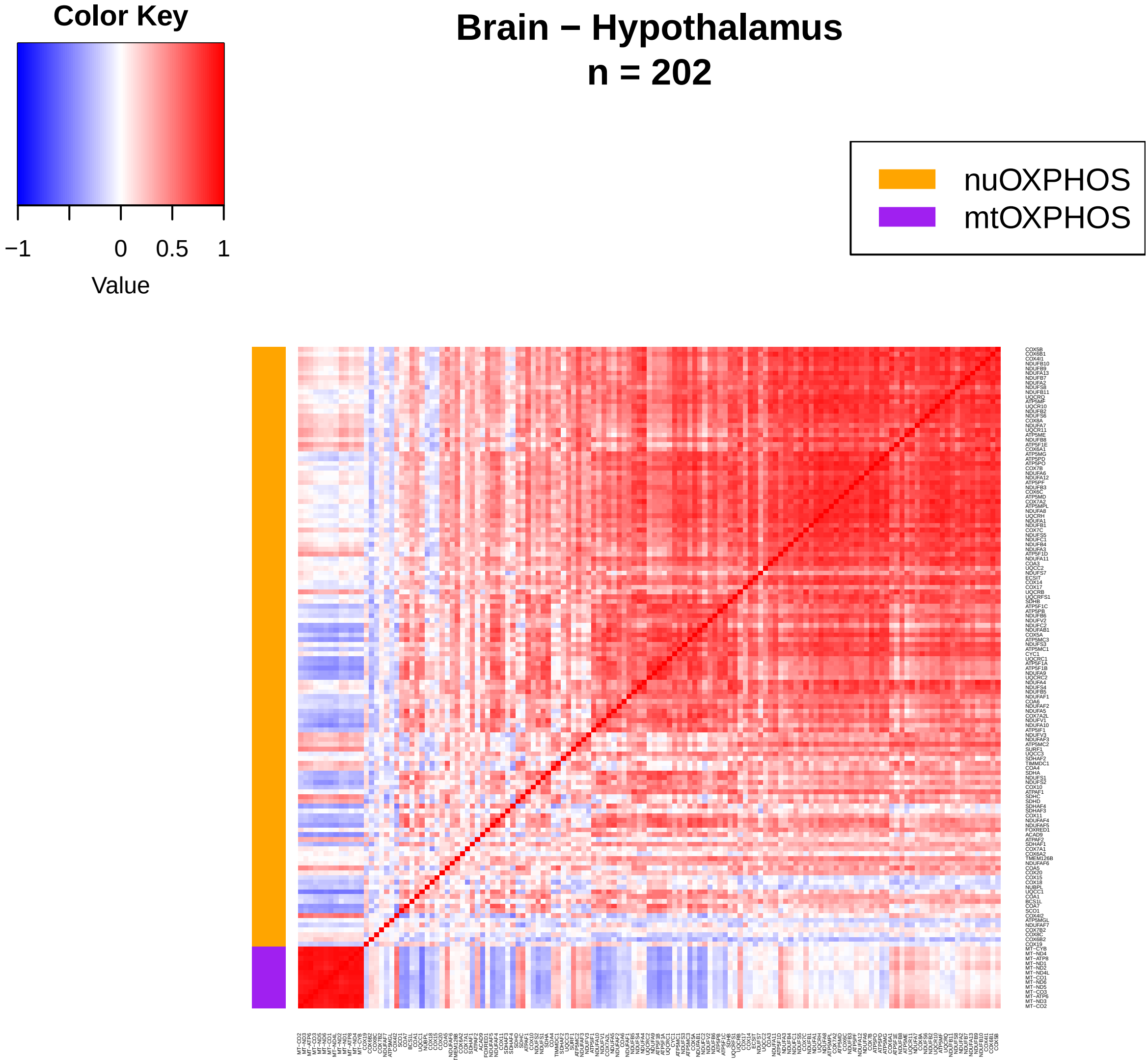
Brain − Hypothalamus
n = 202
Color Key
−1
0
0.5
1
Value
nuOXPHOS
mtOXPHOS

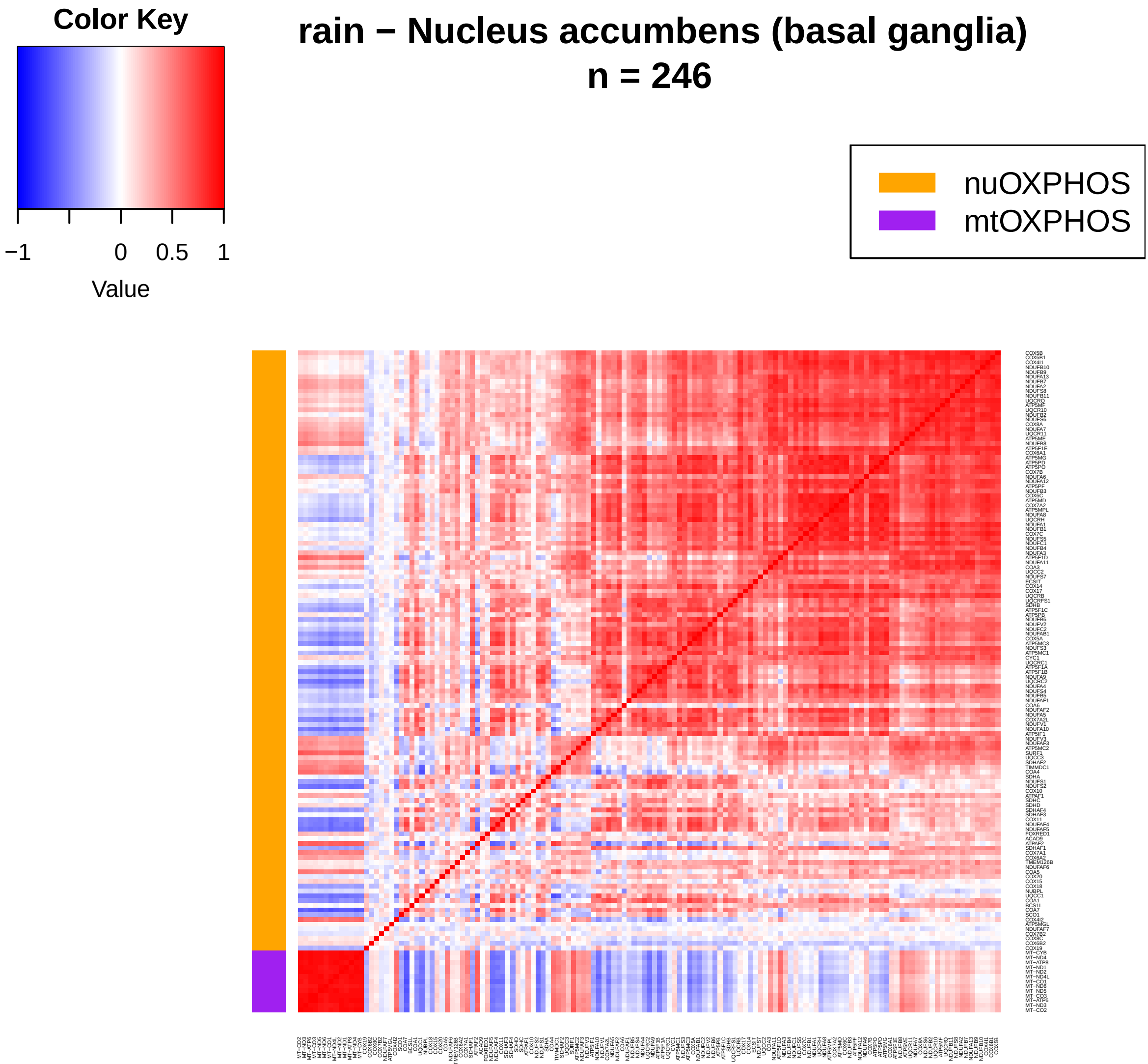
Color Key
−1
0
0.5
1
Value
rain − Nucleus accumbens (basal ganglia)
n = 246
nuOXPHOS
mtOXPHOS

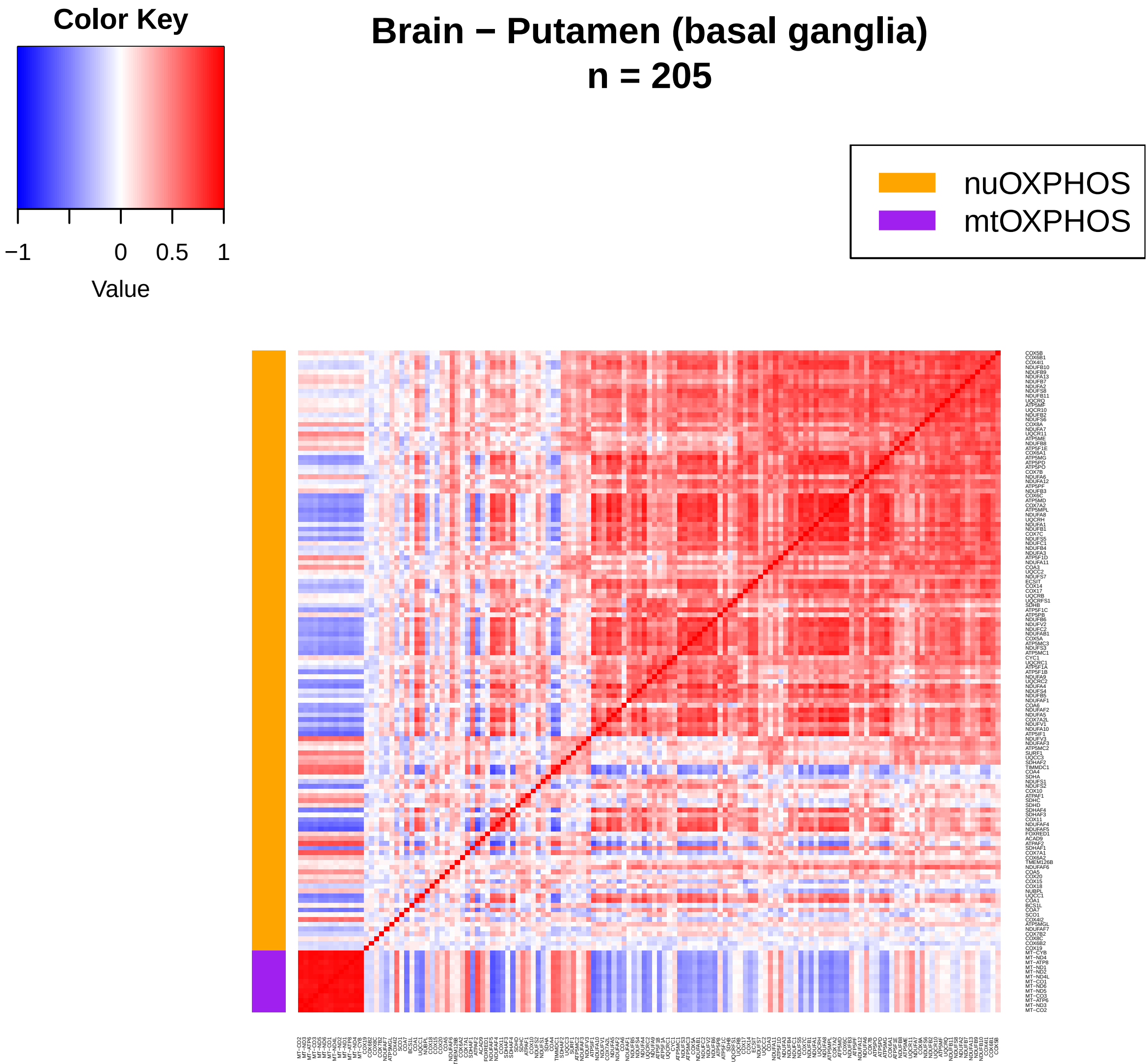
Color Key
−1
0
0.5
1
Value
Brain − Putamen (basal ganglia)
n = 205
nuOXPHOS
mtOXPHOS

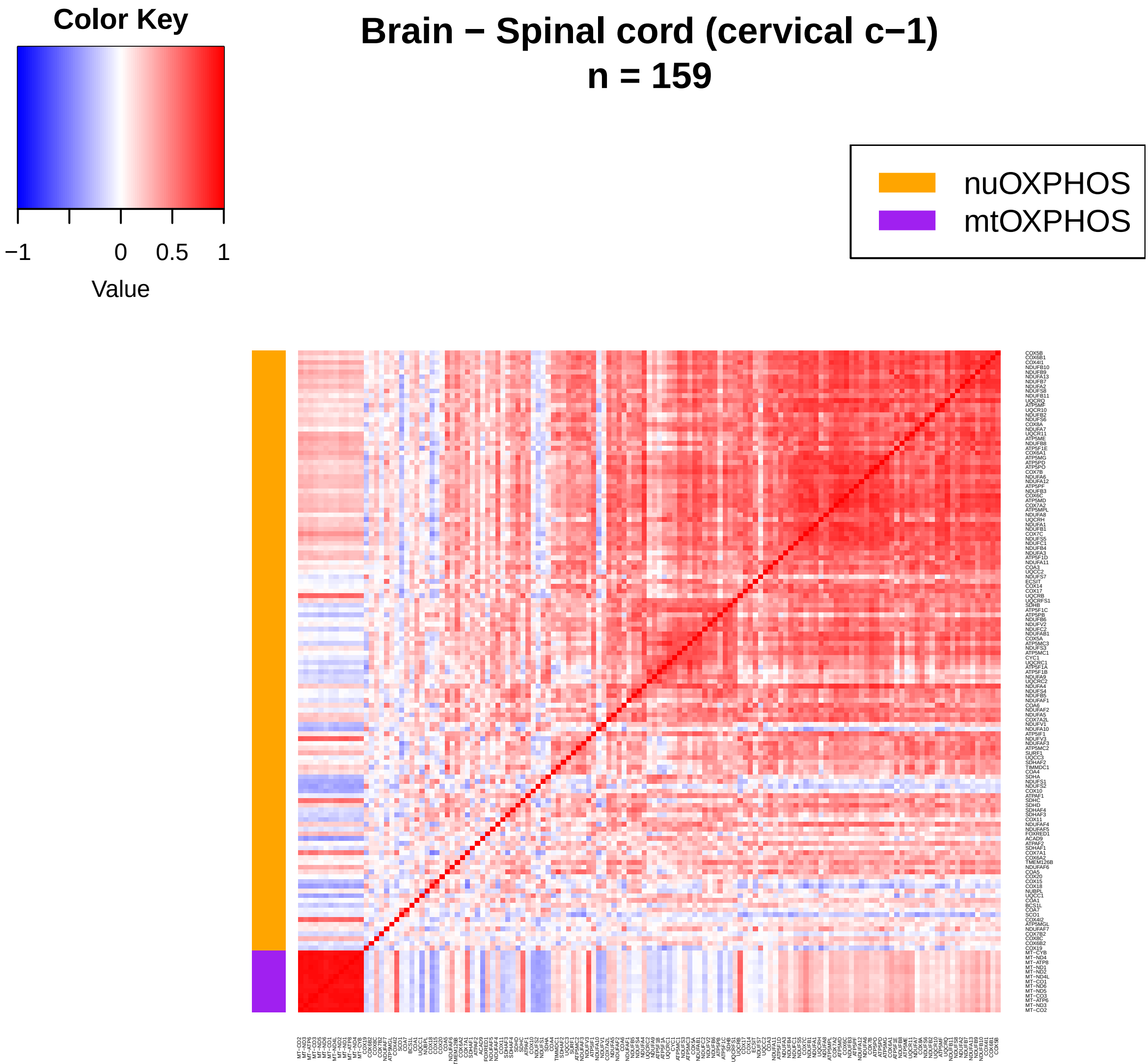
Brain − Spinal cord (cervical c−1)
n = 159
Color Key
−1
0
0.5
1
Value
nuOXPHOS
mtOXPHOS

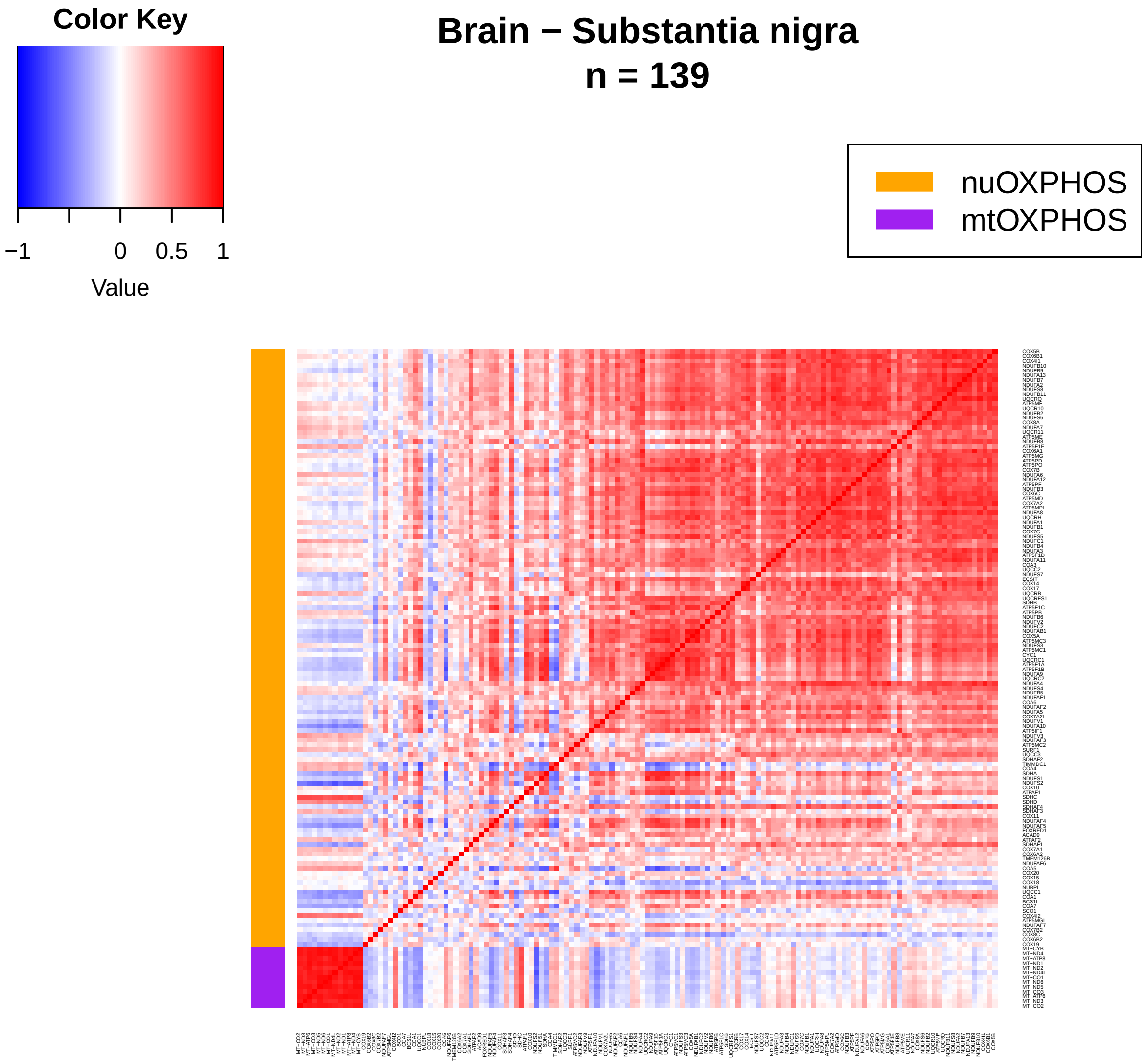
Color Key
−1
0
0.5
1
Value
Brain − Substantia nigra
n = 139
nuOXPHOS
mtOXPHOS

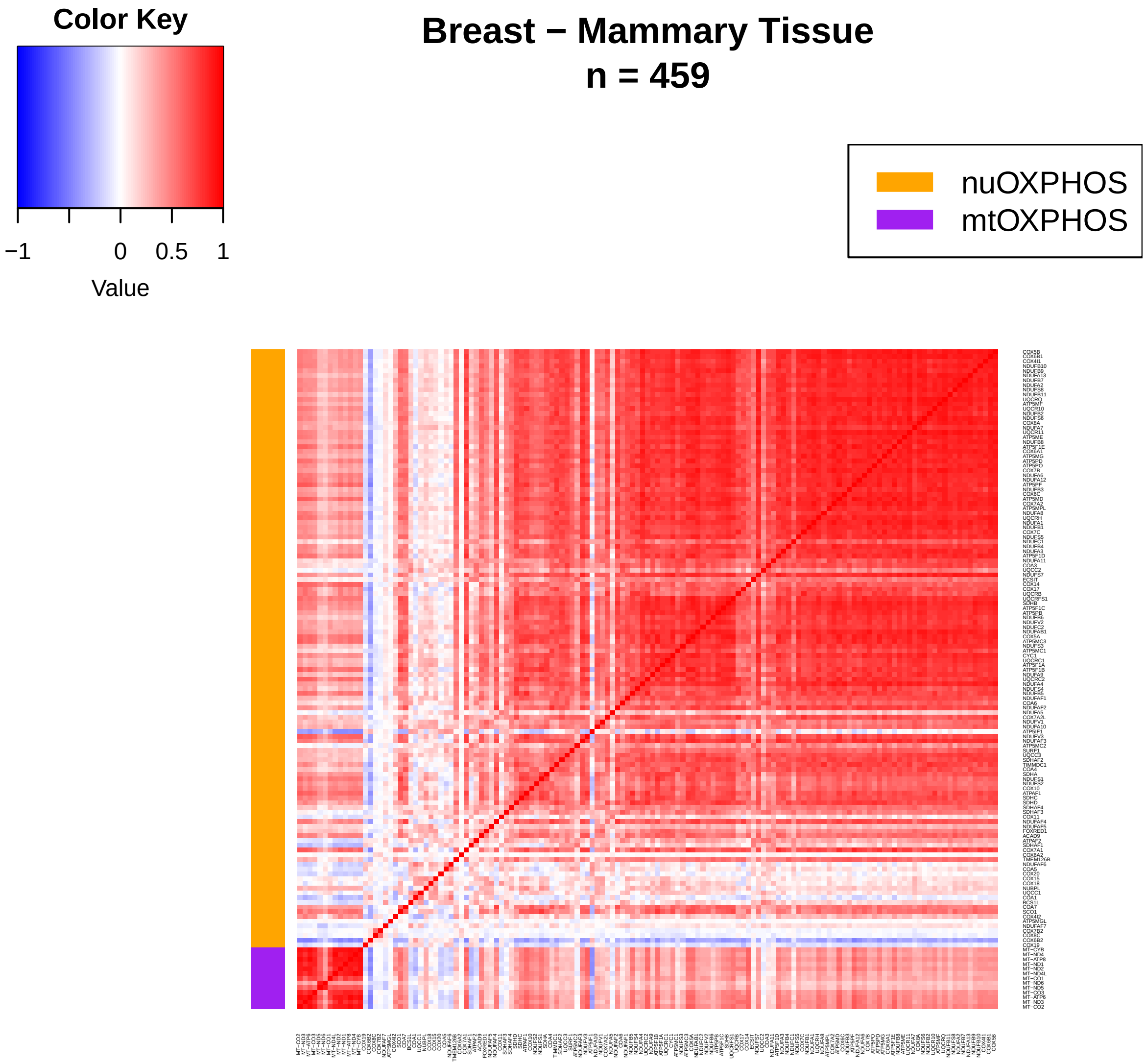
Color Key
−1
0
0.5
1
Value
Breast − Mammary Tissue
n = 459
nuOXPHOS
mtOXPHOS

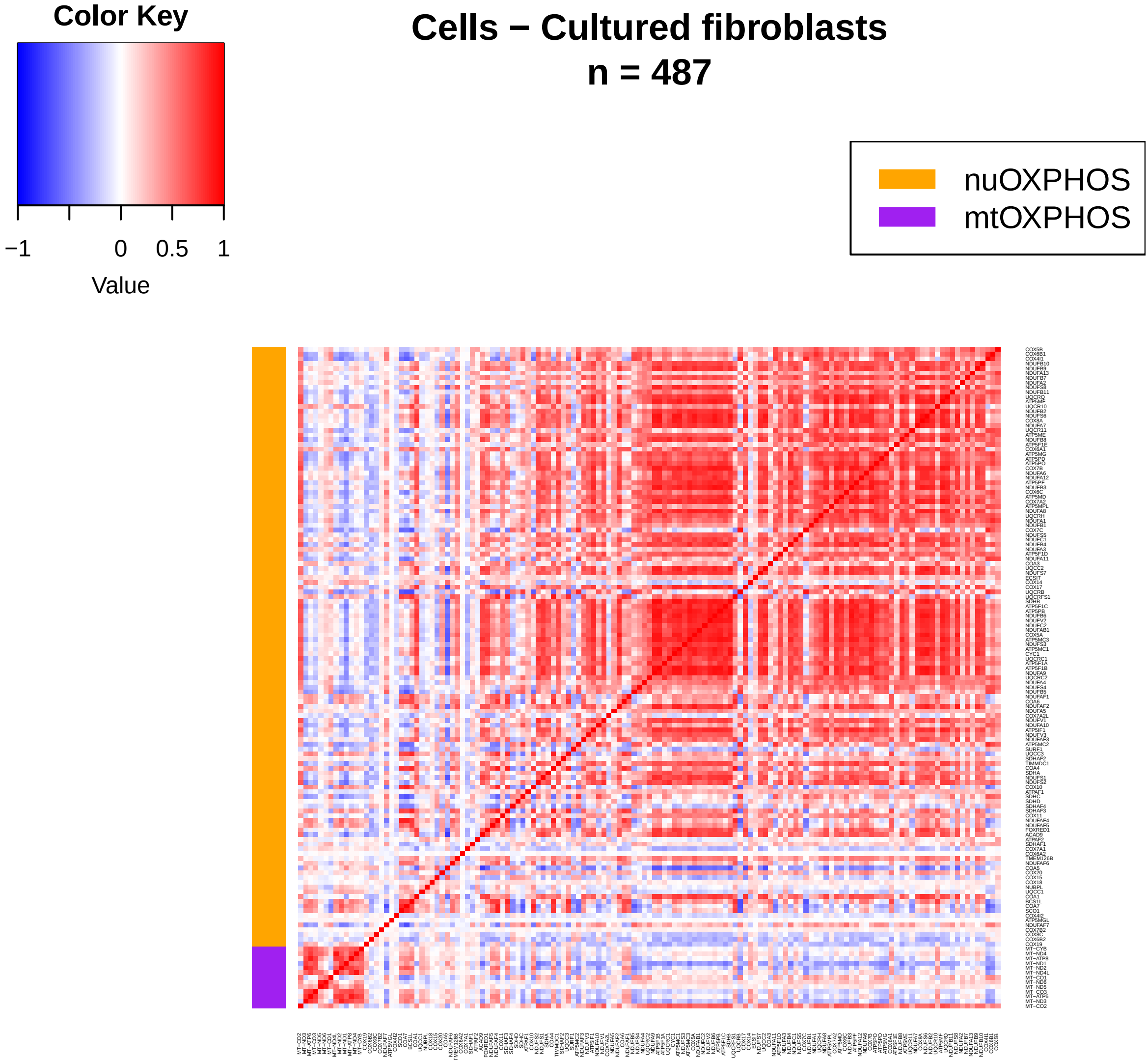
Color Key
−1
0
0.5
1
Value
Cells − Cultured fibroblasts
n = 487
nuOXPHOS
mtOXPHOS

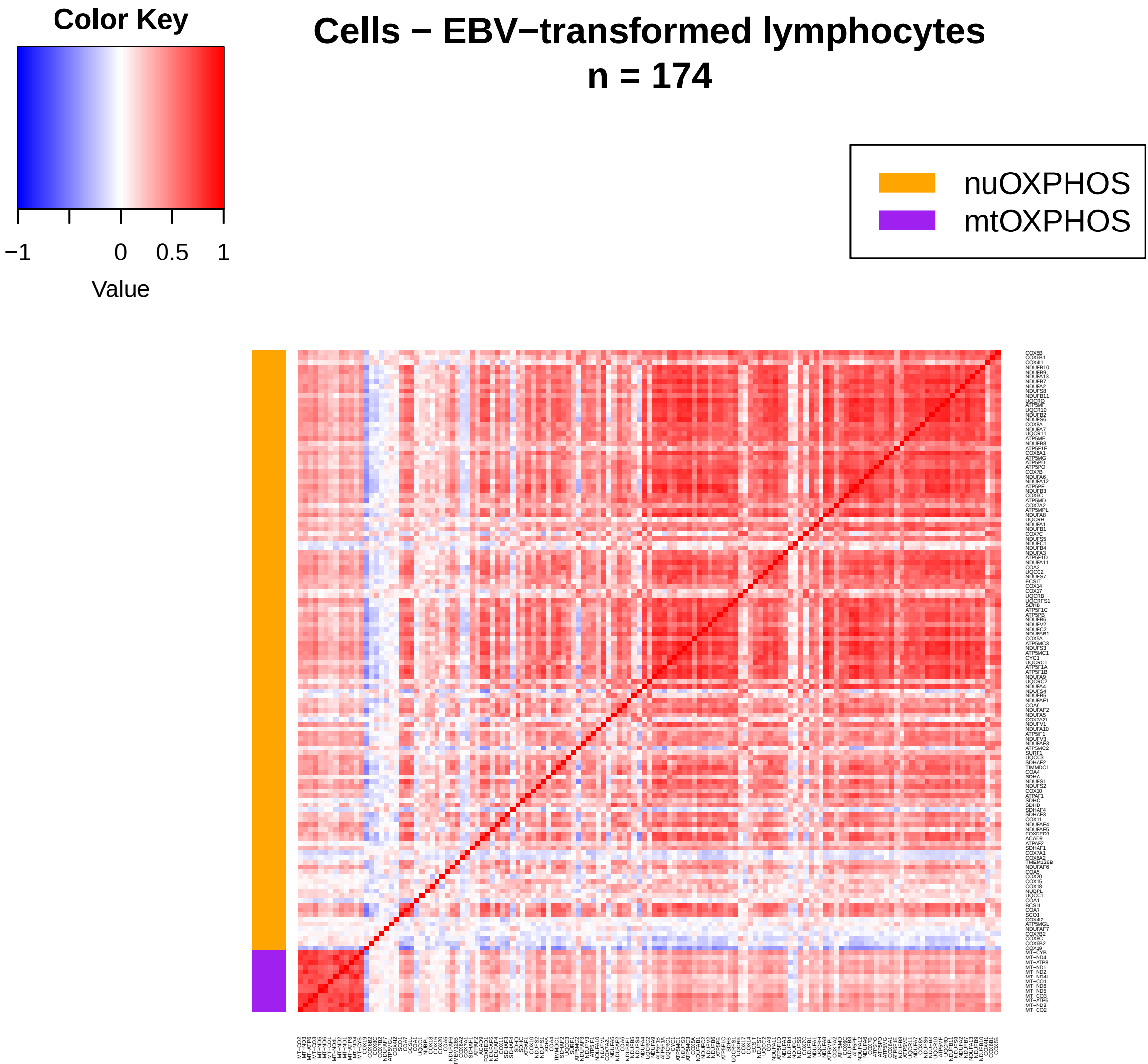
Color Key
−1
0
0.5
1
Value
Cells − EBV−transformed lymphocytes
n = 174
nuOXPHOS
mtOXPHOS

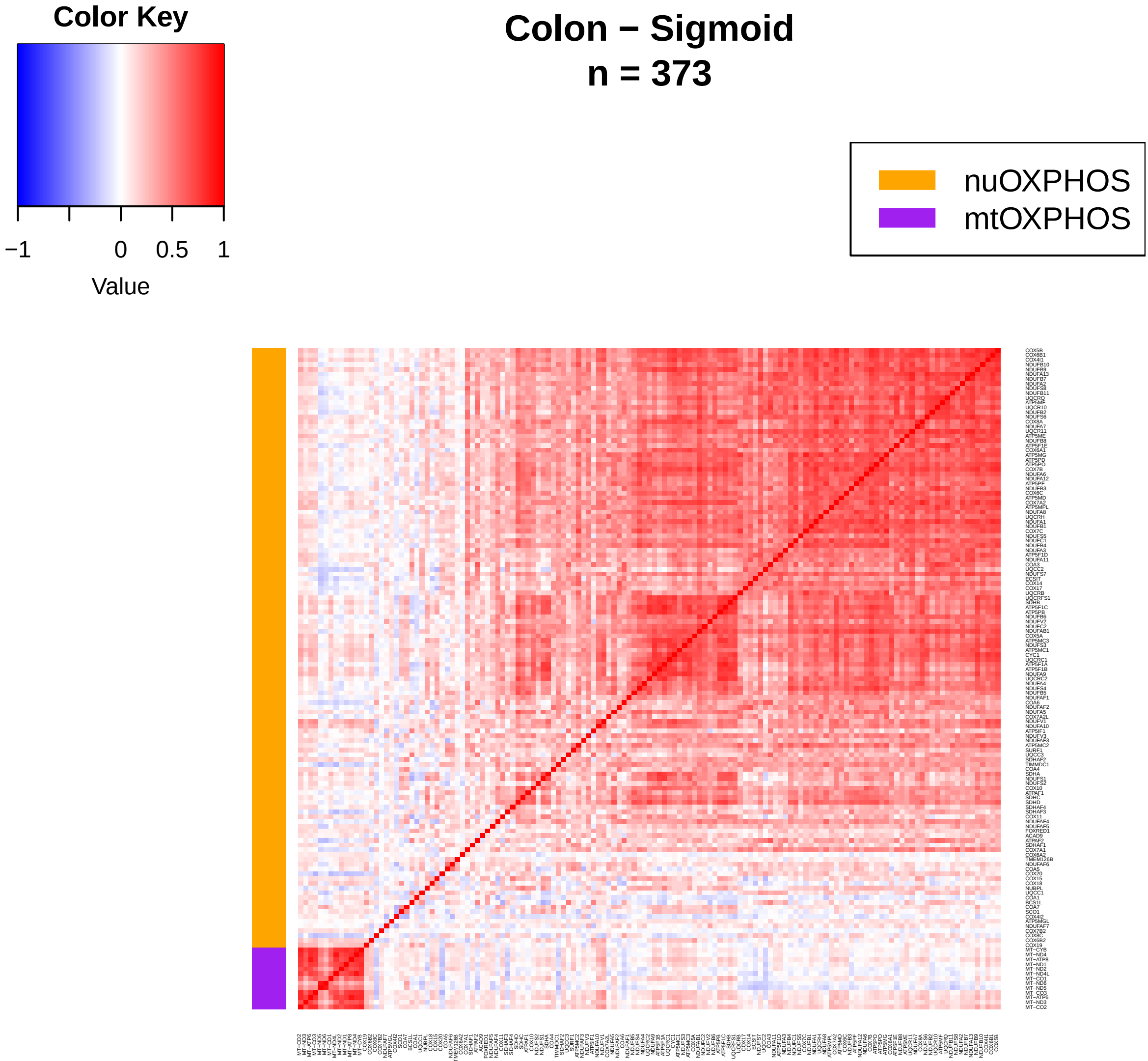
Color Key
−1
0
0.5
1
Value
Colon − Sigmoid
n = 373
nuOXPHOS
mtOXPHOS

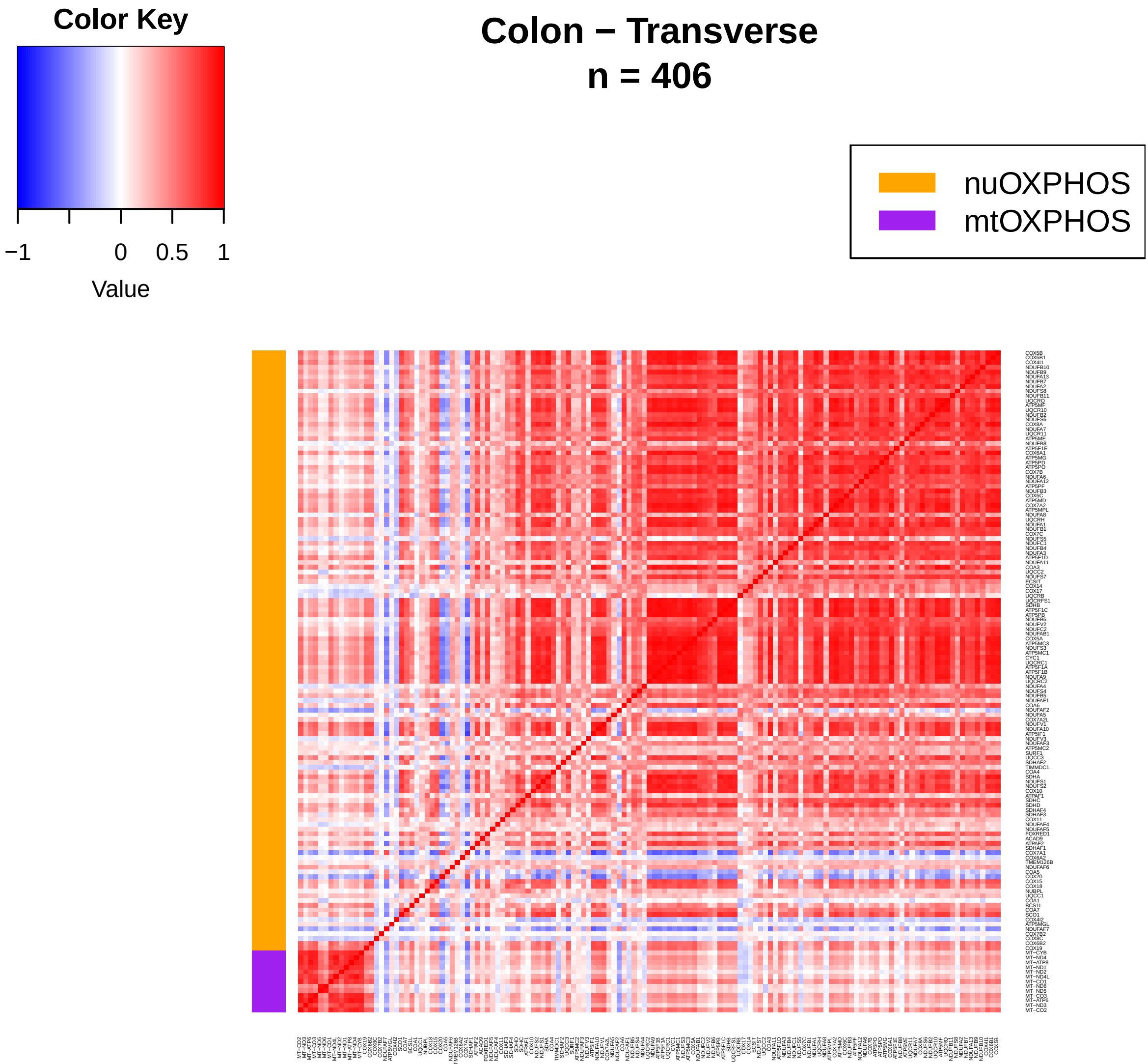
Color Key
−1
0
0.5
1
Value
Colon − Transverse
n = 406
nuOXPHOS
mtOXPHOS

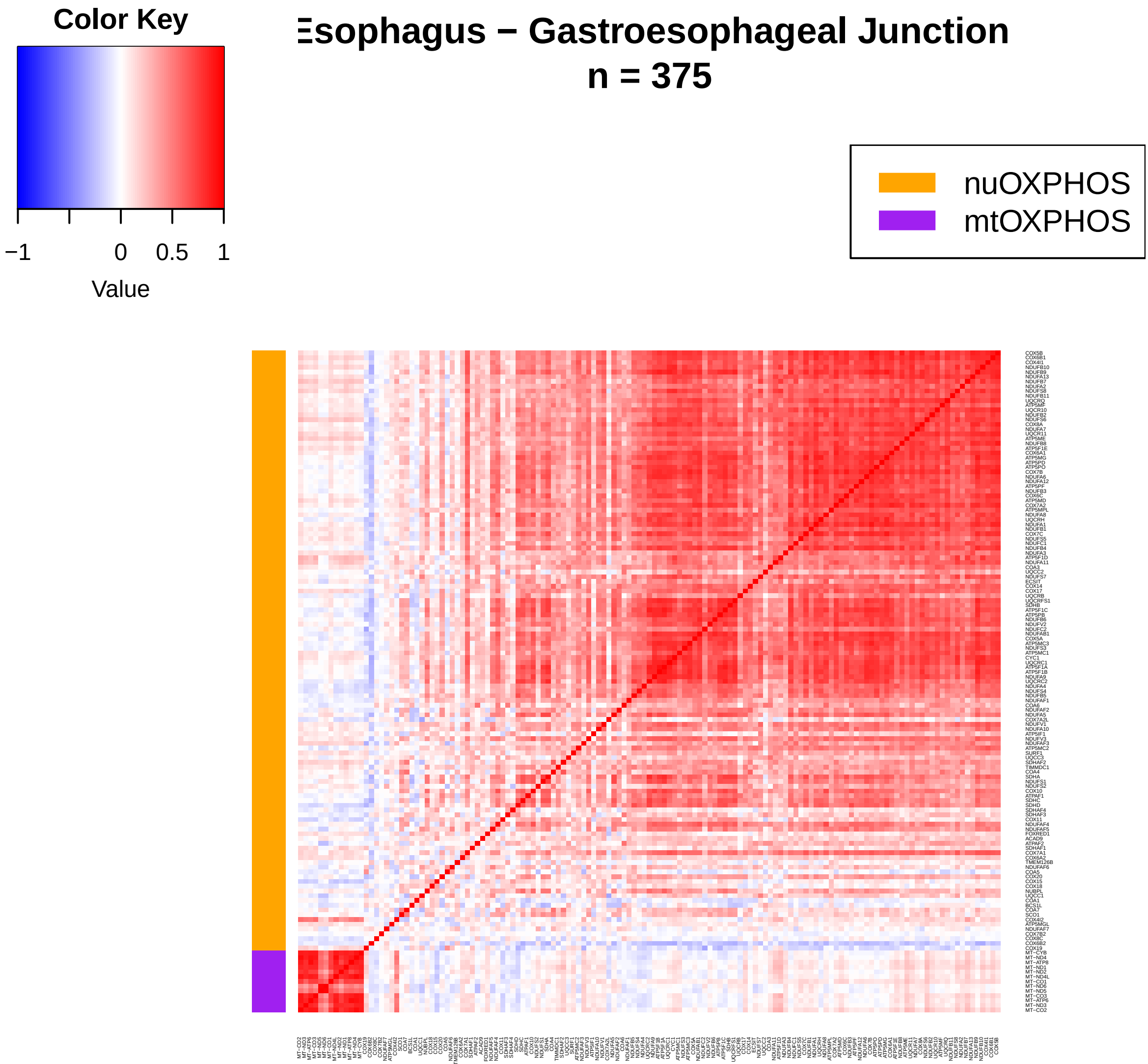
Color Key
−1
0
0.5
1
Value
Esophagus − Gastroesophageal Junction
n = 375
nuOXPHOS
mtOXPHOS

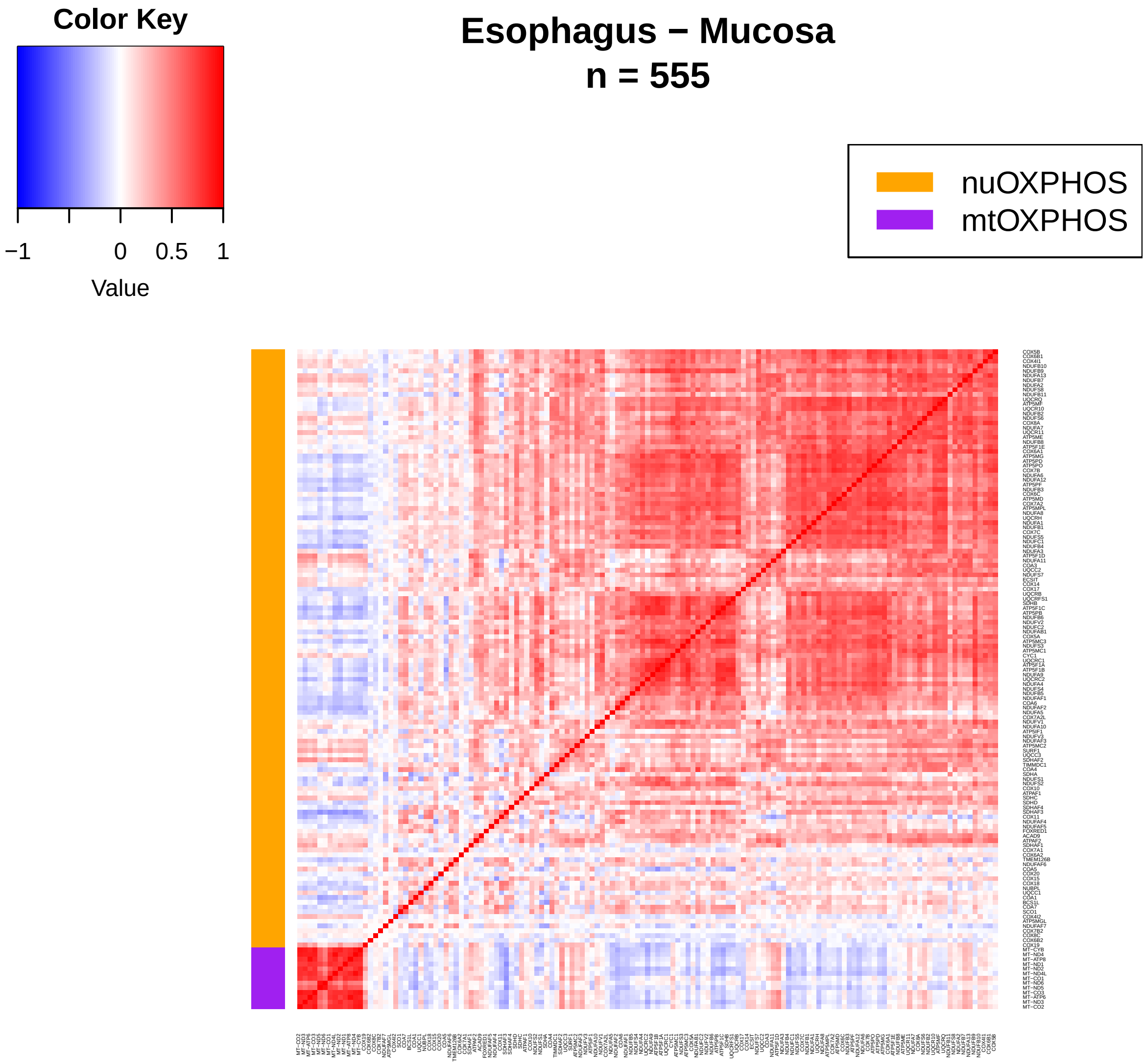
Color Key
−1
0
0.5
1
Value
Esophagus − Mucosa
n = 555
nuOXPHOS
mtOXPHOS

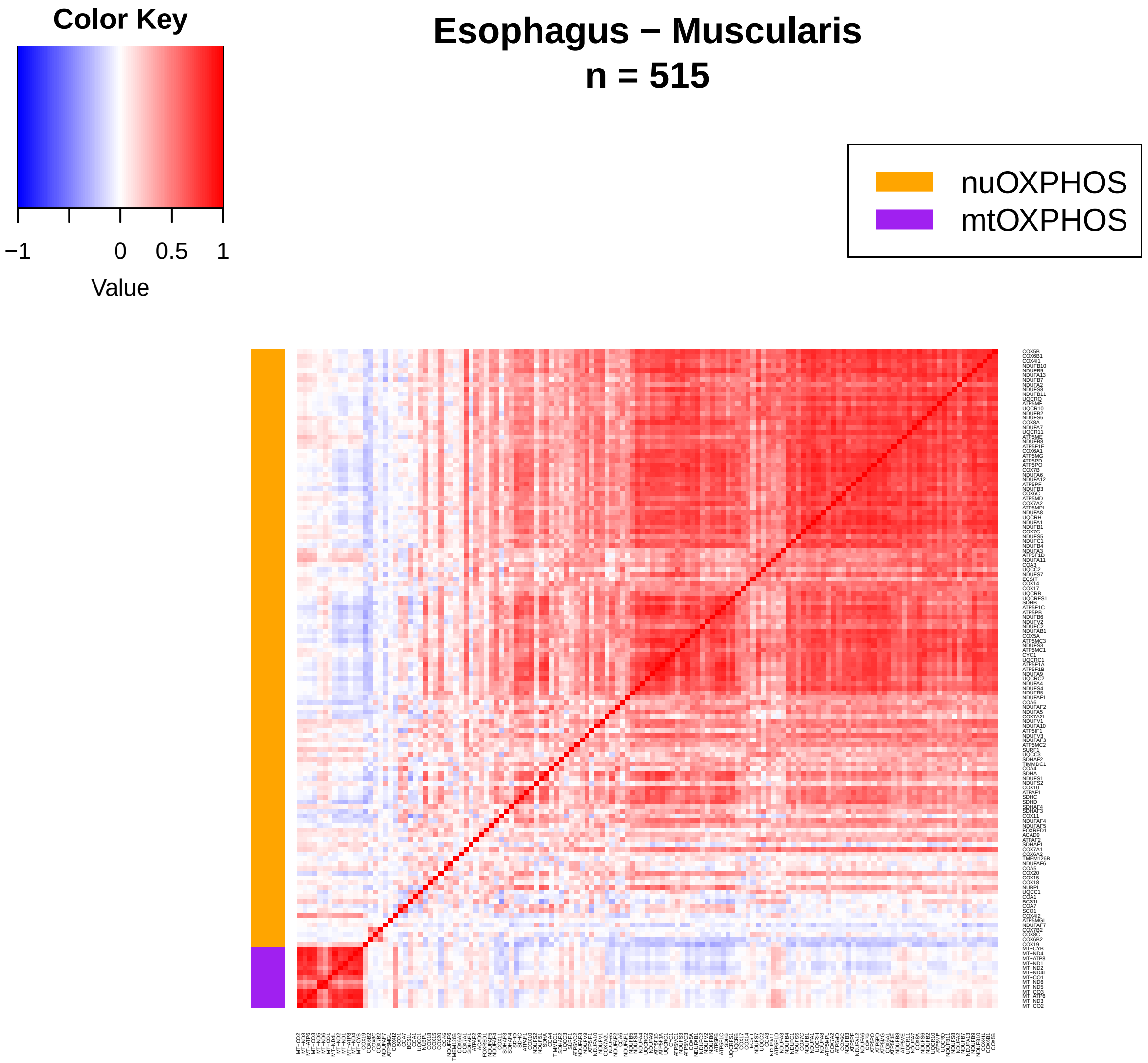
Esophagus − Muscularis
n = 515
Color Key
−1
0
0.5
1
Value
nuOXPHOS
mtOXPHOS

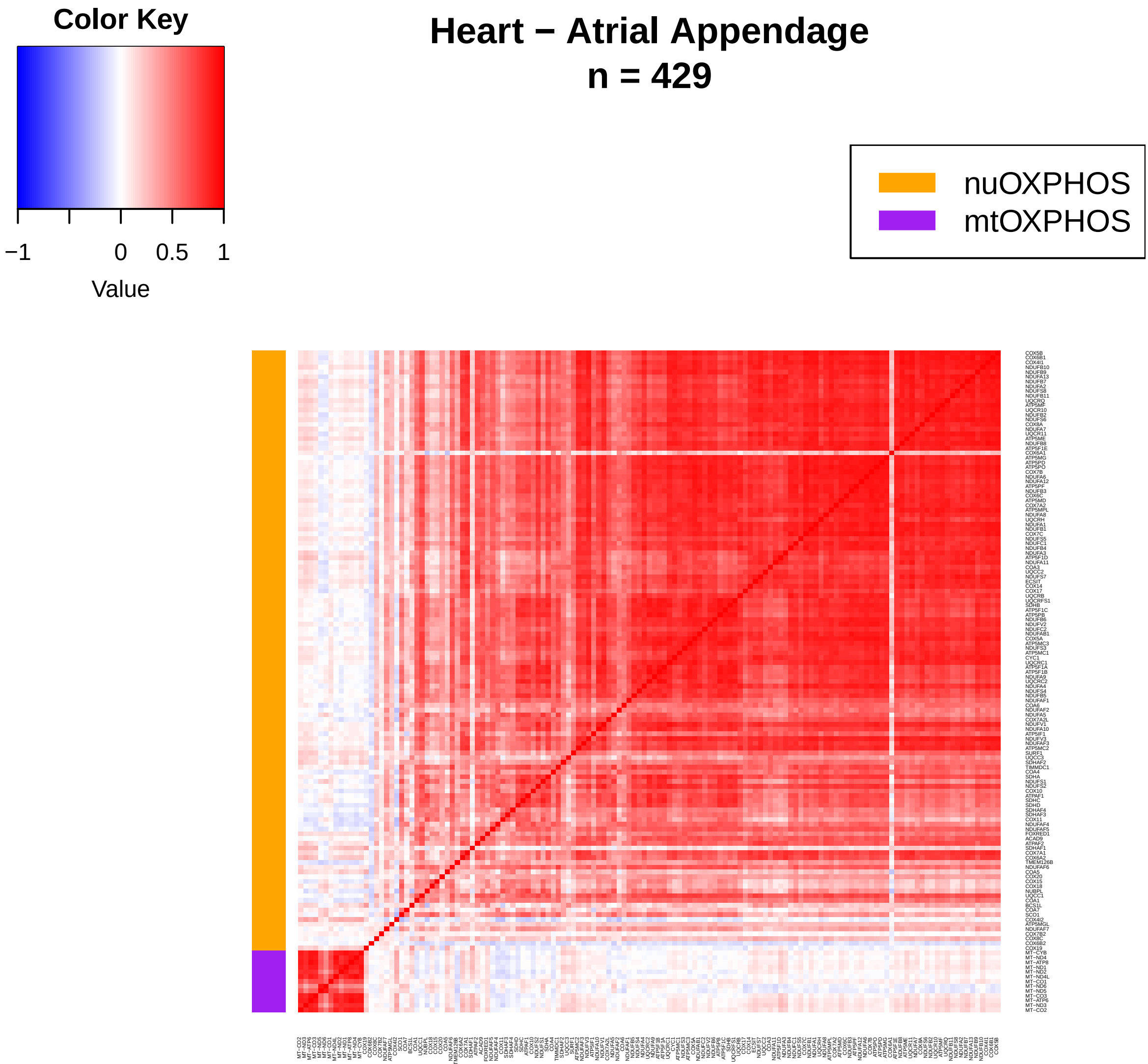
Color Key
−1
0
0.5
1
Value
Heart − Atrial Appendage
n = 429
nuOXPHOS
mtOXPHOS

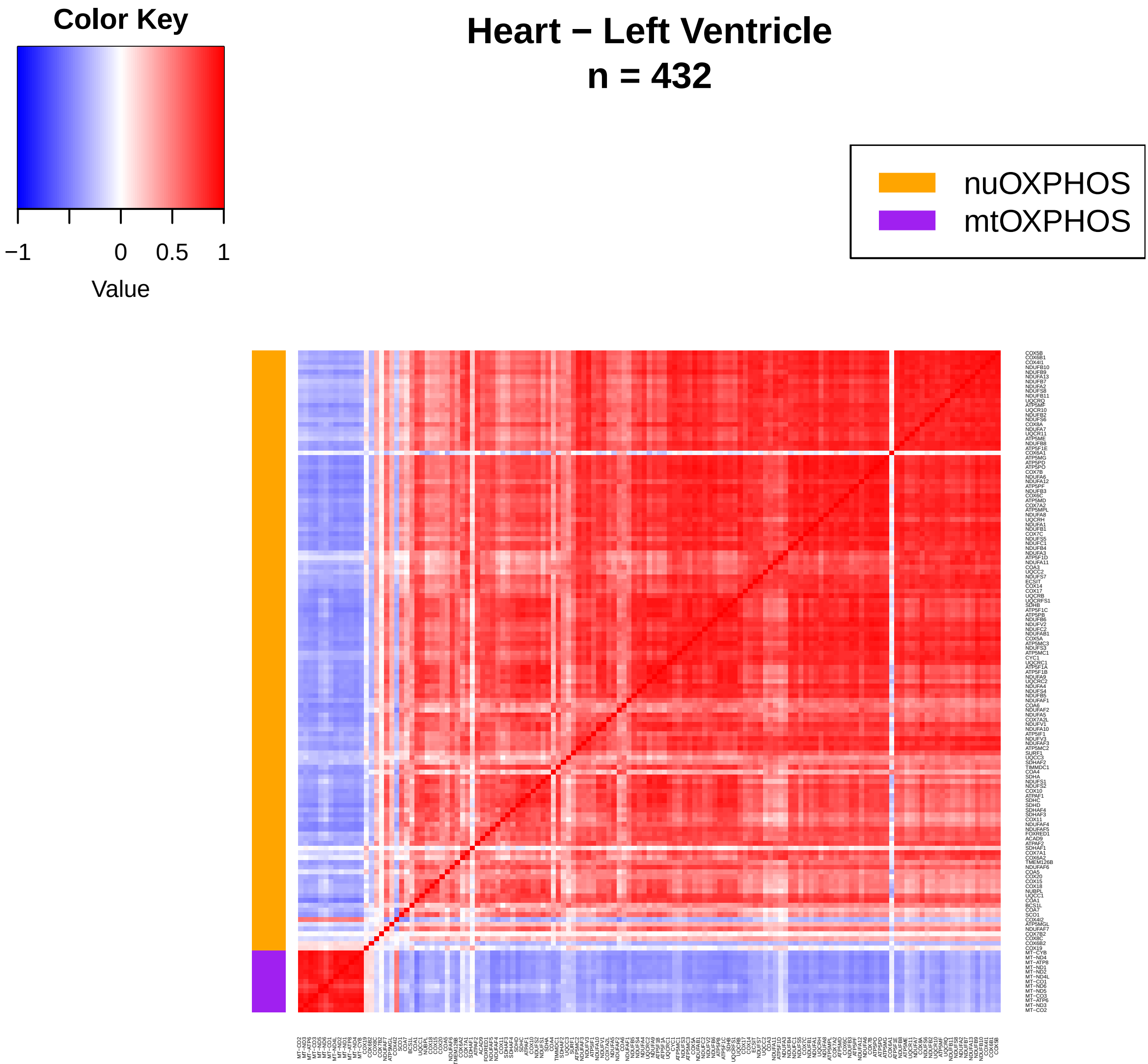
Color Key
−1
0
0.5
1
Value
Heart − Left Ventricle
n = 432
nuOXPHOS
mtOXPHOS

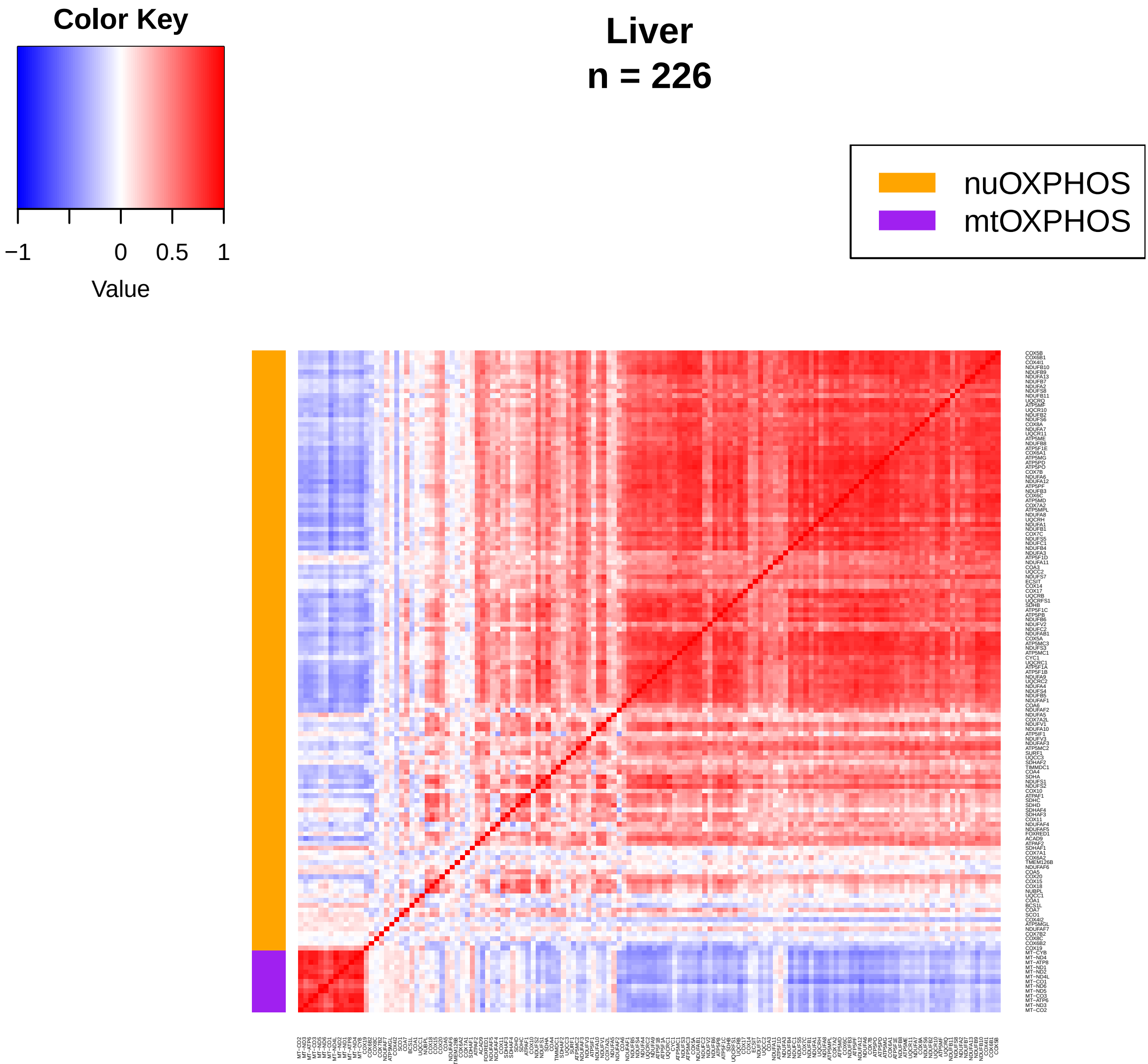
Color Key
−1
0
0.5
1
Value
Liver
n = 226
nuOXPHOS
mtOXPHOS

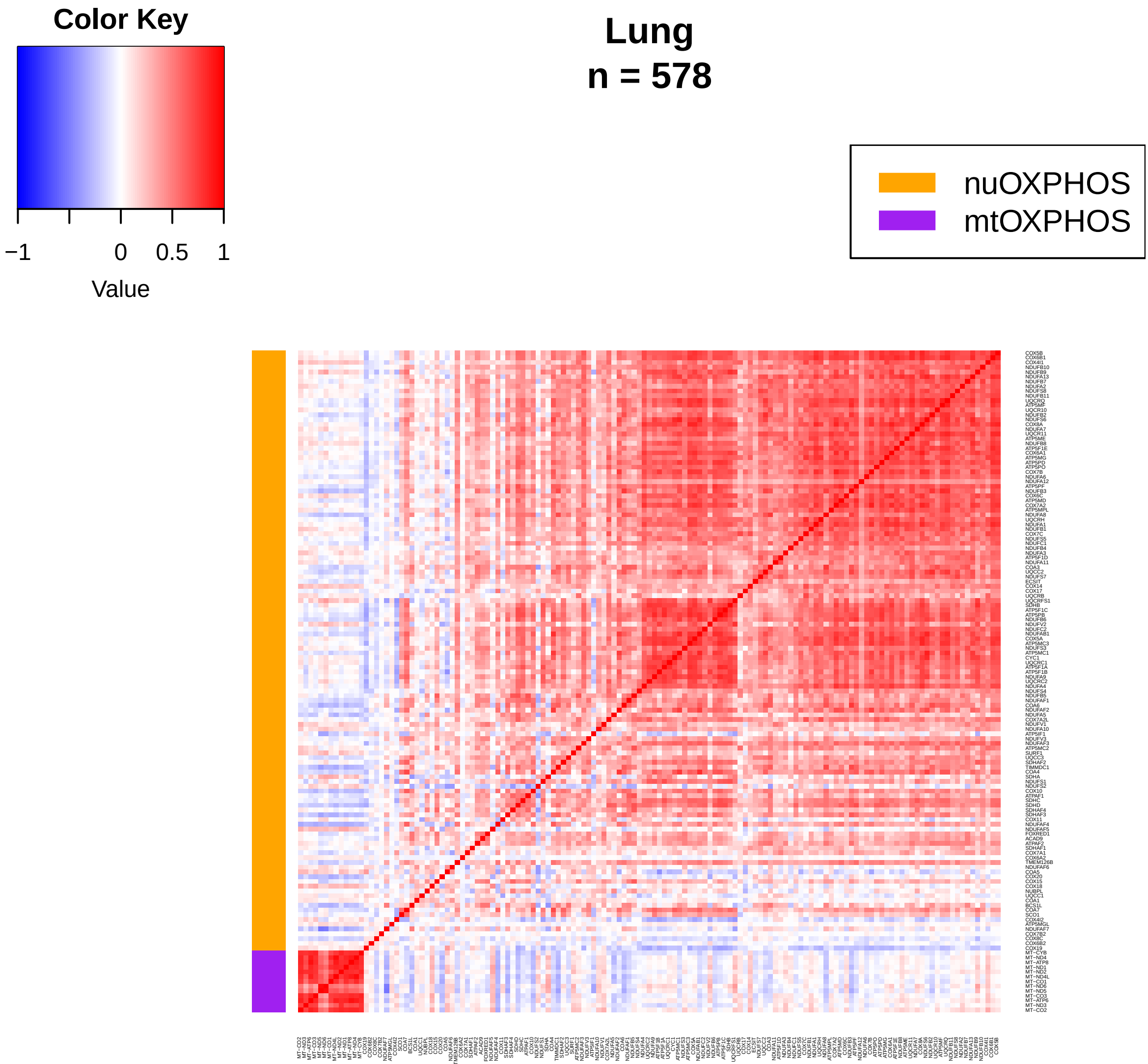
Color Key
−1
0
0.5
1
Value
Lung
n = 578
nuOXPHOS
mtOXPHOS

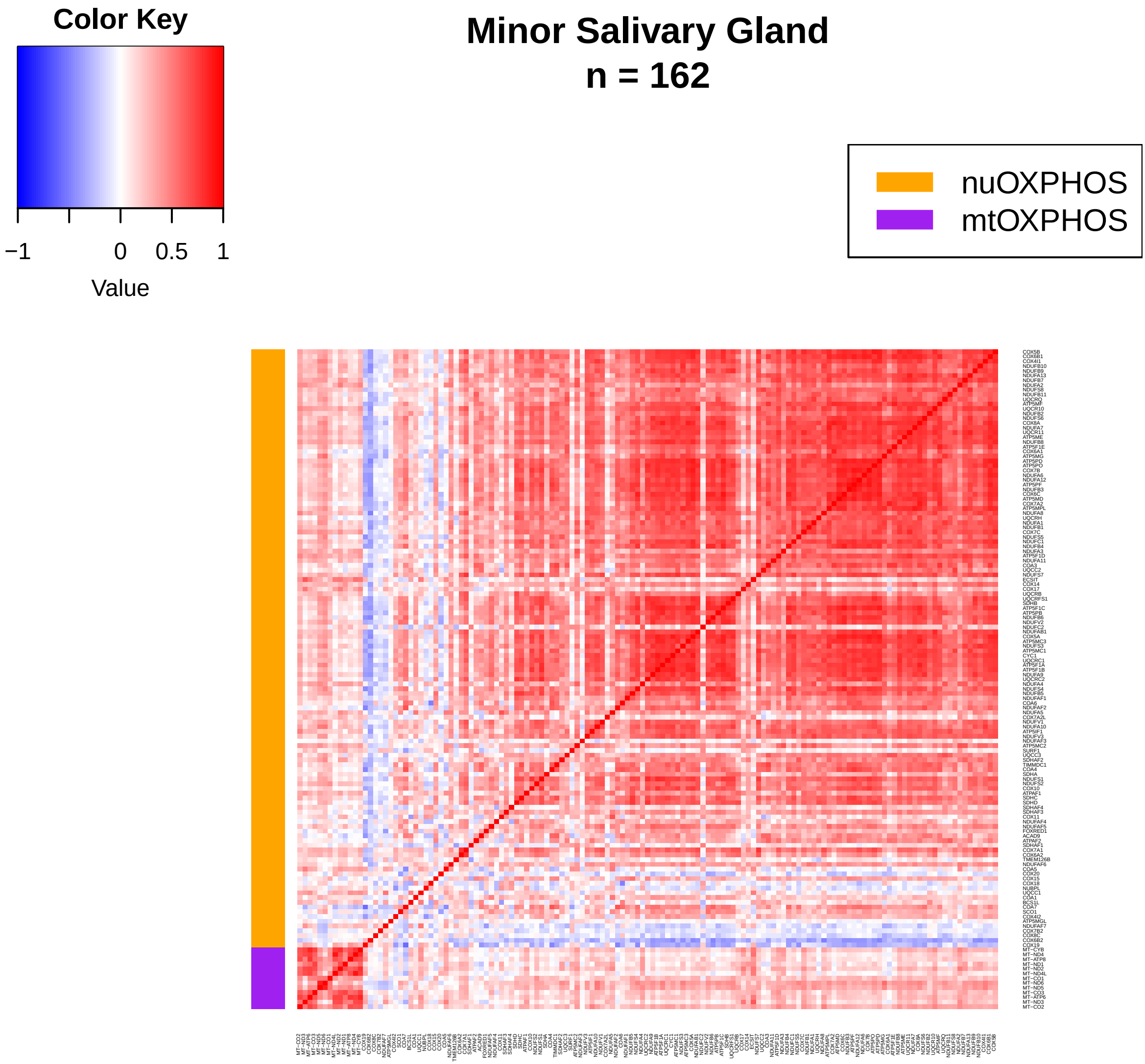
Color Key
−1
0
0.5
1
Value
Minor Salivary Gland
n = 162
nuOXPHOS
mtOXPHOS

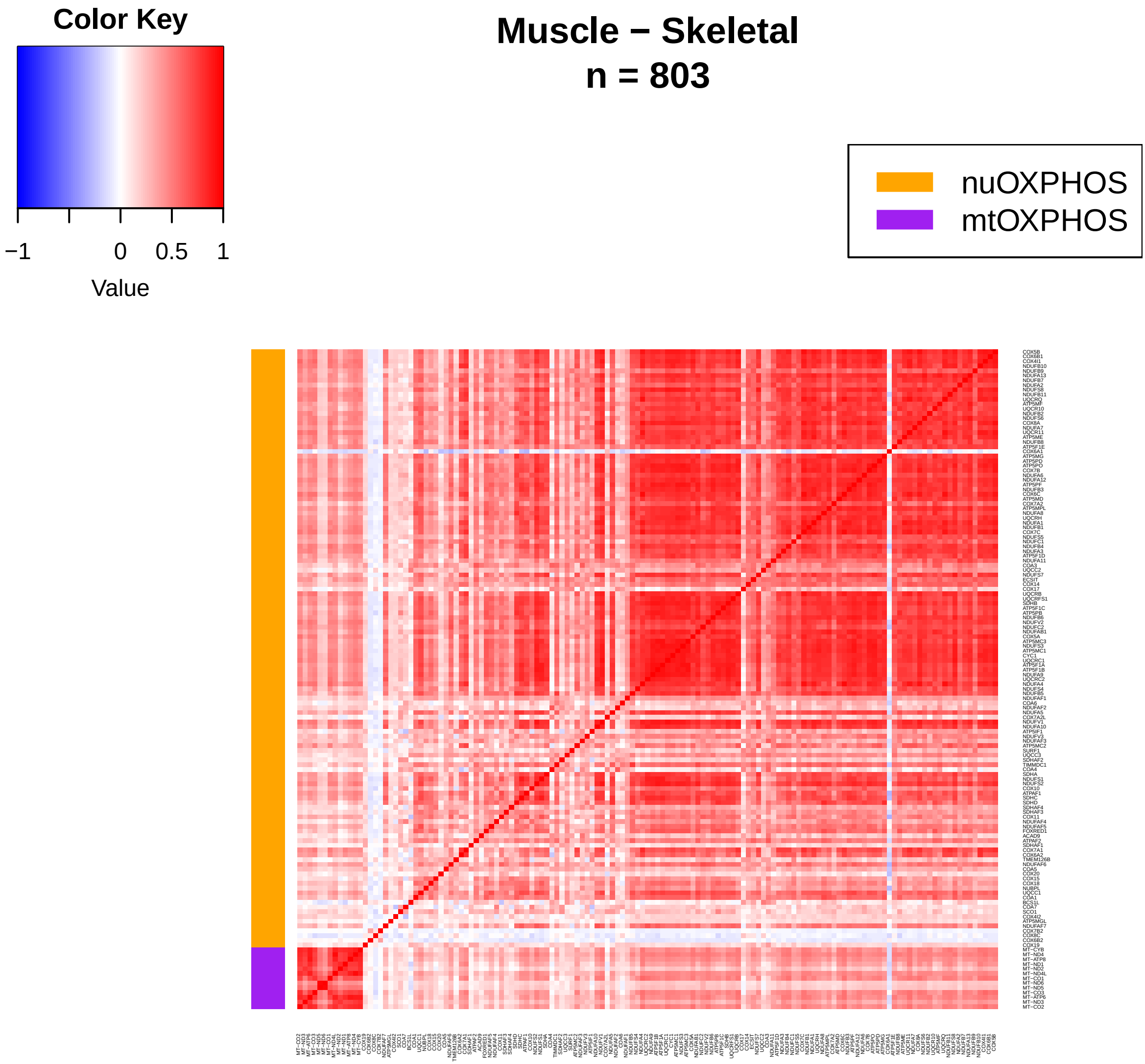
Color Key
−1
0
0.5
1
Value
Muscle − Skeletal
n = 803
nuOXPHOS
mtOXPHOS

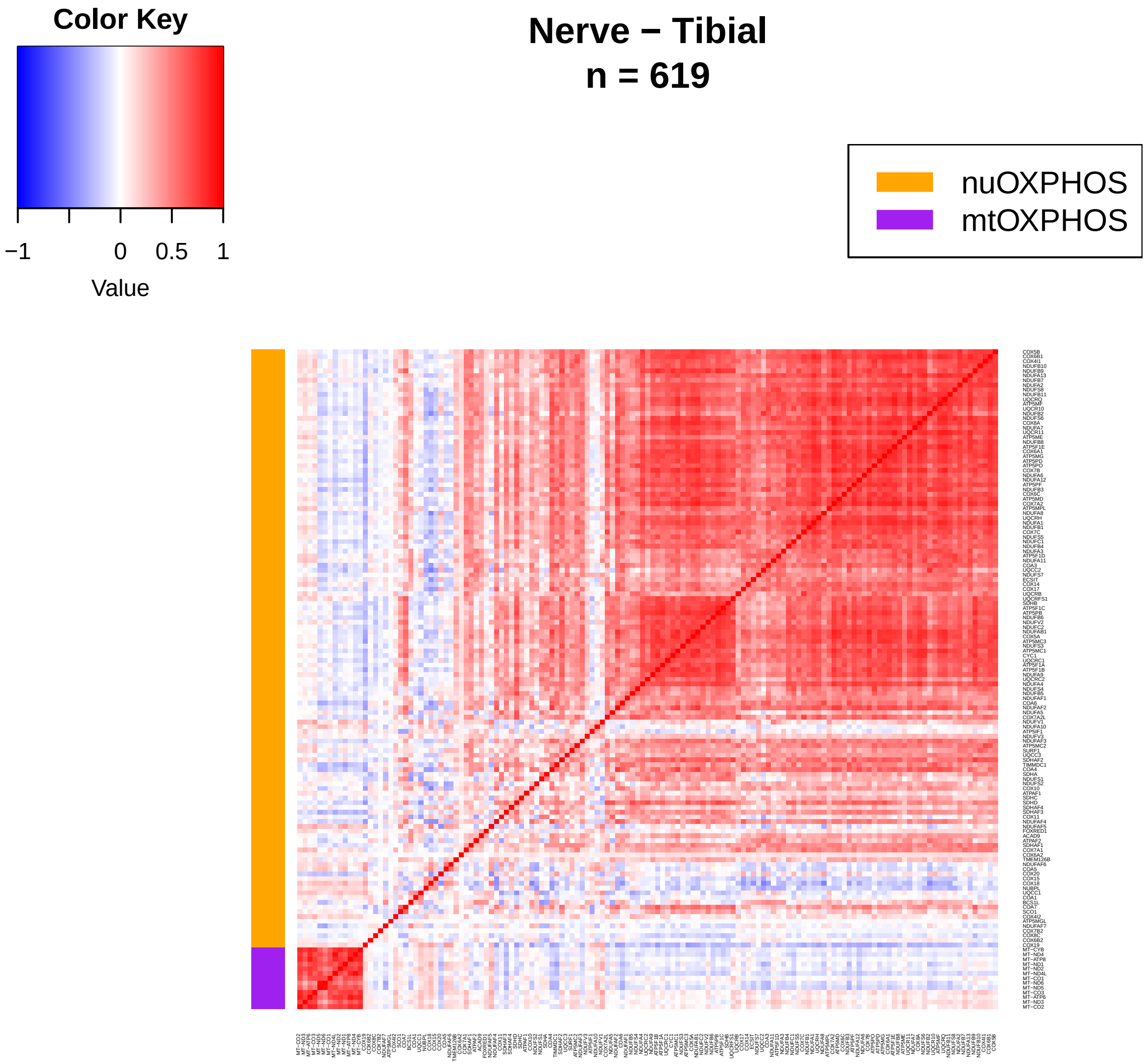
Color Key
Value
−1
0
0.5
1
Nerve − Tibial
n = 619
nuOXPHOS
mtOXPHOS

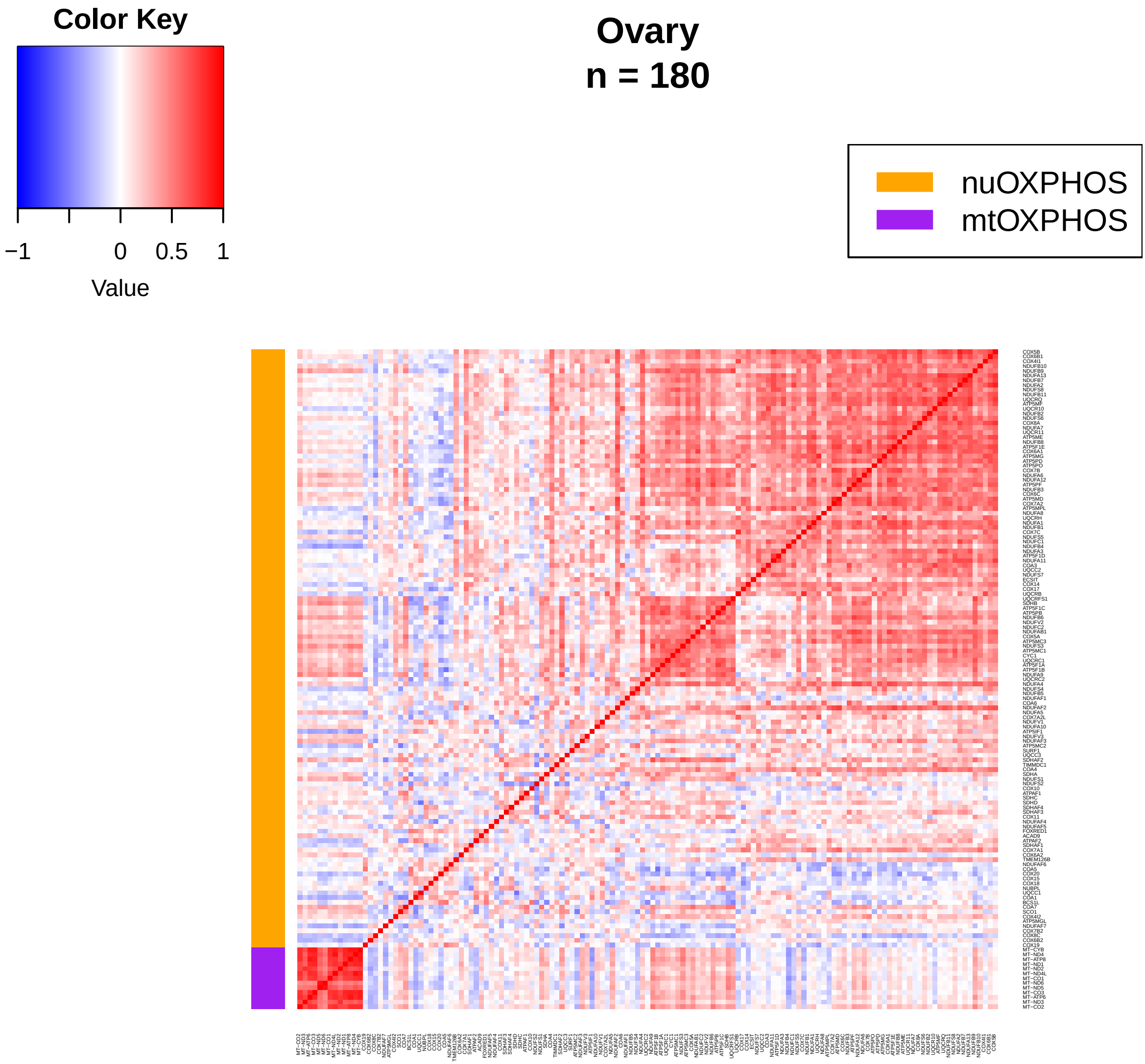
Ovary
n = 180
Color Key
−1
0
0.5
1
Value
nuOXPHOS
mtOXPHOS

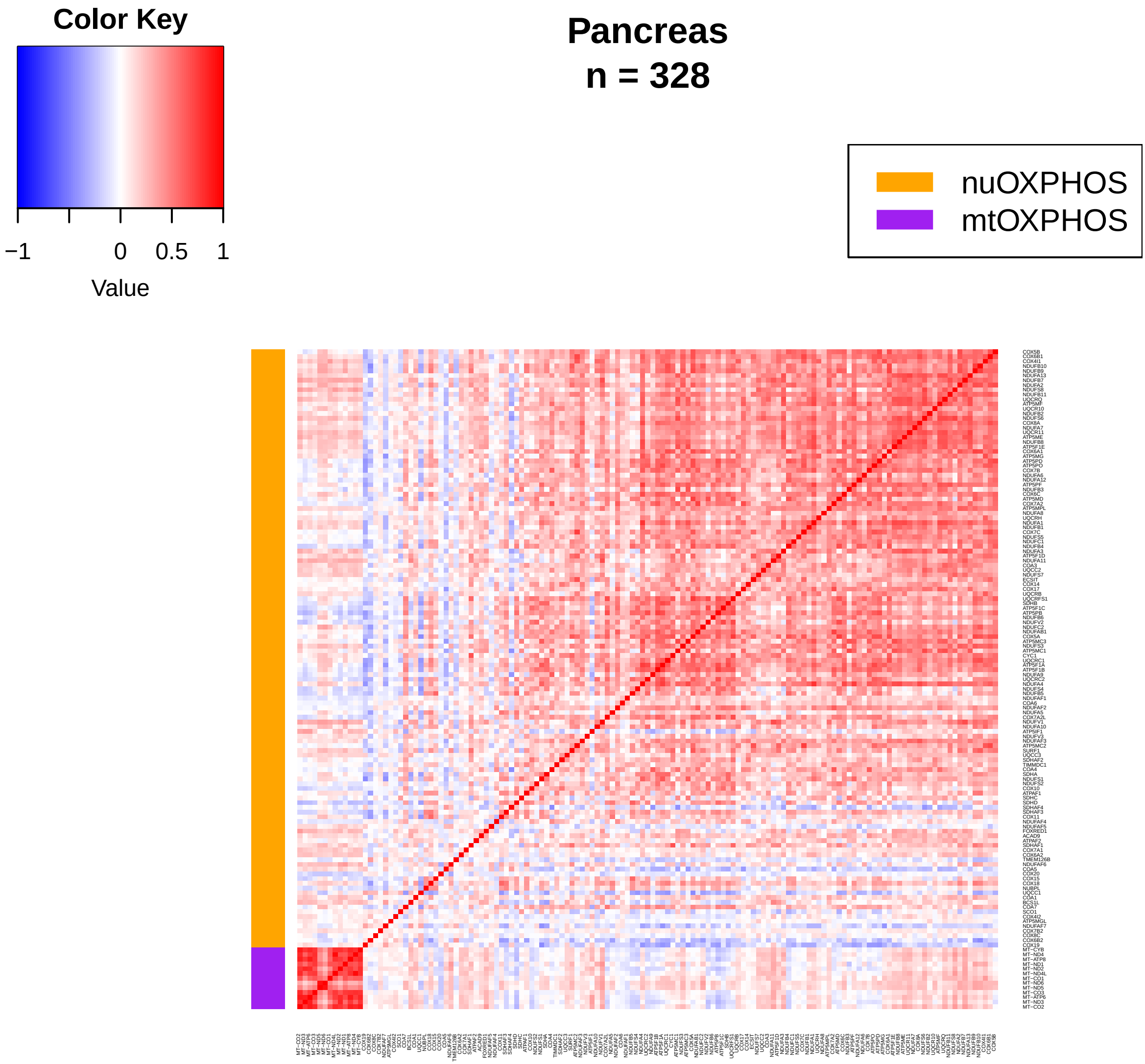
Color Key
−1
0
0.5
1
Value
Pancreas
n = 328
nuOXPHOS
mtOXPHOS

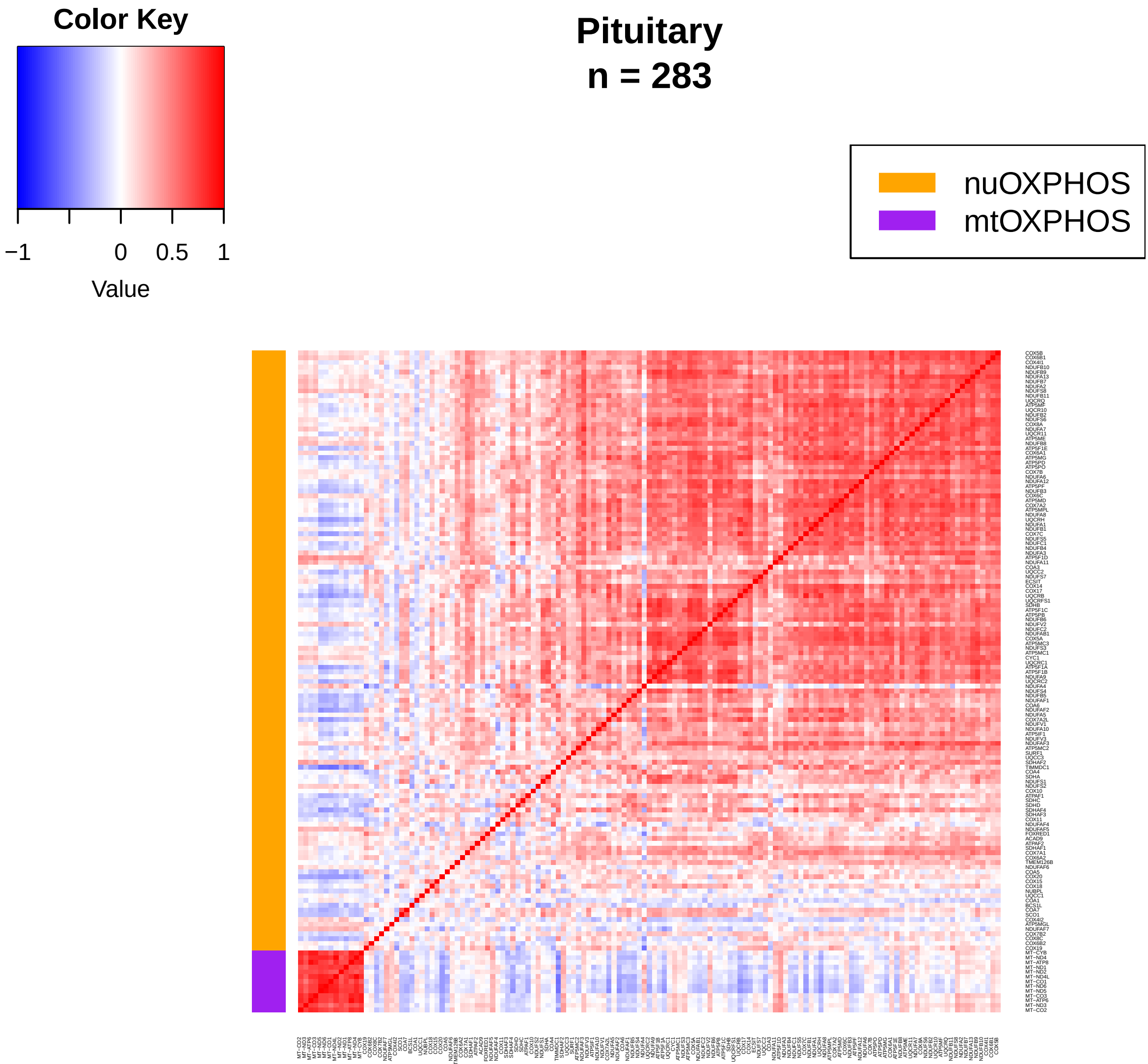
Color Key
−1
0
0.5
1
Value
Pituitary
n = 283
nuOXPHOS
mtOXPHOS

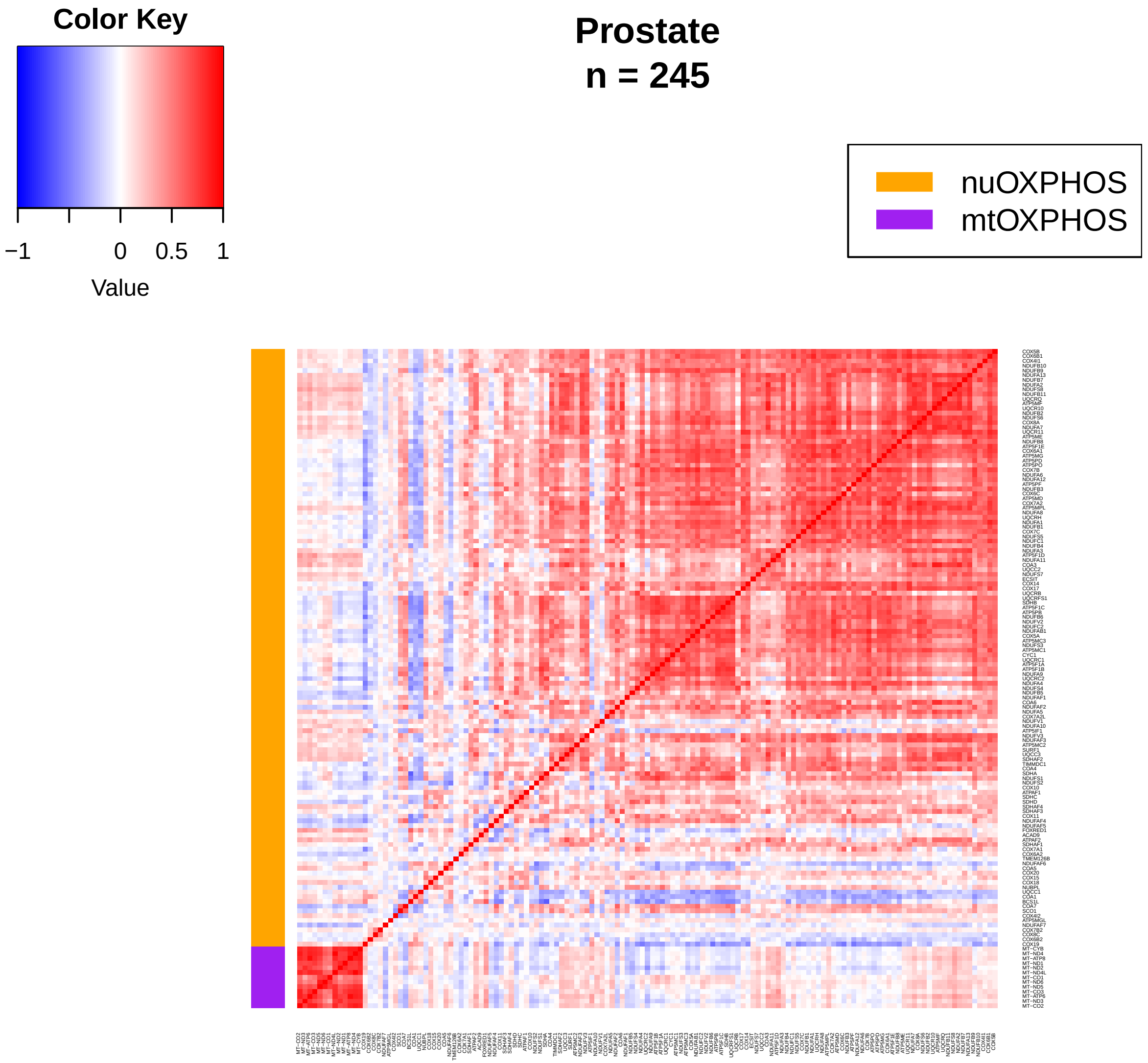
Color Key
−1
0
0.5
1
Value
Prostate
n = 245
nuOXPHOS
mtOXPHOS

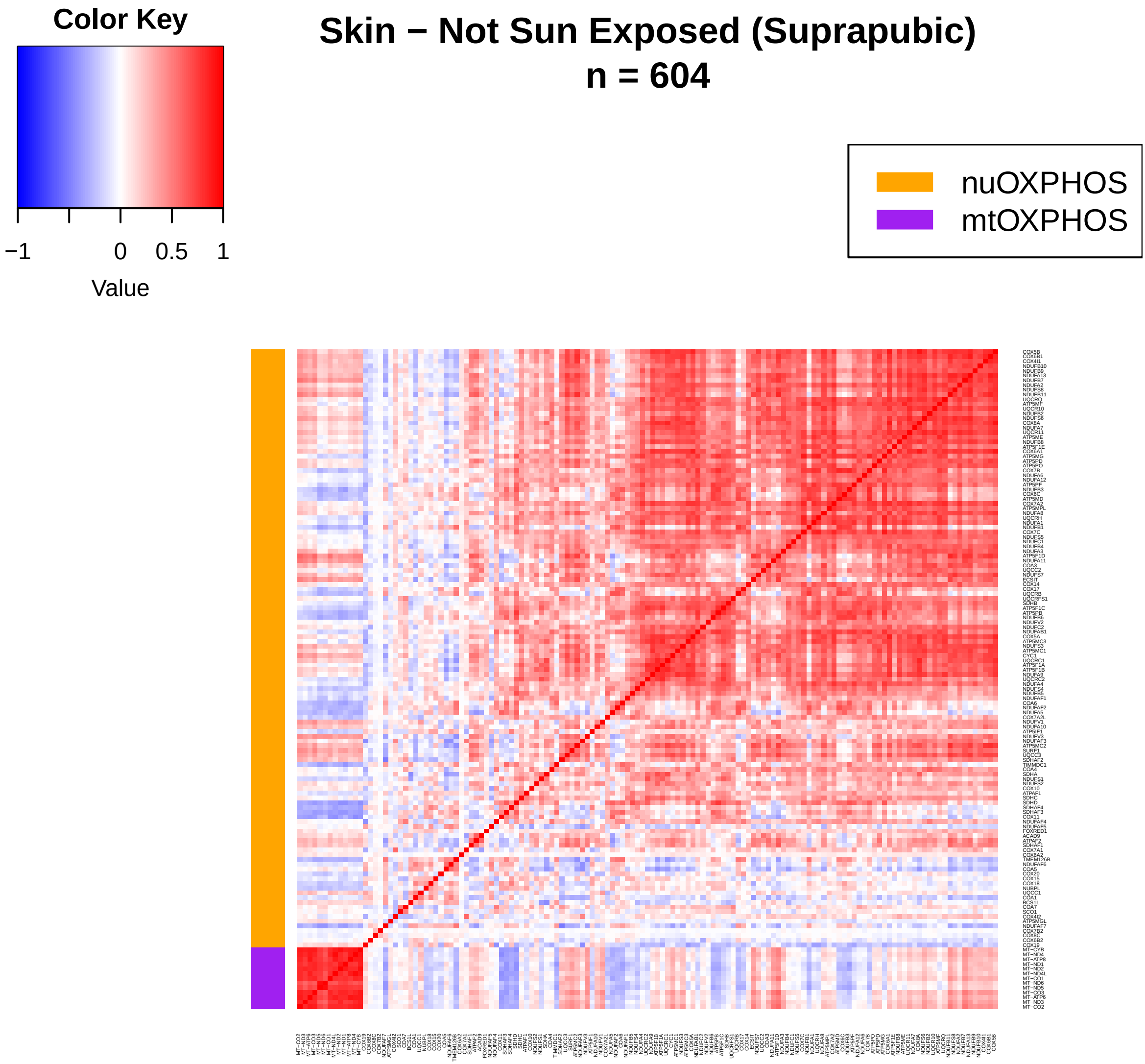
Skin − Not Sun Exposed (Suprapubic)
n = 604
Color Key
−1
0
0.5
1
Value
nuOXPHOS
mtOXPHOS

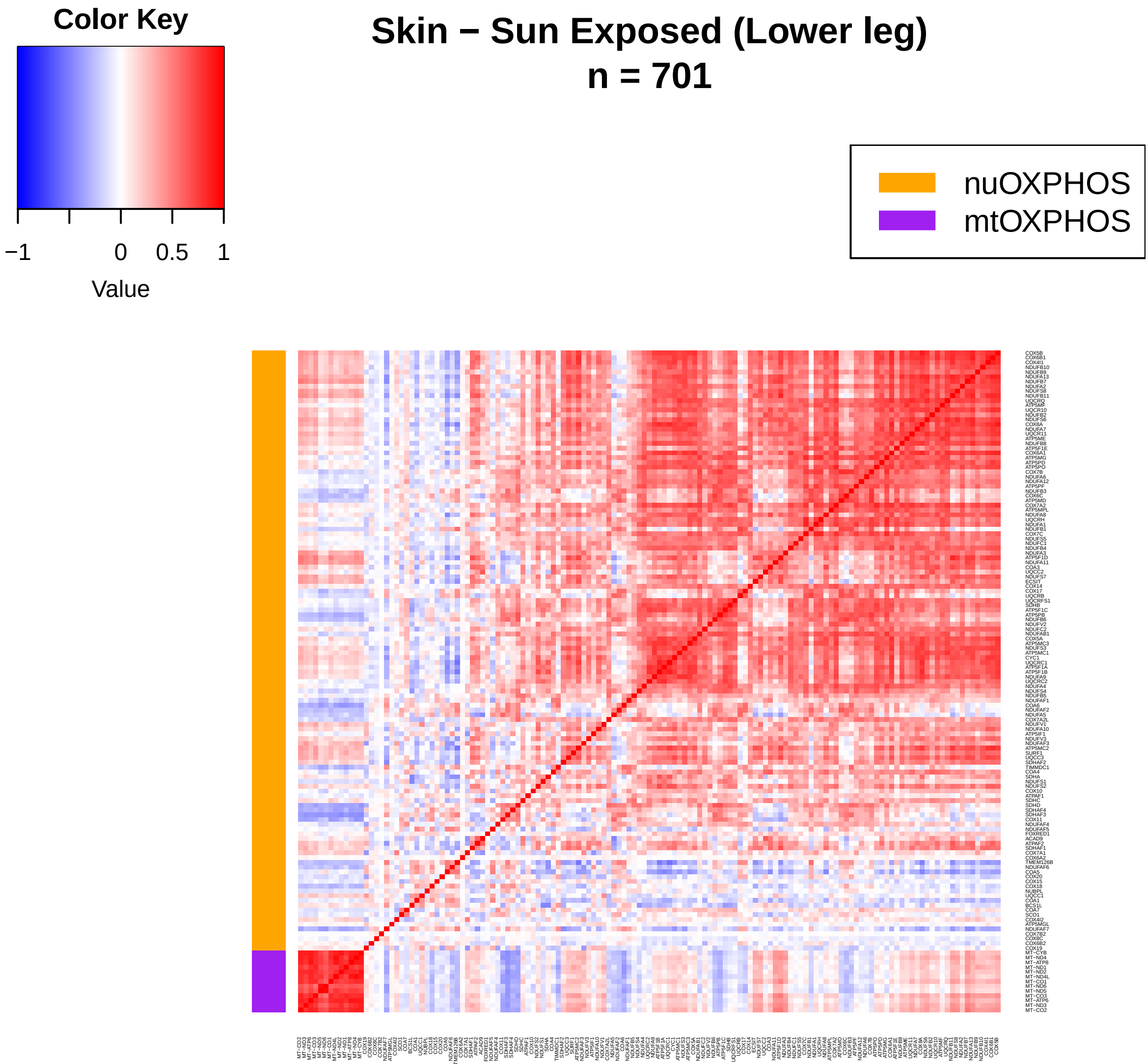
Skin − Sun Exposed (Lower leg)
n = 701
Color Key
−1
0
0.5
1
Value
nuOXPHOS
mtOXPHOS

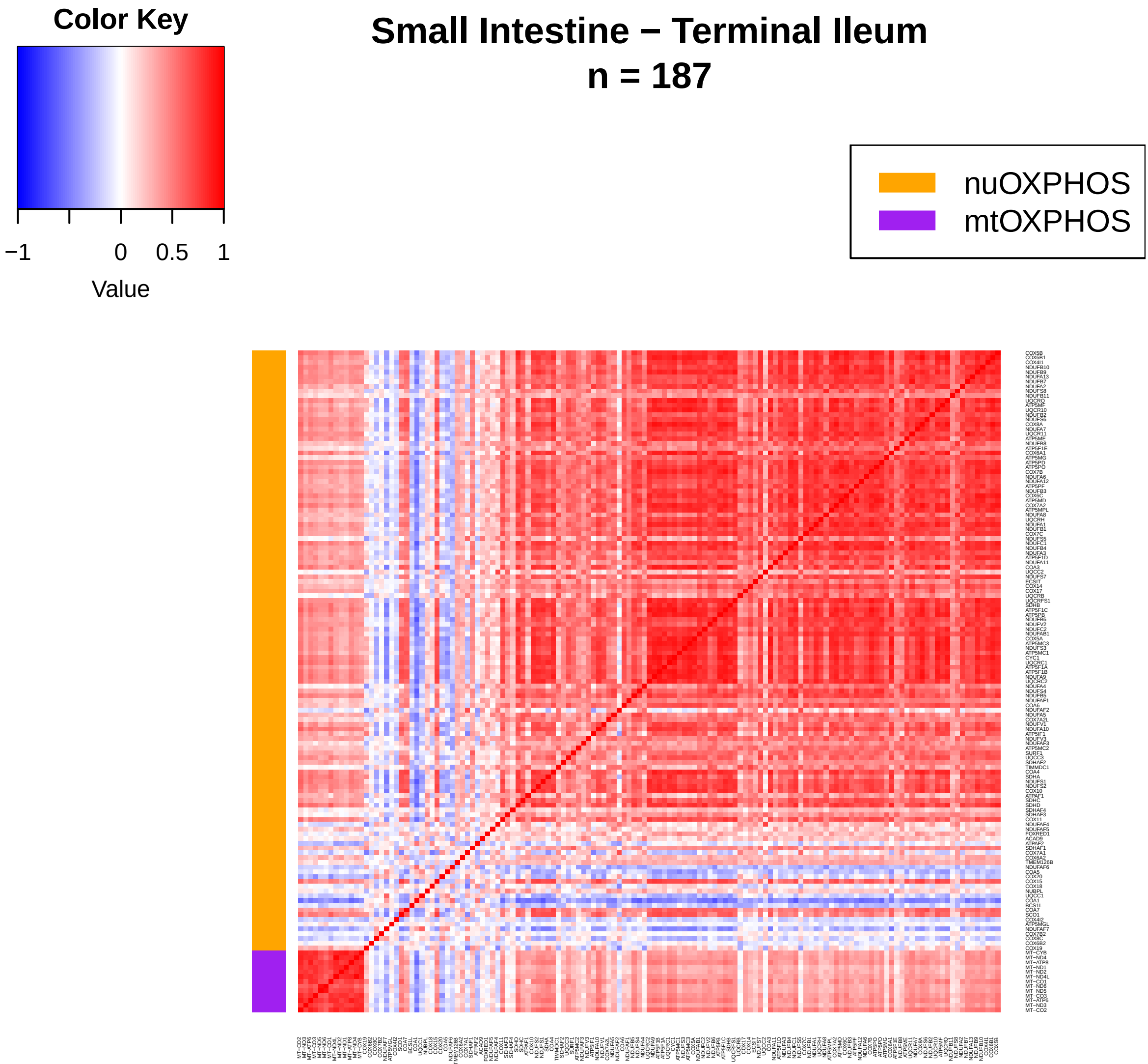
Color Key
−1
0
0.5
1
Value
Small Intestine − Terminal Ileum
n = 187
nuOXPHOS
mtOXPHOS

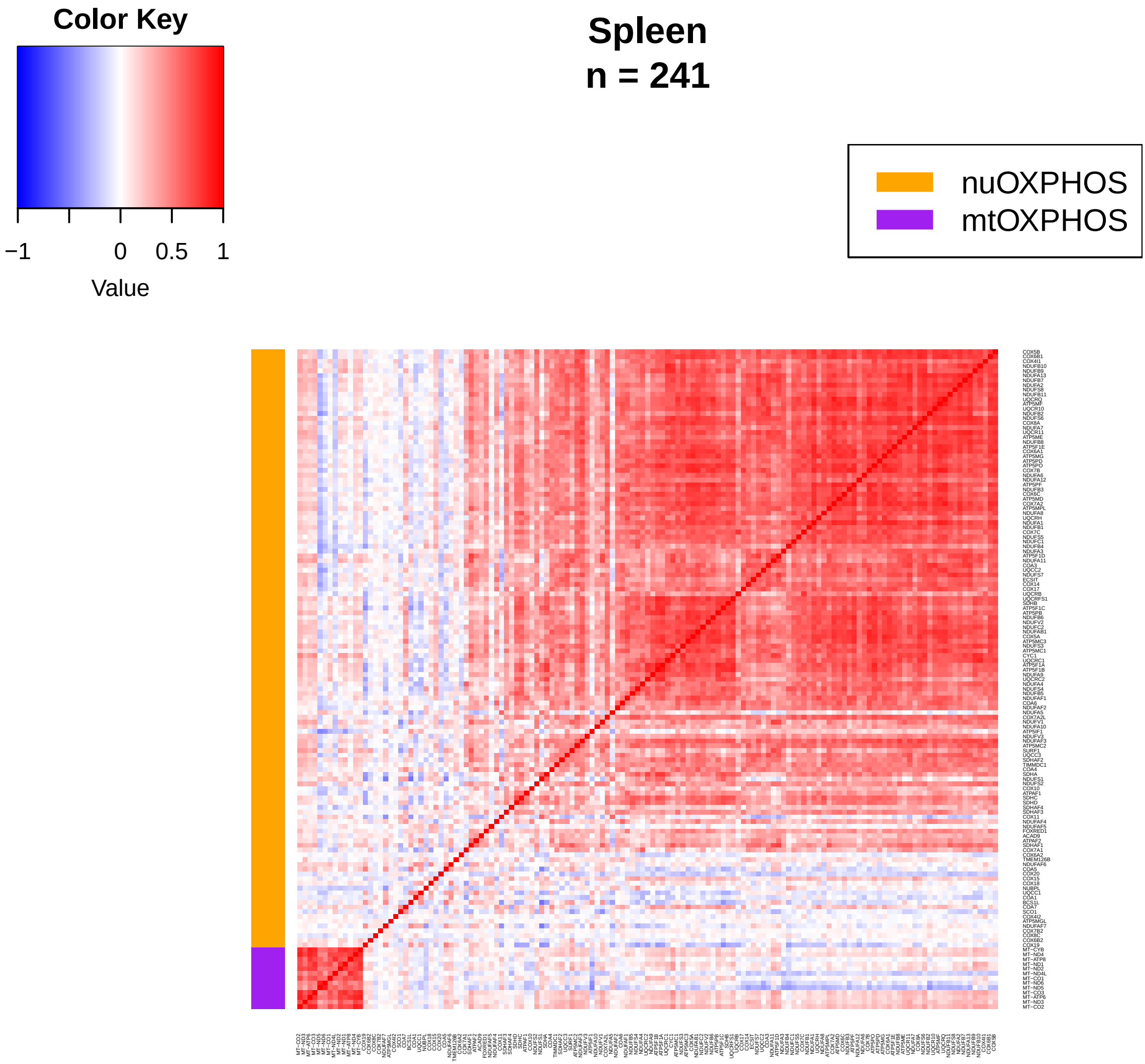
Color Key
−1
0
0.5
1
Value
Spleen
n = 241
nuOXPHOS
mtOXPHOS

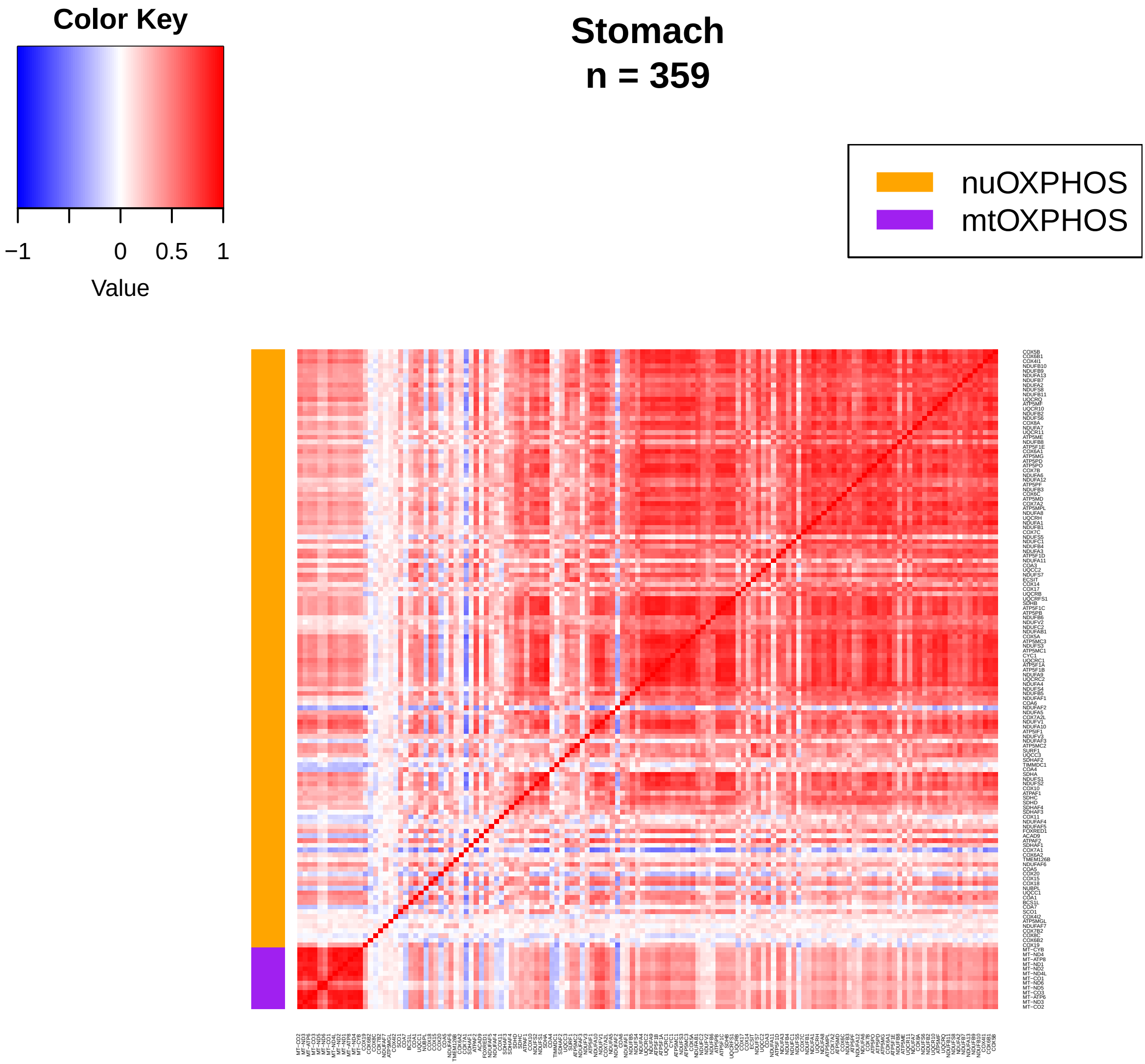

Color Key
−1
0
0.5
1
Value
Stomach
n = 359
nuOXPHOS
mtOXPHOS

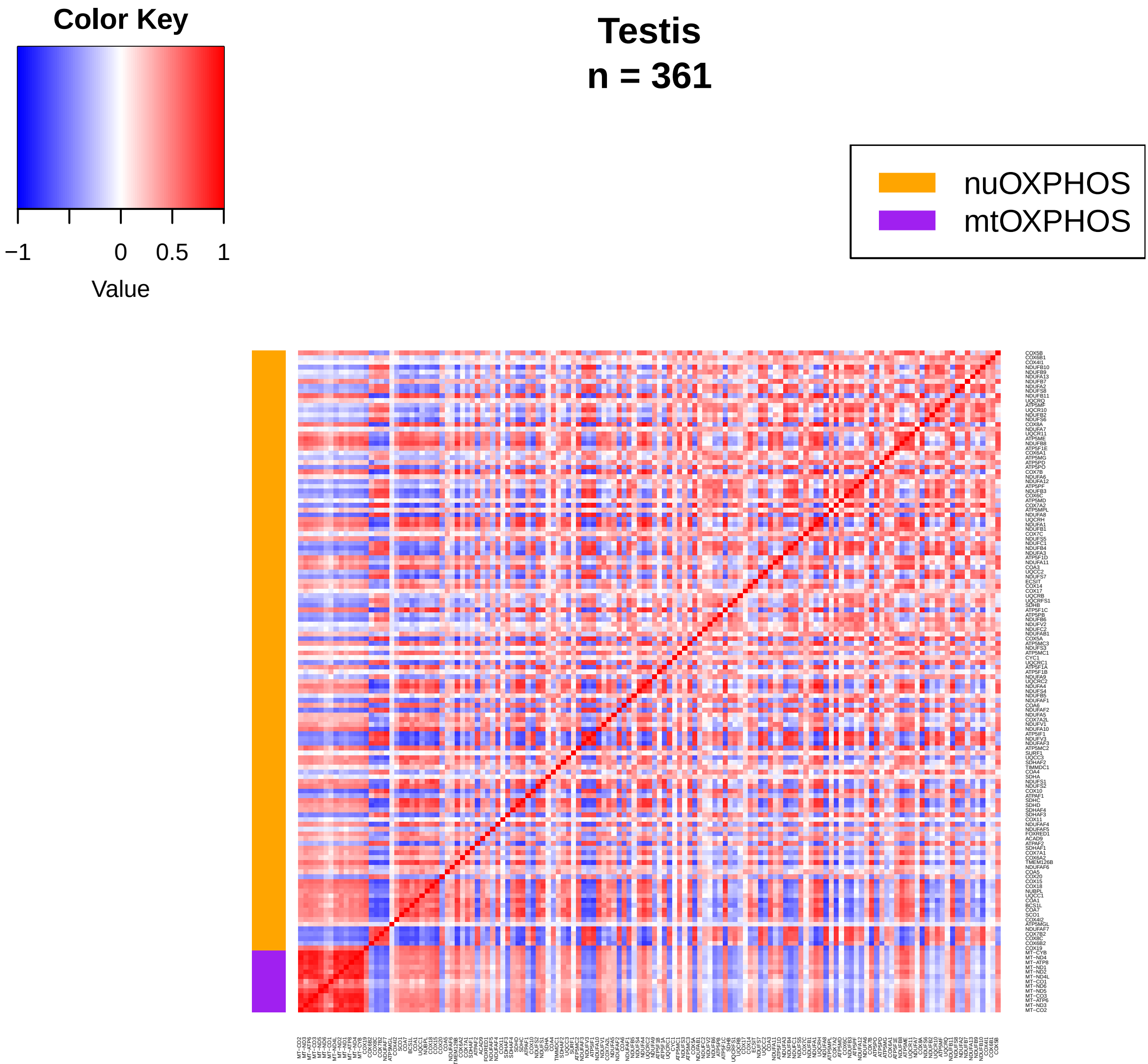
Color Key
−1
0
0.5
1
Value
Testis
n = 361
nuOXPHOS
mtOXPHOS

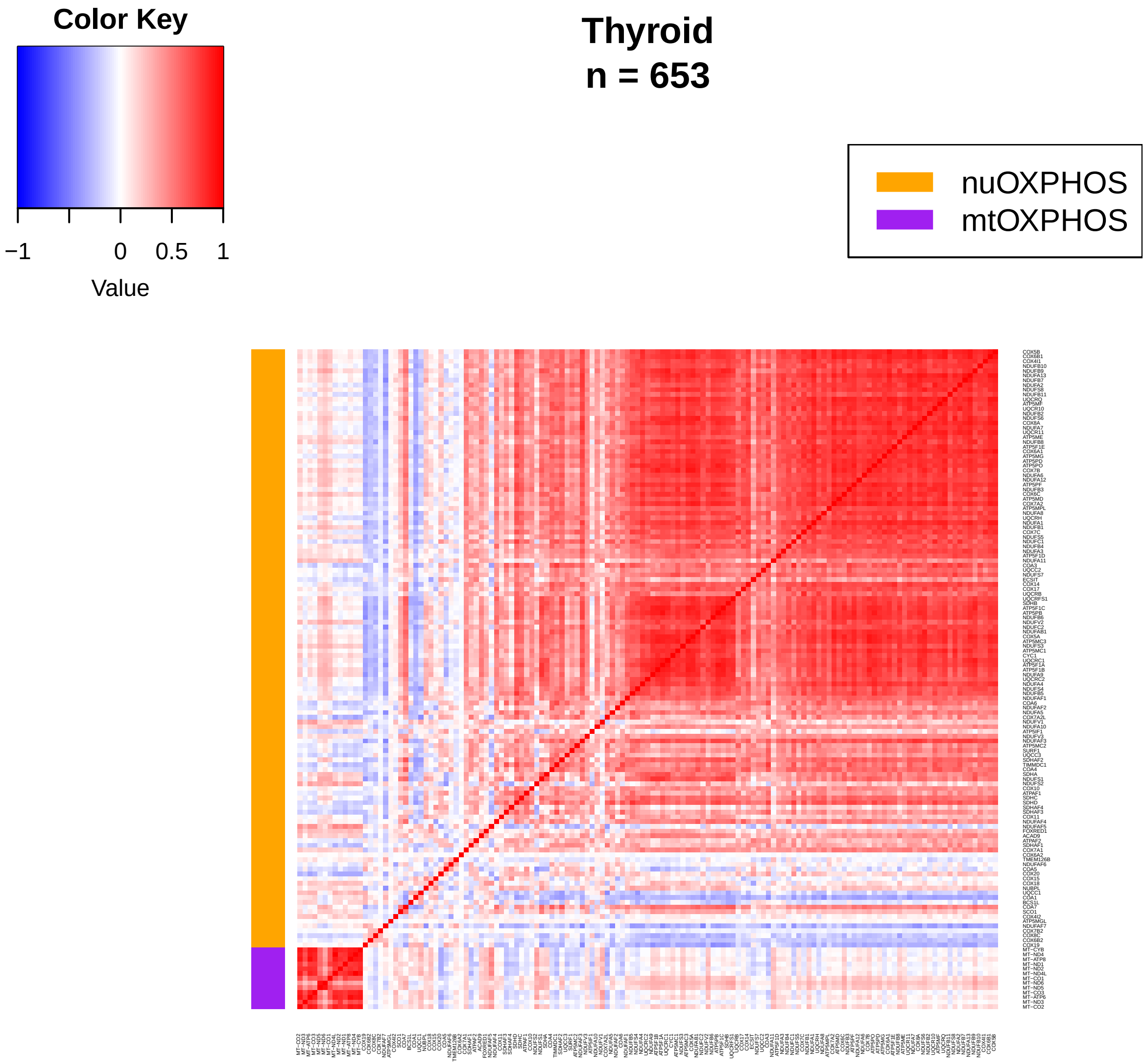
Color Key
−1
0
0.5
1
Value
Thyroid
n = 653
nuOXPHOS
mtOXPHOS

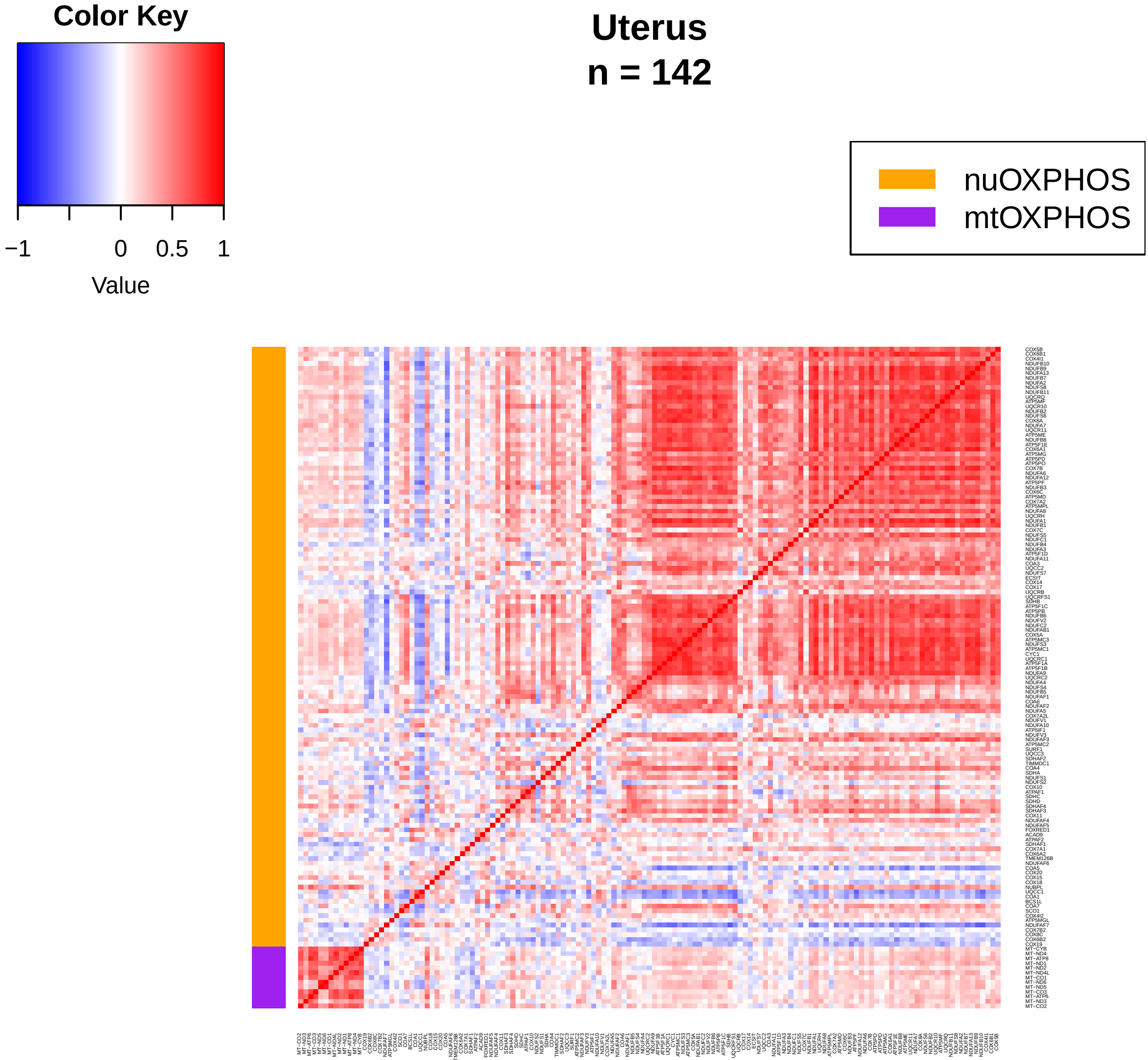
Color Key
−1
0
0.5
1
Value
Uterus
n = 142
nuOXPHOS
mtOXPHOS

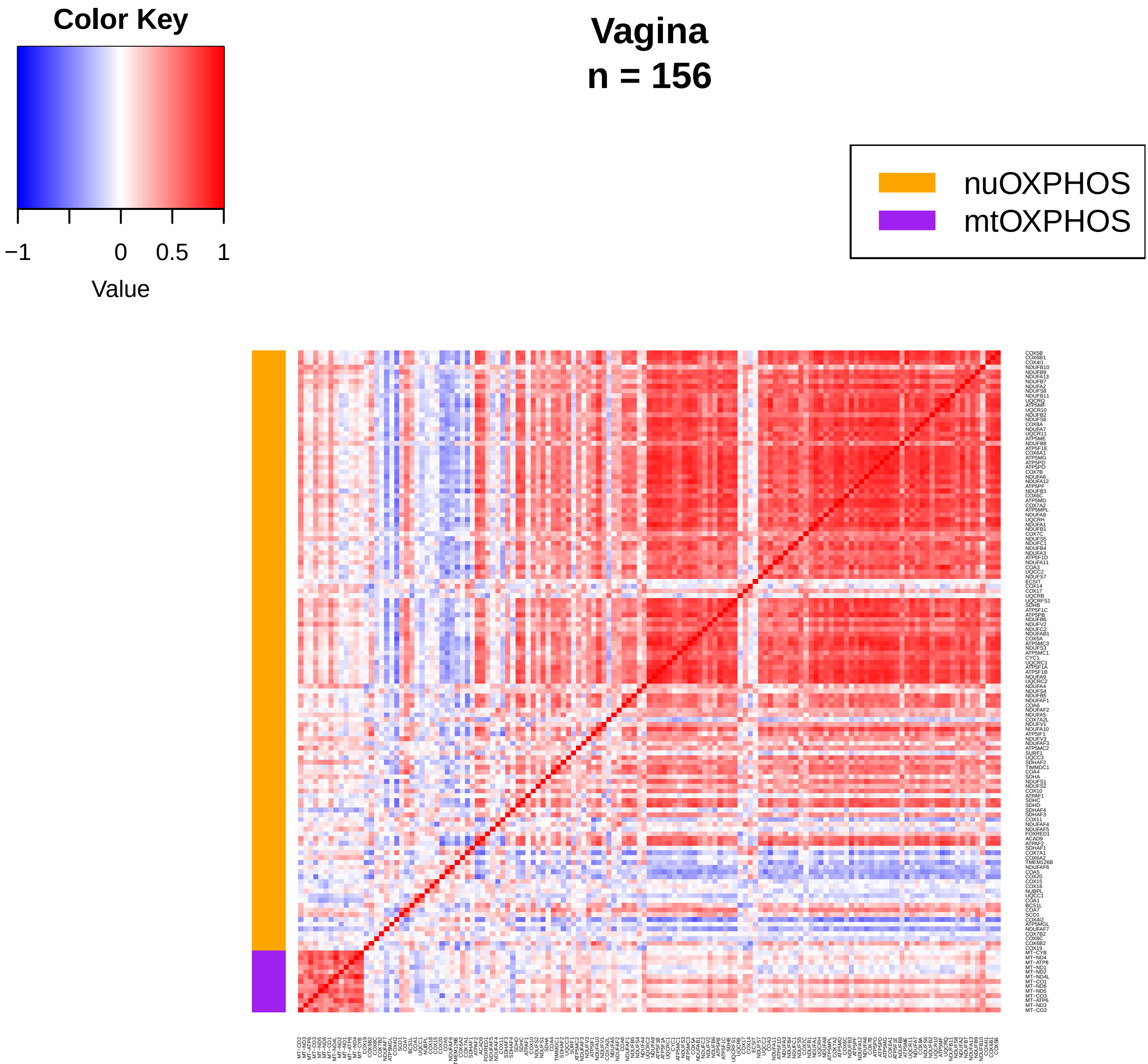
Color Key
−1
0
0.5
1
Value
Vagina
n = 156
nuOXPHOS
mtOXPHOS

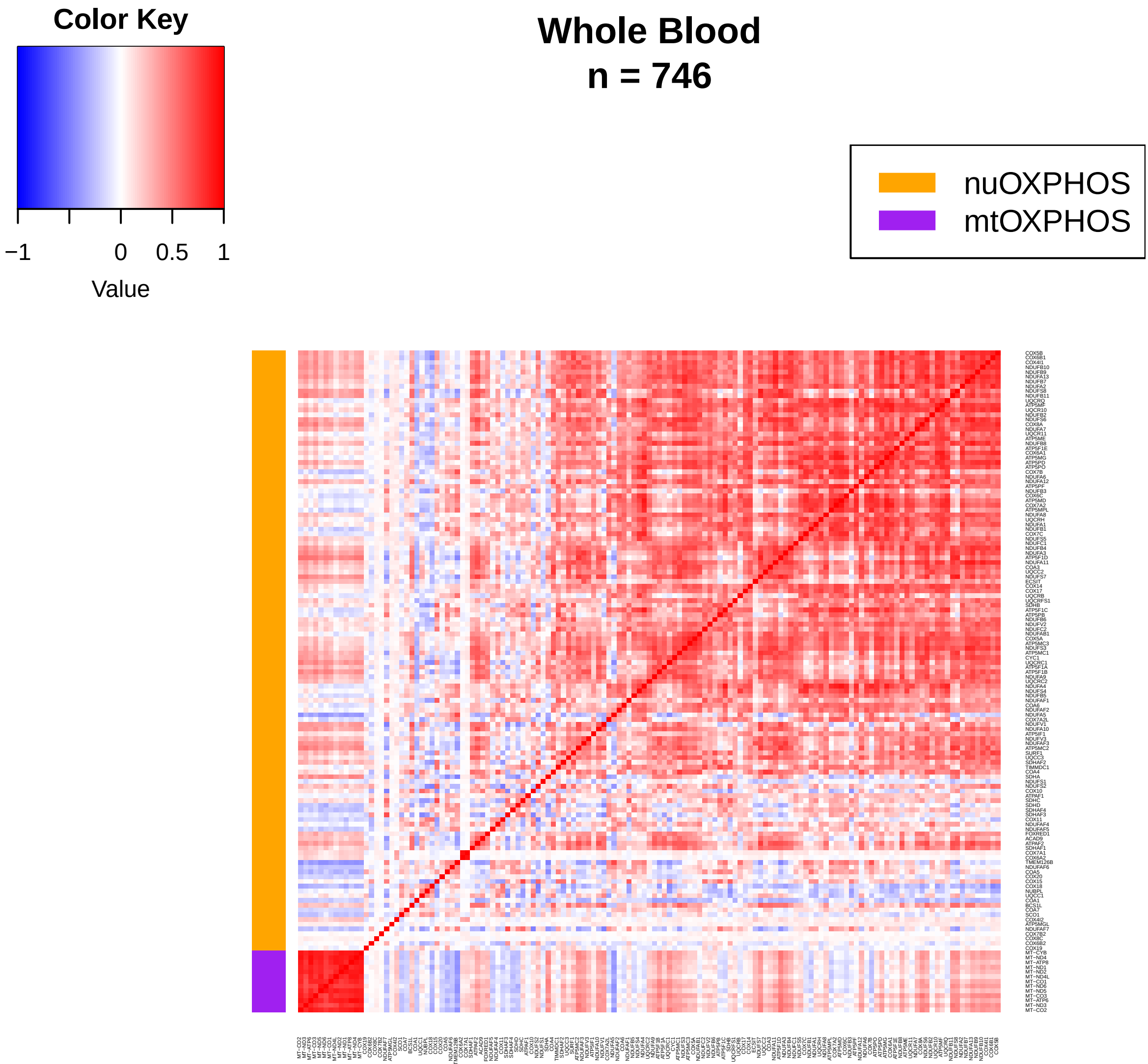
Color Key
−1
0
0.5
1
Value
Whole Blood
n = 746
nuOXPHOS
mtOXPHOS
