# Supplementary material for: Malignancy and NF-κB signalling strengthen coordination between expression of mitochondrial and nuclear-encoded oxidative phosphorylation genes
Source: Genome Biol. 2021 Dec 2;22:328. doi: 10.1186/s13059-021-02541-6 (PMC8638269; doi:10.1186/s13059-021-02541-6)
Supplement: Supplementary file 4 — Additional File 4. TCGA matched tumour heatmaps. mtOXPHOS-nuOXPHOS expression heatmaps for matched tumour samples for 14 cancer types. [file 13059_2021_2541_MOESM4_ESM.pdf]

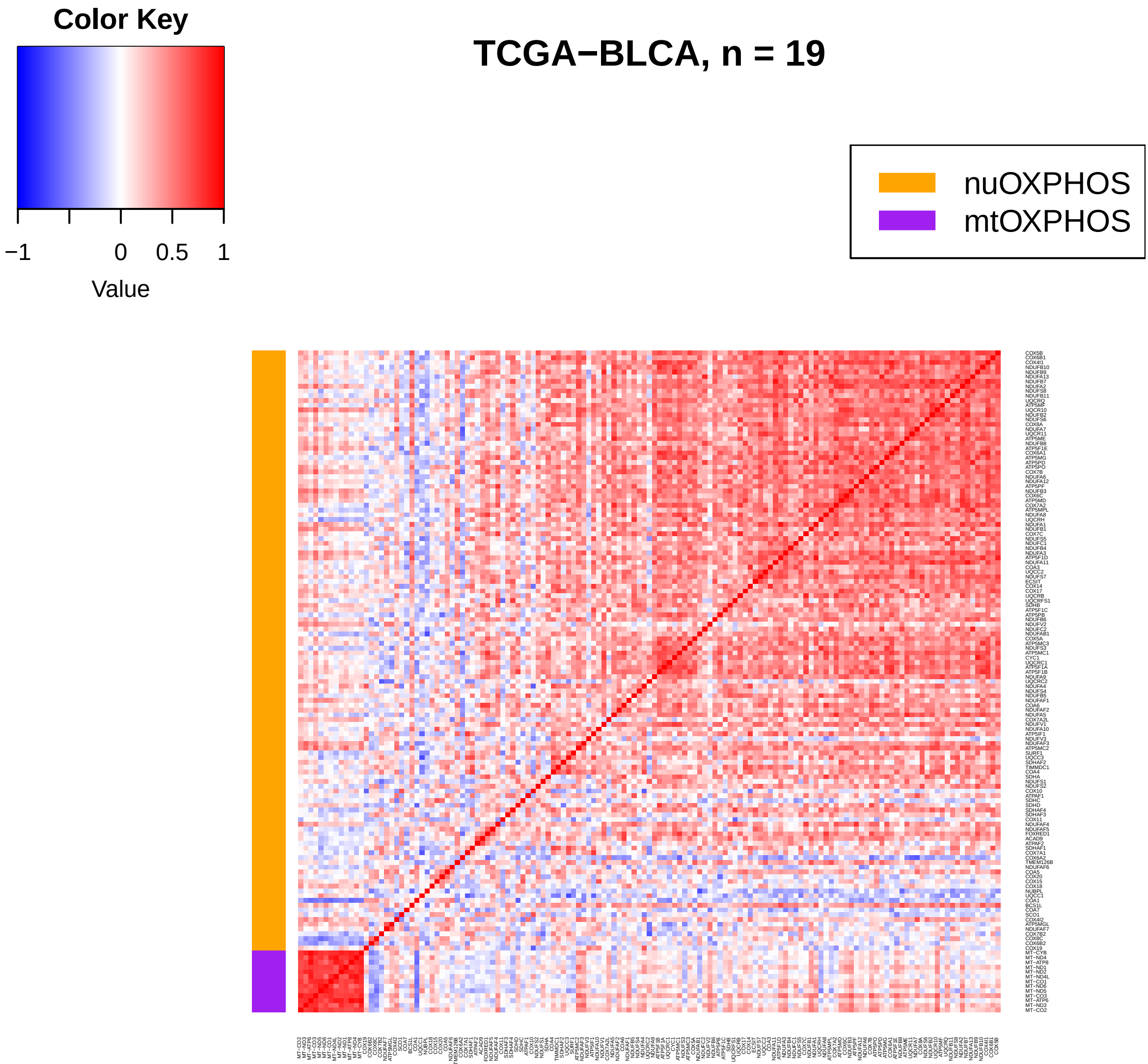

Color Key
−1
0
0.5
1
Value
TCGA−BLCA, n = 19
nuOXPHOS
mtOXPHOS

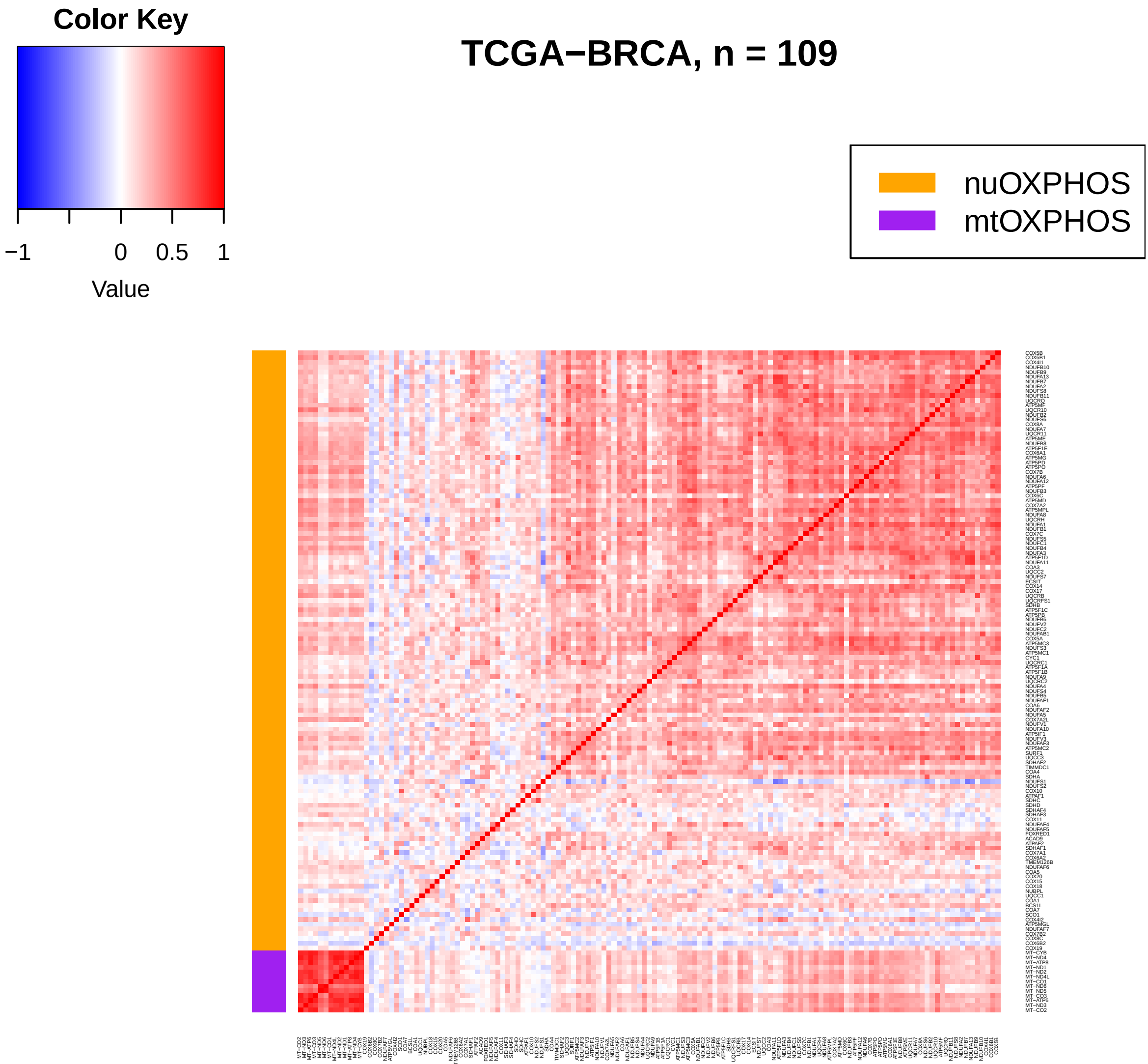
Color Key
−1
0
0.5
1
Value
TCGA−BRCA, n = 109
nuOXPHOS
mtOXPHOS

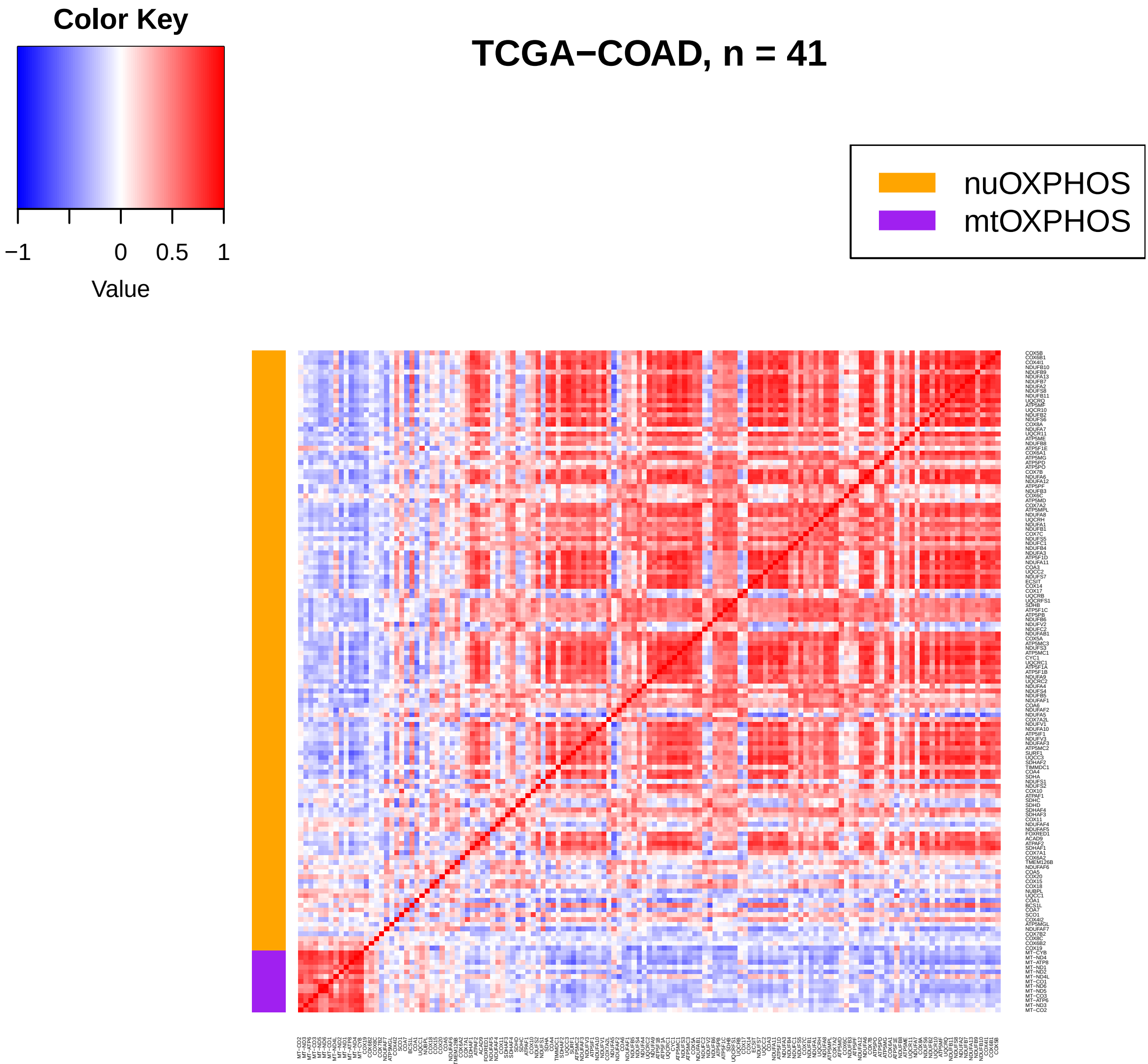
Color Key
−1
0
0.5
1
Value
TCGA−COAD, n = 41
nuOXPHOS
mtOXPHOS

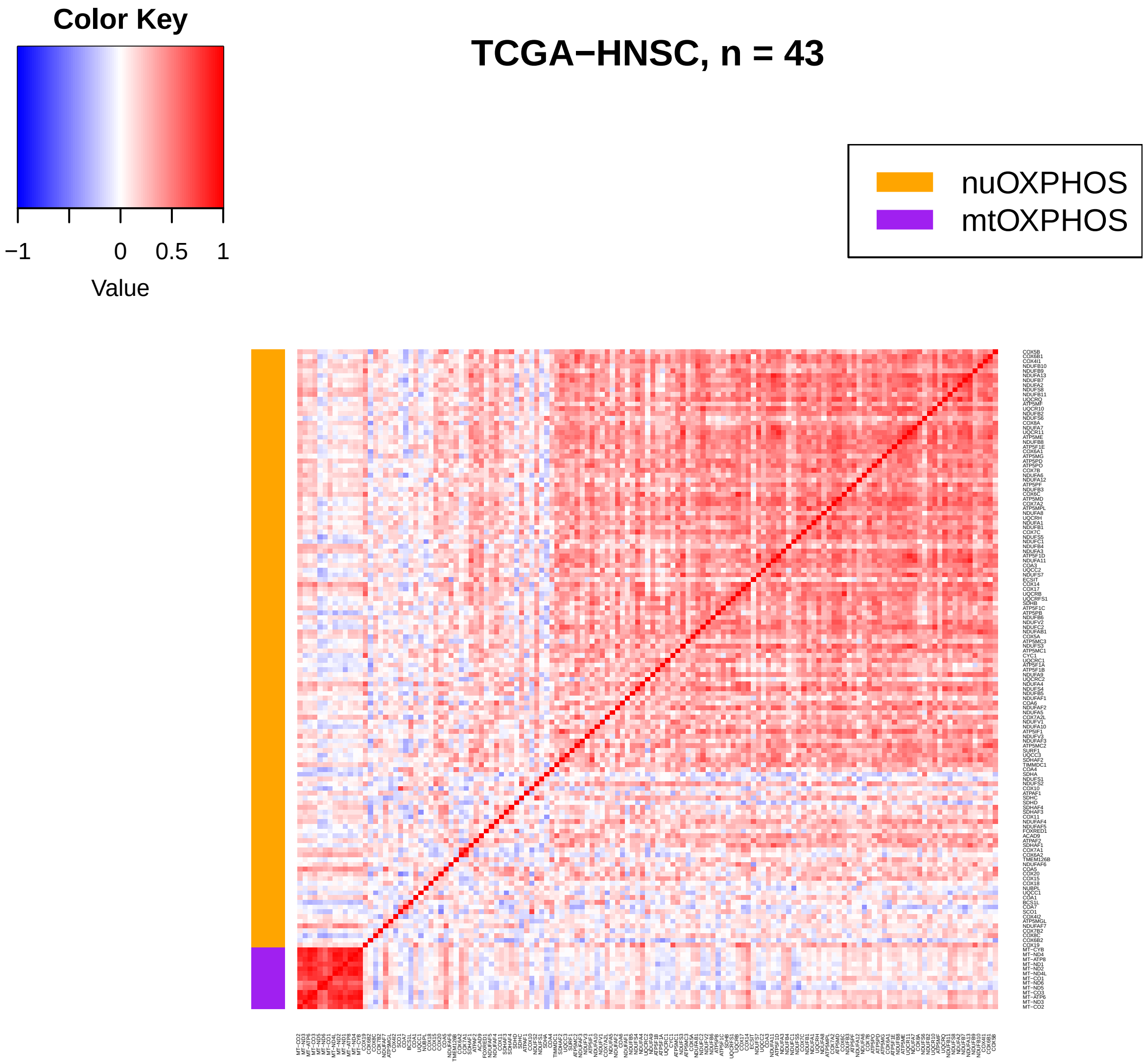

Color Key
−1
0
0.5
1
Value
TCGA−HNSC, n = 43
nuOXPHOS
mtOXPHOS

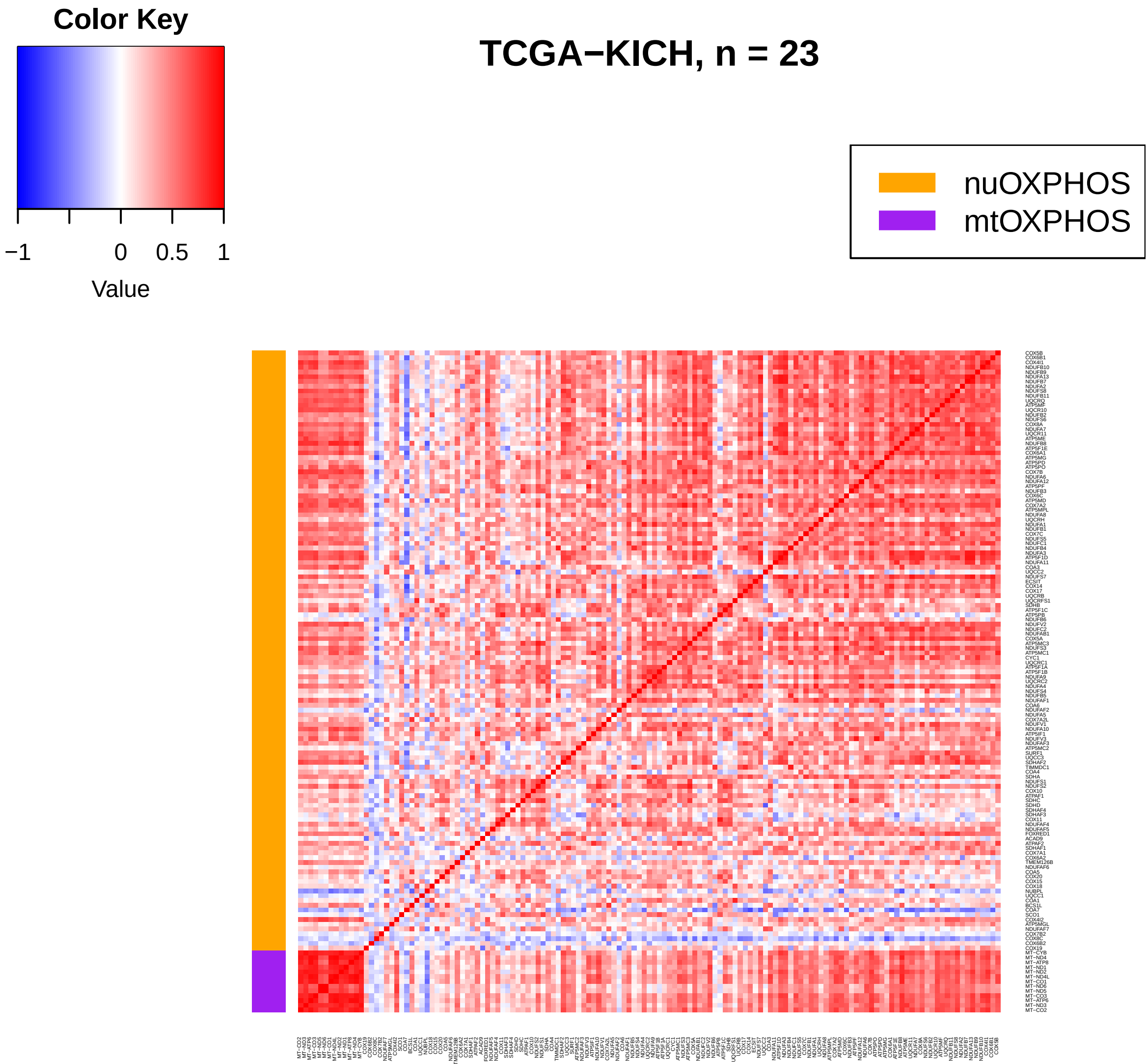
Color Key
−1
0
0.5
1
Value
TCGA−KICH, n = 23
nuOXPHOS
mtOXPHOS

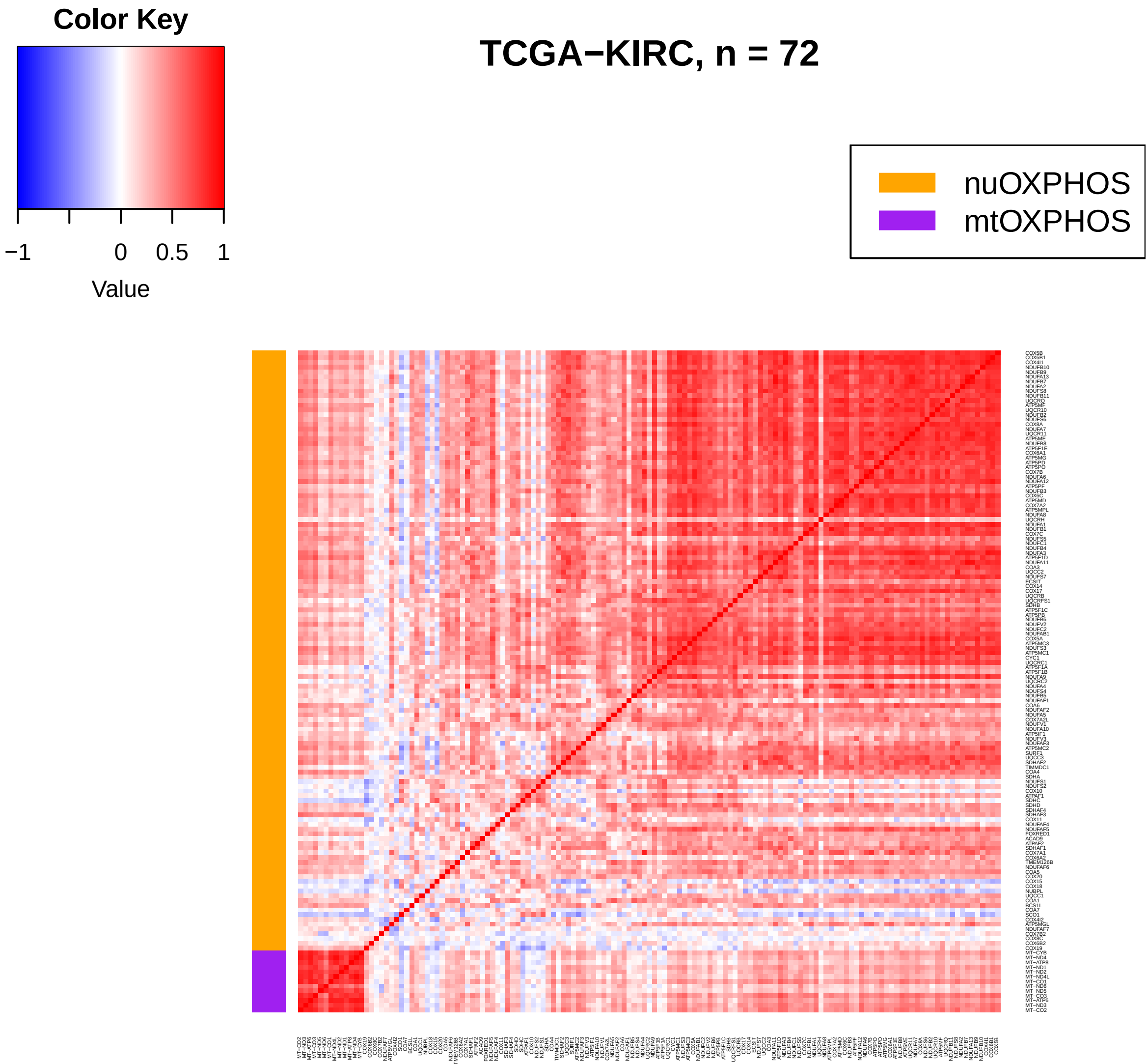
Color Key
−1
0
0.5
1
Value
TCGA−KIRC, n = 72
nuOXPHOS
mtOXPHOS

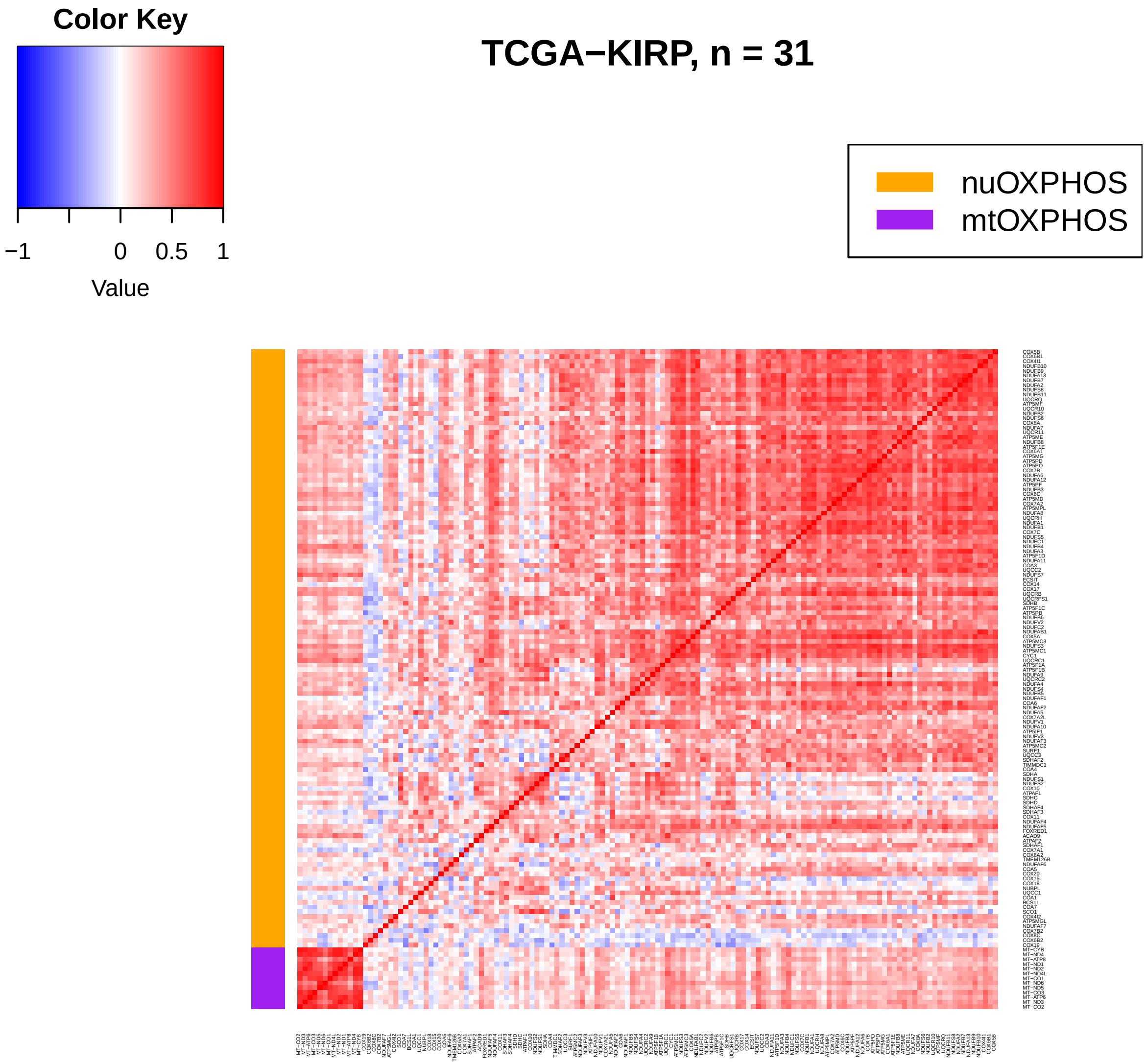
Color Key
−1
0
0.5
1
Value
TCGA−KIRP, n = 31
nuOXPHOS
mtOXPHOS

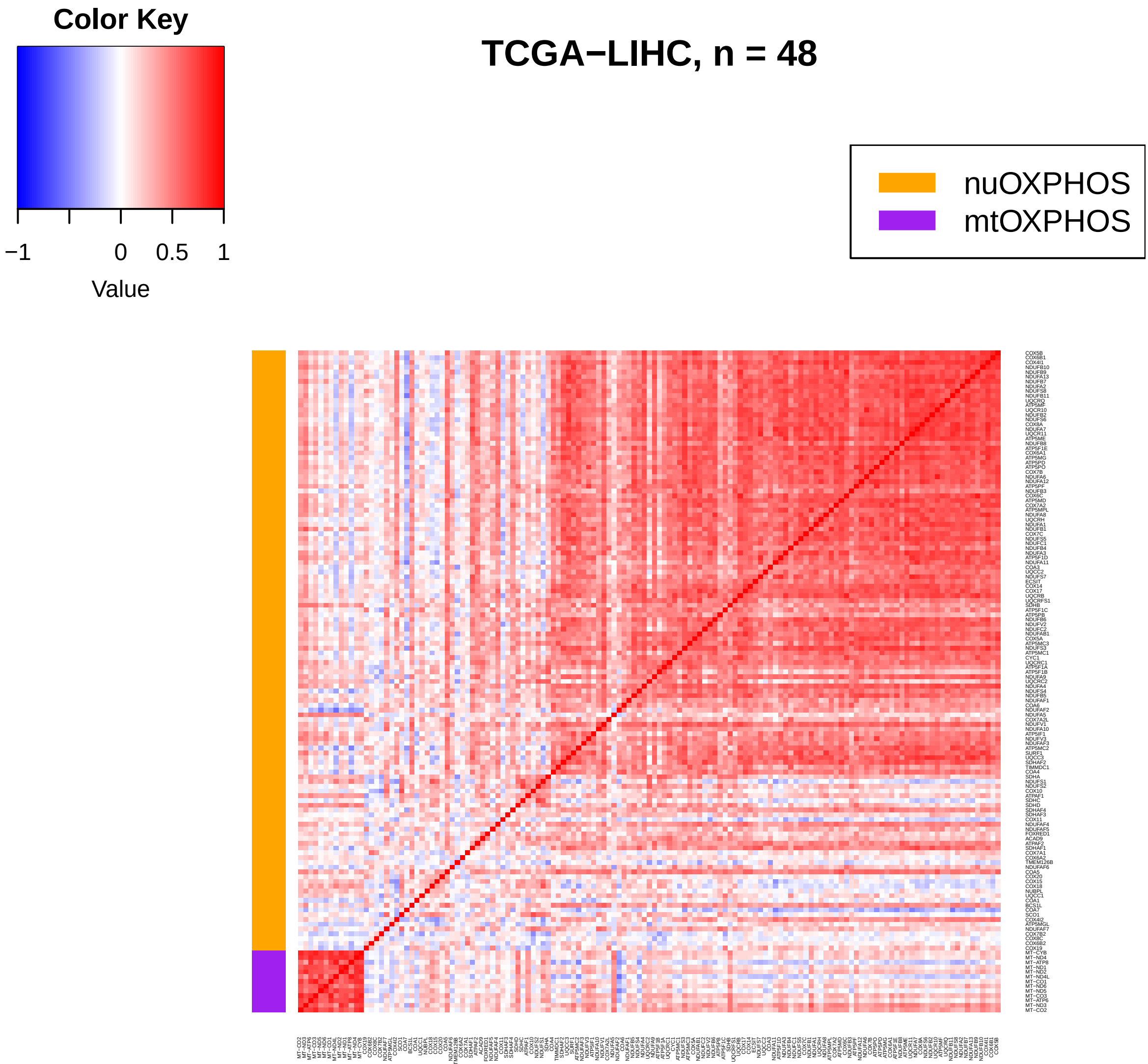

Color Key
−1
0
0.5
1
Value
TCGA−LIHC, n = 48
nuOXPHOS
mtOXPHOS

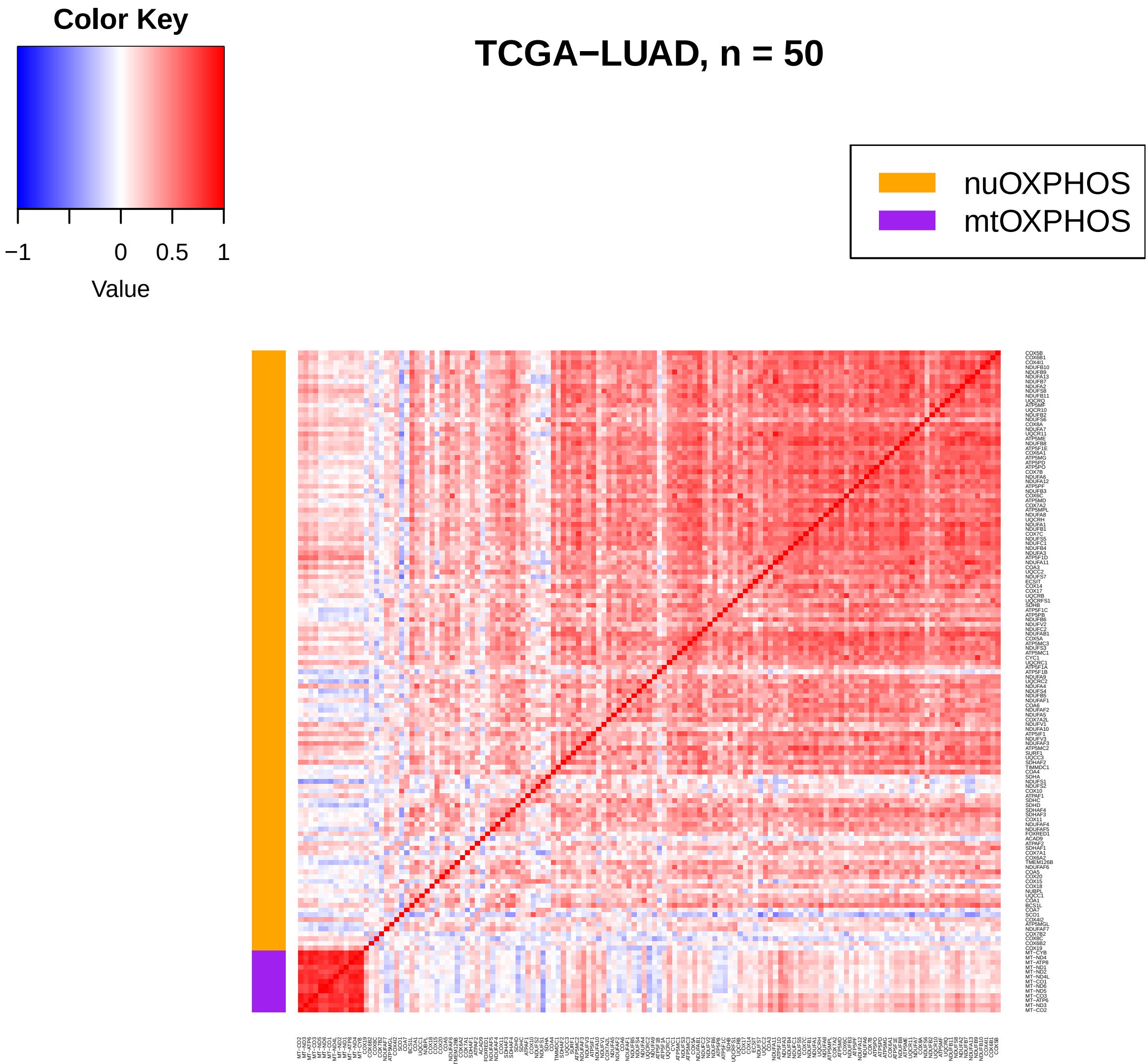
Color Key
−1
0
0.5
1
Value
TCGA−LUAD, n = 50
nuOXPHOS
mtOXPHOS

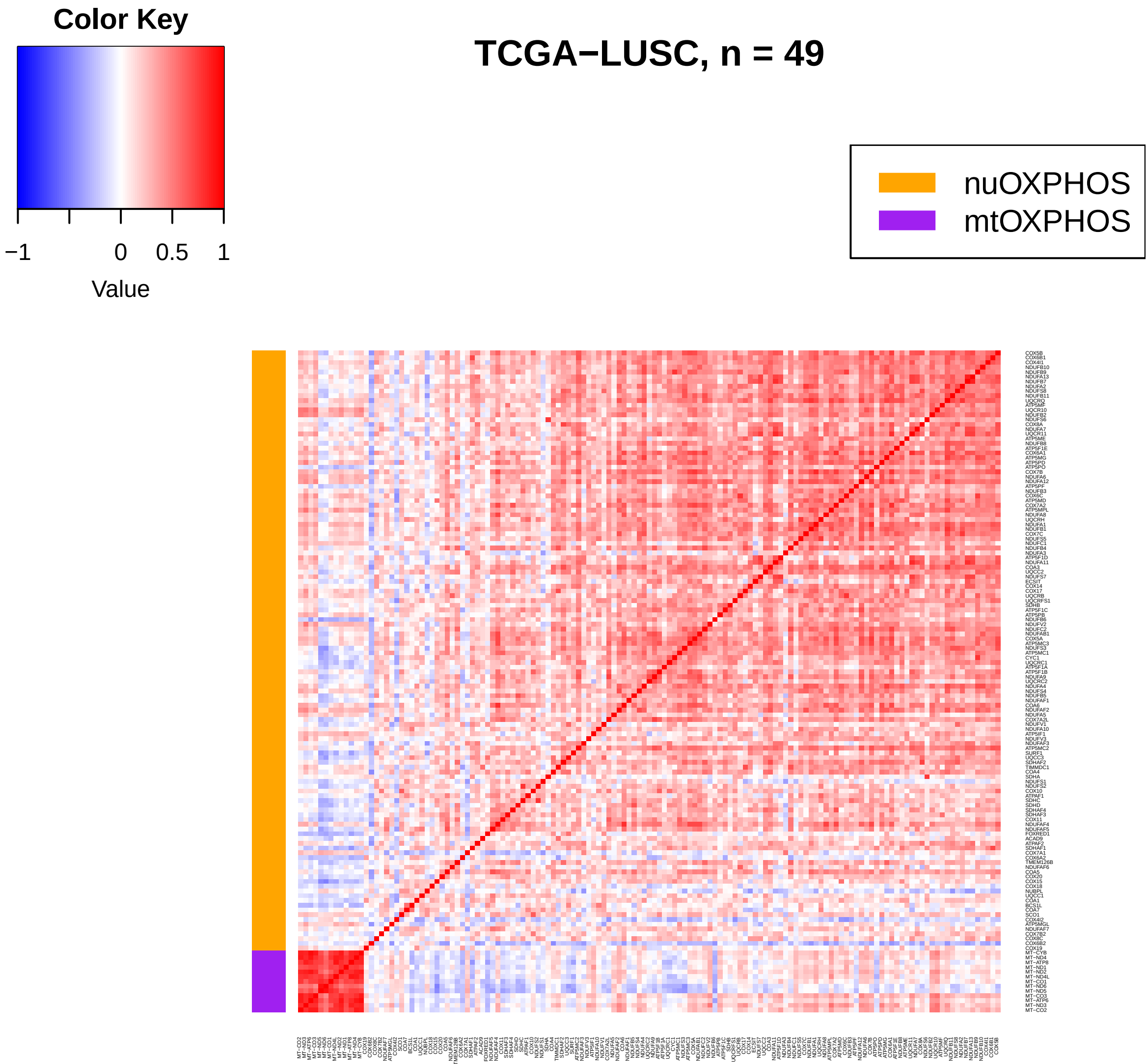
Color Key
−1
0
0.5
1
Value
TCGA−LUSC, n = 49
nuOXPHOS
mtOXPHOS

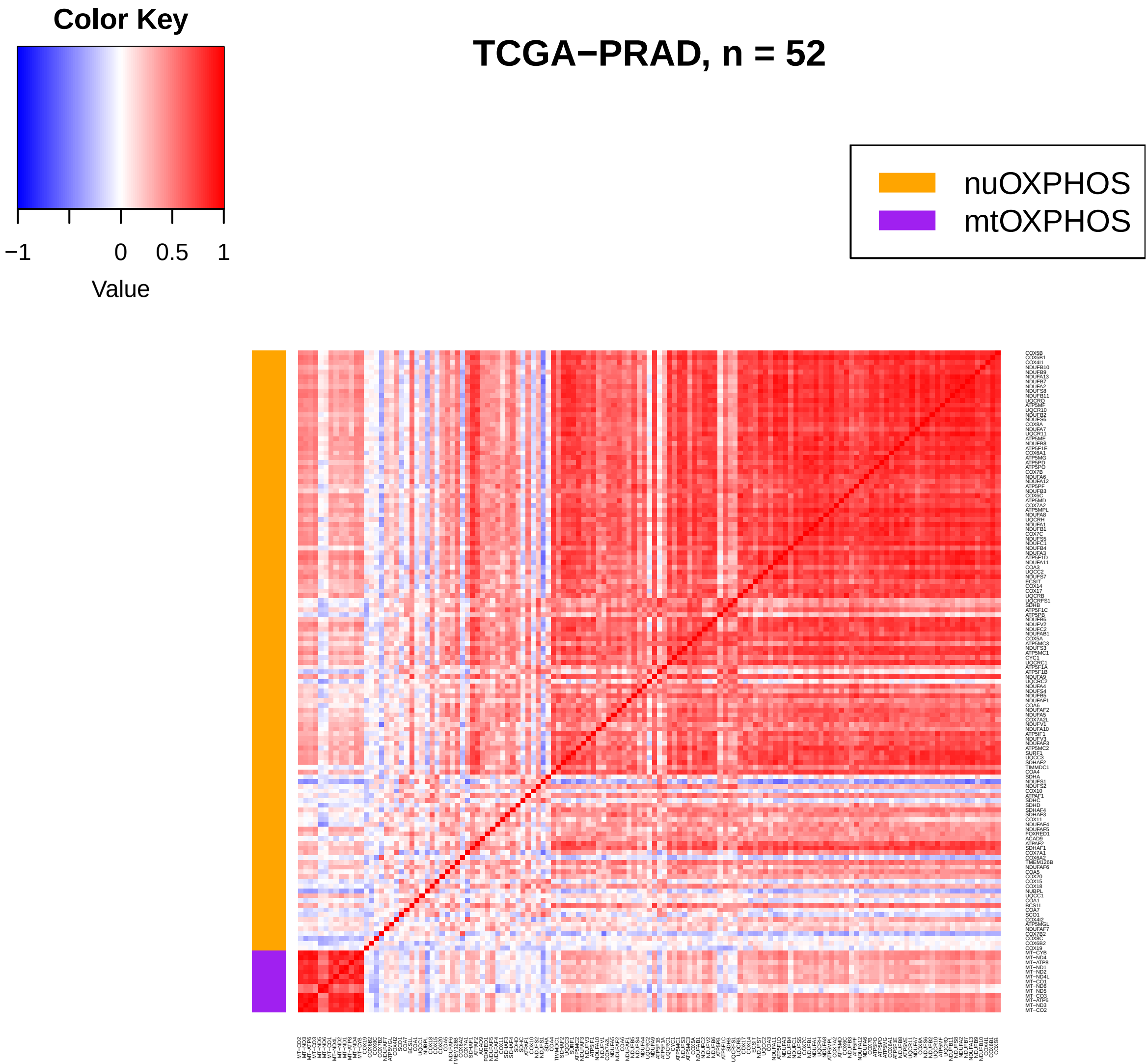
Color Key
−1
0
0.5
1
Value
TCGA−PRAD, n = 52
nuOXPHOS
mtOXPHOS

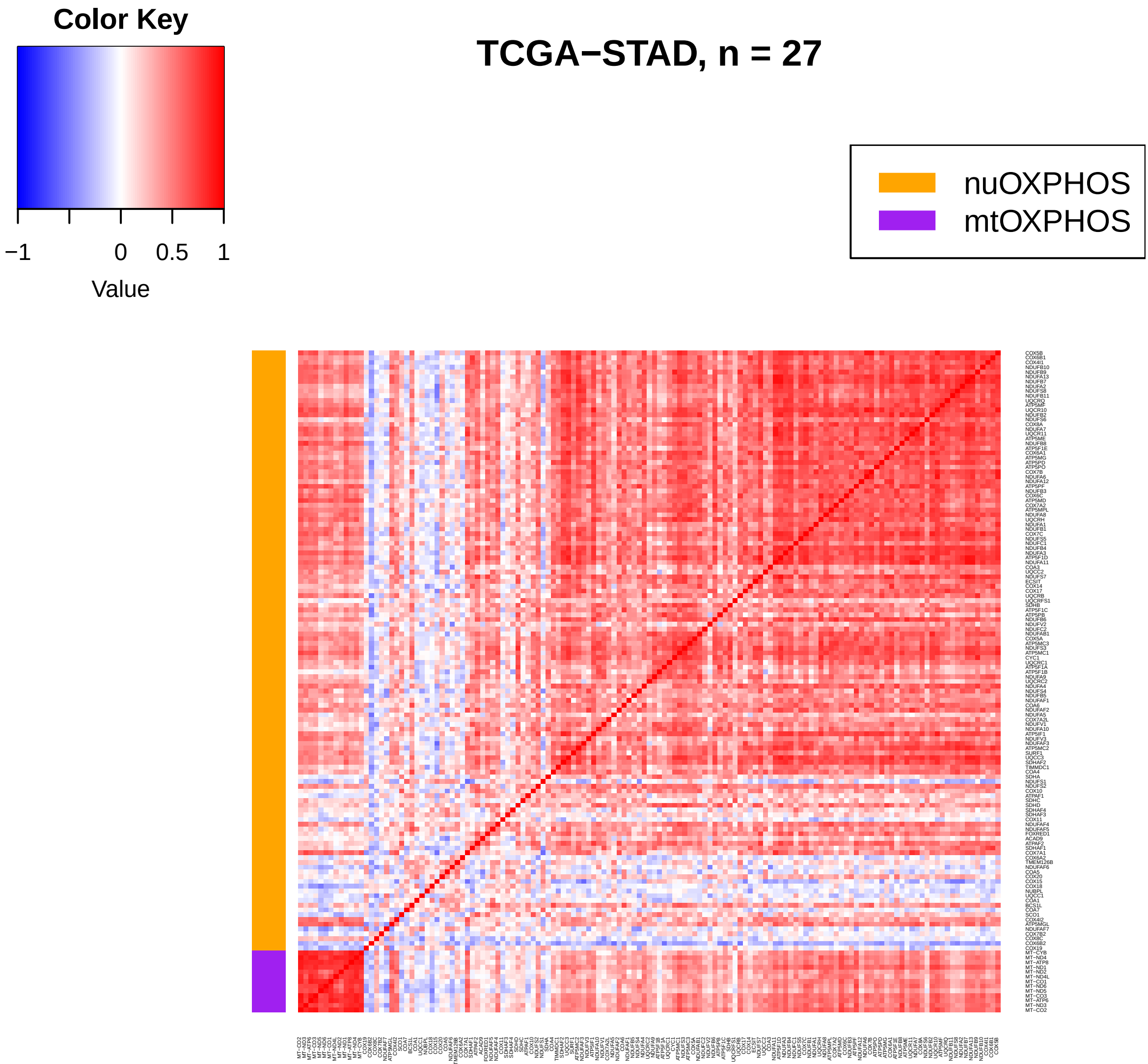
Color Key
−1
0
0.5
1
Value
TCGA−STAD, n = 27
nuOXPHOS
mtOXPHOS

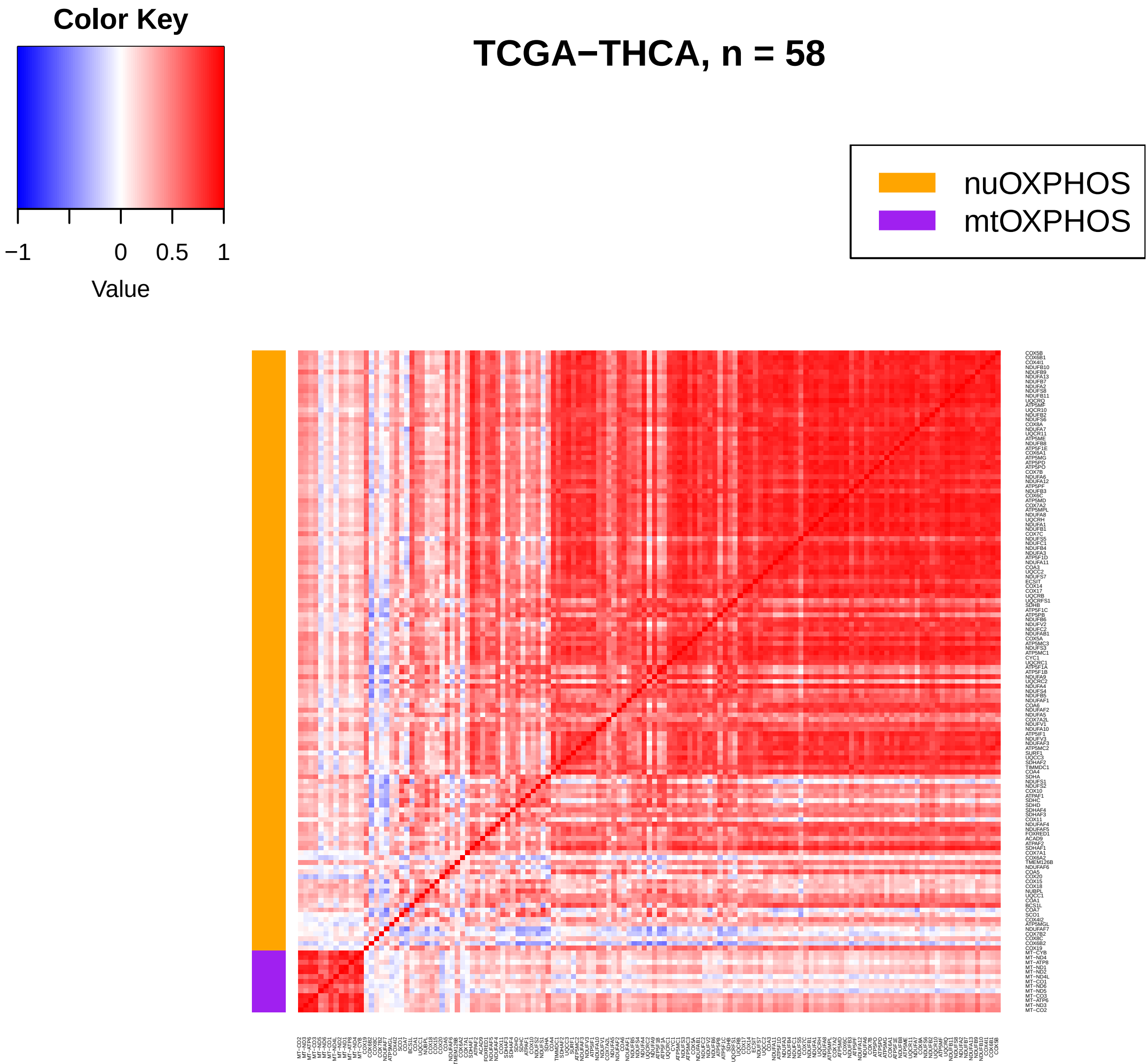
Color Key
−1
0
0.5
1
Value
TCGA−THCA, n = 58
nuOXPHOS
mtOXPHOS

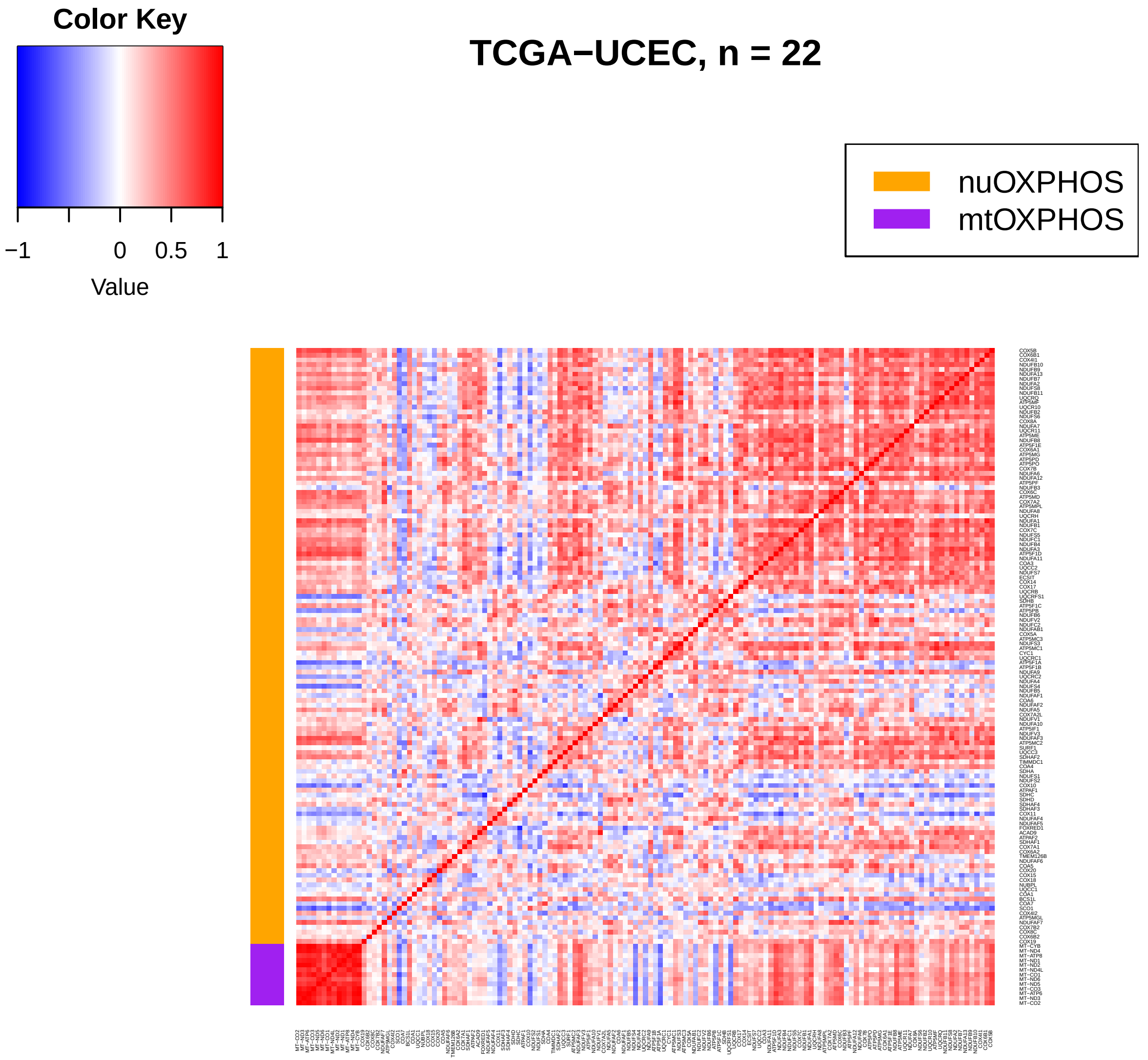
Color Key
−1
0
0.5
1
Value
TCGA−UCEC, n = 22
nuOXPHOS
mtOXPHOS
